# Supplementary material for: Association of pregnancy complications/risk factors with the development of future long-term health conditions in women: overarching protocol for umbrella reviews
Source: BMJ Open. 2022 Dec 29;12(12):e066476. doi: 10.1136/bmjopen-2022-066476 (PMC9806074; doi:10.1136/bmjopen-2022-066476)
Supplement: Supplementary data [file bmjopen-2022-066476supp001.pdf]

The association of pregnancy complications/risk factors with the development of future long-term health conditions in women: overarching protocol for umbrella reviews

Appendix/Supplementary material

- Appendix Table 1: PRISMA-P (Preferred Reporting Items for Systematic review and Meta-Analysis Protocols) 2015 checklist: recommended items to address in a systematic review protocol).
- Appendix Table 2: Definitions of pregnancy complications and risk factors
- Appendix Table 3: Search terms for pregnancy complications/risk factors (exposure)
- Appendix Table 4: Search terms for long-term health conditions (outcomes)
- Appendix Table 5.1 Search strategy- Medline Database-Autoimmune condition and pregnancy complications
- Appendix Table 5.2 search strategy- Medline database- Cancer and pregnancy complications
- Appendix Table 5.3 search strategy- Medline database- Functional conditions and pregnancy complications
- Appendix Table 5.4 search strategy- Medline database- Mental health condition and pregnancy complications
- Appendix Table 5.5 search strategy- Medline database- Metabolic conditions and pregnancy complications

Appendix Table 1: PRISMA-P (Preferred Reporting Items for Systematic review and Meta-Analysis Protocols) 2015 checklist: recommended items to address in a systematic review protocol

PRISMA-P (Preferred Reporting Items for Systematic review and Meta-Analysis Protocols) 2015 checklist: recommended items to address in a systematic review protocol*

Section and topic	Item No	Checklist item
**ADMINISTRATIVE INFORMATION**		
Title:		
Identification	1a	Identify the report as a protocol of a systematic review-**Page 4 lines 143-149**
Update	1b	If the protocol is for an update of a previous systematic review, identify as such-**NA**
Registration	2	If registered, provide the name of the registry (such as PROSPERO) and registration number-**Page 4 line 147**
Authors:		
Contact	3a	Provide name, institutional affiliation, e-mail address of all protocol authors; provide physical mailing address of corresponding author-**Page 1 and 2 lines 4-47**
Contributions	3b	Describe contributions of protocol authors and identify the guarantor of the review-**Page 10 lines 347-352**

Singh M, *et al. BMJ Open* 2022; 12:e066476. doi: 10.1136/bmjopen-2022-066476

Amendments	4	If the protocol represents an amendment of a previously completed or published protocol, identify as such and list changes; otherwise, state plan for documenting important protocol amendments-**NA**
Support:		
Sources	5a	Indicate sources of financial or other support for the review-**Page 10, lines 354-359**
Sponsor	5b	Provide name for the review funder and/or sponsor- **Page 10, line 354**
Role of sponsor or funder	5c	Describe roles of funder(s), sponsor(s), and/or institution(s), if any, in developing the protocol-**Page 10, lines 359**
**INTRODUCTION**		
Rationale	6	Describe the rationale for the review in the context of what is already known **Page 4 and 5 lines 94-142**
Objectives	7	Provide an explicit statement of the question(s) the review will address with reference to participants, interventions, comparators, and outcomes (PICO)- **Page 4 lines 161-199**
**METHODS**		
Eligibility criteria	8	Specify the study characteristics (such as PICO, study design, setting, time frame) and report characteristics (such as years considered, language, publication status) to be used as criteria for eligibility for the review-**Page 5 lines 168-200**
Information sources	9	Describe all intended information sources (such as electronic databases, contact with study authors, trial registers or other grey literature sources) with planned dates of coverage-**Page 7 lines 201-216**
Search strategy	10	Present draft of search strategy to be used for at least one electronic database, including planned limits, such that it could be repeated **Appendix table 5.1-5.5**
Study records:		
Data management	11a	Describe the mechanism(s) that will be used to manage records and data throughout the review-**Page 7 lines 217-218**
Selection process	11b	State the process that will be used for selecting studies (such as two independent reviewers) through each phase of the review (that is, screening, eligibility and inclusion in meta-analysis)-**Page 7 line 218-226**
Data collection process	11c	Describe planned method of extracting data from reports (such as piloting forms, done independently, in duplicate), any processes for obtaining and confirming data from investigators-**Page 8 and 9 lines 267-304**
Data items	12	List and define all variables for which data will be sought (such as PICO items, funding sources), any pre-planned data assumptions and simplifications- **Appendix table 1**
Outcomes and prioritization	13	List and define all outcomes for which data will be sought, including prioritization of main and additional outcomes, with rationale-**Table 2 main document**
Risk of bias in individual studies	14	Describe anticipated methods for assessing risk of bias of individual studies, including whether this will be done at the outcome or study level, or both; state how this information will be used in data synthesis-**Page 8 lines 238-248**
Data synthesis	15a	Describe criteria under which study data will be quantitatively synthesised- **Page 9 and 10 lines 306-327**
	15b	If data are appropriate for quantitative synthesis, describe planned summary measures, methods of handling data and methods of combining data from studies, including any planned exploration of consistency (such as I^2, Kendall's τ)- **Page 9 and 10 lines 306-327**

Singh M, *et al. BMJ Open* 2022; 12:e066476. doi: 10.1136/bmjopen-2022-066476

	15c	Describe any proposed additional analyses (such as sensitivity or subgroup analyses, meta-regression)- **Page 10 line 324**
	15d	If quantitative synthesis is not appropriate, describe the type of summary planned- **Page 10 line 308**
Meta-bias(es)	16	Specify any planned assessment of meta-bias(es) (such as publication bias across studies, selective reporting within studies**)-Page 10 line 325**
Confidence in cumulative evidence	17	Describe how the strength of the body of evidence will be assessed (such as GRADE)- **Page 10 line 326**

*** It is strongly recommended that this checklist be read in conjunction with the PRISMA-P Explanation and Elaboration (cite when available) for important clarification on the items. Amendments to a review protocol should be tracked and dated. The copyright for PRISMA-P (including checklist) is held by the PRISMA-P Group and is distributed under a Creative Commons Attribution Licence 4.0.**

From: Shamseer L, Moher D, Clarke M, Ghersi D, Liberati A, Petticrew M, Shekelle P, Stewart L, PRISMA-P Group. Preferred reporting items for systematic review and meta-analysis protocols (PRISMA-P) 2015: elaboration and explanation. BMJ. 2015 Jan 2;349(jan02 1):g7647.

Appendix Table 2: Definitions of pregnancy complications and risk factors

**Pregnancy Specific factors**	**Definitions**
Miscarriage/ Recurrent miscarriage/ Spontaneous pregnancy loss.	Spontaneous loss of a pregnancy before 24 weeks of gestation.<br>Early miscarriage, before 13 weeks of gestation.<br>Late miscarriage, between 13 and 24 weeks of gestation<br>Recurrent miscarriage is loss of three or more consecutive pregnancies before 24 weeks of gestation (36).
Stillbirth	child born after the 24th week of pregnancy who did not show any signs of life after being born(1).
Pre-eclampsia- early and late onset<br>Recurrent pre-eclampsia	New onset of hypertension (over 140 mmHg systolic or over 90 mmHg diastolic) after 20 weeks of pregnancy and the coexistence of 1 or more of the following new-onset conditions:<br>- proteinuria<br>- other maternal organ dysfunction (renal insufficiency, liver involvement, neurological complications, haematological complications)<br>- uteroplacental dysfunction(2).
Eclampsia<br>HELLP	The new onset of seizures or coma in a pregnant woman with pre-eclampsia<br>HELLP (Hemolysis, Elevated Liver enzymes and Low Platelets) syndrome is a life-threatening pregnancy complication usually considered to be a variant of pre-eclampsia(2).
Gestational hypertension	New hypertension presenting after 20 weeks of pregnancy without significant proteinuria(2).
Placenta previa	Placenta previa is low lying placenta after 20 weeks of pregnancy(3).

Placenta accreta	Placenta accrete is where placenta is stuck to the uterine muscle(3).
Placenta percreta	Placenta percreta is a condition where placenta attaches itself and grows through the uterus and potentially to the nearby organs (such as the bladder)(3).
Placental abruption	Placental abruption is the early separation of a placenta from the lining of the uterus before completion of the second stage of labor. It is one of the causes of bleeding during the second half of pregnancy. Placental abruption is also called abruptio placentae(4, 5).
Hyperemesis gravidarum	Severe, protracted nausea and vomiting associated with weight loss of more than 5% of pre pregnancy weight, dehydration and electrolyte imbalances(6).
Gestational diabetes mellitus	hyperglycaemia in pregnancy that is above diagnostic thresholds for diabetes<br>• a fasting plasma glucose level of 5.6 mmol/litre or above or<br>• a 2-hour plasma glucose level of 7.8 mmol/litre or above(7).
Ectopic pregnancy	If the fertilised egg fails to move from the fallopian tube into the uterus, where the pregnancy grows and develops and the fertilised egg may implant and start to develop outside the uterus(8).
Molar pregnancy/ Choriocarcinoma	Abnormal cells or tumours that start in the womb from cells that would normally develop into the placenta are called Gestational trophoblastic disease (GTD)<br>Gestational trophoblastic disease (GTD) forms a group of disorders spanning the conditions of complete and partial molar pregnancies through to the malignant conditions of invasive mole, choriocarcinoma(9, 10).
Multiple pregnancy/Twin-pregnancies/ Multiple gestation	Term used when one is expecting two or more babies at the same time (twins, triplets or more)(11).
Obstetric / postpartum haemorrhage (PPH)	Heavy bleeding after birth postpartum haemorrhage (PPH) can be primary or secondary. Primary PPH -blood loss 500 ml (a pint) or more of blood within the first 24 hours after the birth of the baby. Secondary PPH occurs when there is abnormal or heavy vaginal bleeding between 24 hours and 12 weeks after the birth(12).
Pre-term birth/ Recurrent pre-term birth	Babies born alive before 37 weeks of pregnancy are completed. extremely preterm (less than 28 weeks),very preterm (28 to 32 weeks),moderate to late preterm (32 to 37 weeks)(13).
Mode of birth: Instrumental	Birth of a singleton/multiple baby/babies between 37+0 and 42+6 weeks of gestation, who give birth vaginally with the assistance of instruments(ventouse and forceps births)(14).

Mode of birth Caesarean	Birth of a singleton/ multiple baby/babies baby between 37+0 and 42+6 weeks of gestation, by caesarean section(elective or emergency)(14).
Low birth weight	Weight at birth of < 2500 grams (5.5 pounds)(15).
Small for gestational age	Small for gestational age Term babies with a birth weight below the 10th centile, and below the 2nd centile are provided for reference(14).
Intra-uterine growth retardation Intra uterine growth restriction	Growth restriction implies a pathological restriction of the genetic growth potential. As a result, growth restricted fetuses may manifest evidence of fetal compromise (abnormal Doppler studies, reduced liquor volume). Small for gestational age Term babies with a birth weight below the 10th centile, and below the 2nd centile are provided for reference(14).
Fetal growth restriction	fetal growth restriction is defined as birth weight <3rd centile. We used severe small for gestational age at birth as a proxy for fetal growth restriction(16).
Post-partum depression	Depression like symptoms including low mood etc that starts within one or two months or several months of giving birth. Symptoms might last at least two weeks depending on the severity(17).
Puerperal psychosis	Puerperal psychosis is a severe mental illness that starts suddenly in the days, or weeks, after having a baby. Symptoms vary and can include high mood (mania), depression, confusion, hallucinations and delusions(18).
Perineal trauma-3rd and 4th degree tears	Severe perineal tears extending into the anal sphincter, anal mucosa are known as “third degree” and “fourth degree” tears also known as obstetric anal sphincter injury – OASI)(14, 19).
Obstetric cholestasis	Disorder that affects your liver during pregnancy causing build-up of bile acids in the body. The main symptom is itching of the skin and no rash. The symptoms get better when your baby has been born(20).
Pelvic girdle pain (PGP)	Pelvic girdle pain or symphysis pubis dysfunction (SPD).refers to pain in the front and/or the back of your pelvis .It also affect other areas such as the hips or thighs and the sacroiliac joints at the back and/or the symphysis pubis joint at the front(21).

Appendix Table 3. Search terms for pregnancy complications / risk factors (exposure)

Pregnancy complications/risk factors	pregnancy complication*.mp. or exp Pregnancy Complications/

Miscarriage/ Recurrent miscarriage/ Spontaneous pregnancy loss	exp Abortion, Spontaneous/ or ((recurrent adj3 miscarr$) or miscarr$ or early pregnancy loss$).mp.
Stillbirth	(stillbirth or still birth).mp. or exp Stillbirth/ or exp Fetal Death/ or (f?etal death$ or f?etal demise$).mp.
Hypertensive disorders of pregnancy /Gestational hypertension	Hypertension, Pregnancy-Induced/ or (gestational hypertension or (pregnancy adj3 hypertensi$)).mp.
Pre-eclampsia- early or late onset	(preeclampsia or pre-eclampsia).mp. or exp Pre-Eclampsia/
Eclampsia	exp Eclampsia/ or (eclampsia or tox?emia).mp.
HELLP (Homolysis, Elevated Liver enzymes and Low Platelets) syndrome	HELLP.mp. or exp HELLP Syndrome/
Placenta previa	placenta accreta.mp. or exp Placenta Accreta/ or placenta percreta.mp. or placenta increta.mp. or morbidly adherent placenta.mp. or abnormally invasive placenta.mp.
Placental abruption	Placenta$ abruption.mp. or exp Abruptio Placentae/
Placenta accrete and placenta percreta	placenta pr?eia.mp. or exp placenta previa/ or exp low lying placenta/
Hyperemesis gravidarum	Hyperemesis Gravidarum.mp. or Hyperemesis Gravidarum/ or morning sickness.mp. or exp Morning Sickness/
Gestational diabetes mellitus	(((pregnan$ or gestation$ or prenatal$ or antenatal$ or pre-natal$ or ante-natal$ or maternal$) adj2 diabet$) or gestational diabetes).mp. or exp Diabetes, Gestational/ or GDM.mp.
Ectopic pregnancy	ectopic pregnancy.mp. or exp Pregnancy, Ectopic/ or ((tub$$ adj3 pregnanc$) or (cornual adj3 pregnanc$) or (heterotopic adj3 pregnanc$) or (abdomin$ adj3 pregnanc$) or (extrauterine adj3 pregnanc$) or (interstitial adj3 pregnanc$) or (cervi$ adj3 pregnanc$) or (ovar$ adj3 pregnanc$) or (cesarean scar adj3 pregnanc$)).mp.
Molar pregnancy/ Choriocarcinoma	exp Gestational Trophoblastic Disease/ or gestational trophoblastic.mp. or exp Hydatidiform Mole/ or ((hydatid? adj2 mole?) or (molar adj2 pregnanc?)).mp.<br>exp Choriocarcinoma/ or choriocarcinoma.mp.

Multiple pregnancy/Twin-pregnancies/ Multiple gestation	exp Pregnancy, Multiple/ or ((pregnanc* or gestation*) adj (twin* or triplet* or quadruplet* or quintuplet* or multiple or multi?f?et*)).mp. or (Monochorionic or dichorionic).mp.
Obstetric haemorrhage (postpartum)	exp Postpartum Hemorrhage/ or (postpartum hemorrhage or post partum hemorrhage or postpartum haemorrhage or post partum haemorrhage).ti,ab. or obstetric haemorrhage.mp.
Pre-term birth/ Recurrent pre-term birth	obstetric labor, premature.mp. or exp Obstetric Labor, Premature/ or (premature labor or premature labor or preterm labor or preterm labor or preterm birth).mp.
Mode of delivery: Caesarean or Instrumental	exp Cesarean Section, Repeat/ or cesarean.mp. or exp Cesarean Section/ or (caesarean or cesarean or caesarian or cesarian or cesarien or caesarien or c-section or c section).mp.<br><br>exp Extraction, Obstetrical/ or exp Obstetrical Forceps/ or ((operative or instrumental or assisted or forcep* or ventouse* or vacuum*) adj1 (deliver* or birth*)).mp.
Low birth weight	low birth weight.mp. or exp Infant, Low Birth Weight/ or (low birth weight* adj4 very low birth weight*).mp.
Small for gestational age	exp Infant, Small for Gestational Age/ or small for gestational age.mp. or (small adj3 gestational age).mp.
Intra-uterine growth retardation / intra-uterine growth restriction<br><br>Fetal growth retardation	(intra?uterine growth adj2 (restriction* or retardation)).mp. or iugr.ti,ab<br>fetal growth retardation.mp. or exp Fetal Growth Retardation/ or (fetal growth adj2 (restriction? or retardation)).mp.
postpartum depression	postpartum depression.mp. or exp Depression, Postpartum/
Puerperal psychosis	((postpartum* or post partum* or post-partum* or postnatal* or post natal* or post-natal* or perinatal* or peri natal* or peri-natal* or puerp*) and (depress* or dysthymi* or adjustment disorder* or

	mood disorder* or affective disorder*)).mp.<br><br>(((postpartum* or post partum* or post-partum* or postnatal* or post natal* or post-natal or perinatal* or peri natal* or peri-natal* or puerp*) and (psychos#s or psychotic)) or psychosis after childbirth).mp.
Perineal trauma(3rd and 4th degree)	(((third or fourth or 3rd or 4th) adj degree) and tear*).mp.<br><br>(((anal near adj2 sphincter) or (rectal adj mucosa) or rectum or (anal adj epithelium) or anus or (recto?vaginal adj2 fistulae) or (anorectal adj mucosa) or (anal adj skin)) and (tear* or injur* or damage* or lacerat* or rupture* or trauma)).mp.<br><br>((obstetric* and anal and sphincter and injur*) or (anal and sphincter and injur*)).mp.
Obstetric cholestasis	(exp Pregnancy/ or exp Obstetrics/ or (pregnan* or obstetric*).mp.) and (exp Cholestasis/ or exp Cholestasis, Intrahepatic/)
Pelvic girdle pain(PGP)	pelvic girdle pain.mp. or exp Pelvic Girdle Pain/ or(symphysis pubis adj3 (pain$ or dysfunction$)).mp. or Pubic Symphysis Diastasis.mp. or exp Pubic Symphysis Diastasis/ or Sacroiliac joint dysfunction.mp. or PGP.mp.

<u>Appendix Table 4: Search terms for long-term health conditions (outcomes)</u>

**1-Autoimmune health conditions**	
**Health conditions**	**Search terms**
Autoimmune conditions	exp Autoimmune Diseases/ or (auto?immune adj2 (disease* or condition*)).mp.
Psoriasis	exp Psoriasis/ or psoria$.mp.
Vitiligo	vitiligo.mp. or exp Vitiligo/ or (leucoderma or leukoderma).mp.
Alopecia areata	alopecia areata.mp. or exp Alopecia Areata/ or (alopecia adj totalis).mp. or (alopecia adj universalis).mp.

Systemic lupus erythematosus	exp Lupus Erythematosus, Systemic/ or systemic lupus.mp. or sle.mp.
Psoriatic arthritis	exp Arthritis, Psoriatic/ or psoriatic.mp. or (psoria? adj (arthriti? or arthropath?)).mp. or ((arthriti? or arthropath?) adj psoria?).mp.
Ankylosing spondylitis	Ankylosing spondylitis.mp. or exp Spondylitis, Ankylosing/
Rheumatoid arthritis	exp Arthritis, Rheumatoid/ or rheumatoid artheritis.mp. or ((rheumatoid or reumatoid or revmatoid or rheumatic or reumatic or revmatic or rheumat$ or reumat$ or revmarthrit$) adj3 (arthrit$ or artrit$ or diseas$ or condition$ or nodule$)).mp.
Sjögren's's syndrome	exp Sjogren's Syndrome/ or sjogren.mp.
Systemic sclerosis	Systemic Sclerosis.mp. or exp Scleroderma, Systemic/ or ((systemic adj scler?) or sclero?).mp. or crest syndrome.mp. or exp CREST Syndrome/
Coeliac disease	celiac disease.mp. or exp Celiac Disease/ or (c?eliac adj disease*).mp.
Ulcerative colitis	ulcerative colitis.mp. or exp Colitis, Ulcerative/
Crohn's disease	Crohn's disease.mp. or exp Crohn Disease/
Inflammatory bowel disease	exp Inflammatory Bowel Diseases/ or inflammatory bowel.mp.
Multiple sclerosis	multiple sclerosis.mp. or exp Multiple Sclerosis/
Graves' disease	graves disease.mp. or exp Graves Disease/ or (grave? adj6 (diseas? or thyrotoxicos? or hyperthyr?)).mp.
Hashimoto's disease	exp Thyroiditis, Autoimmune/ or or autoimmune thyroid.mp. or Hashimoto Disease/
Type 1 diabetes	type 1 diabetes.mp. or exp Diabetes Mellitus, Type 1/ or ("typ? 1 diabet*" or "typ? I diabet*" or "typ?1 diabet*" or "typ?I diabet*").mp. or (("insulin* depend*" or "insulin?depend*") not ("non-insulin* depend*" or "non insulindepend*")).mp. or (IDDM or T1DM or T1D).mp. or autoimmune diabetes.mp.
Myasthenia gravis	exp Myasthenia Gravis/ or mysathenia.mp.
Addison's disease	exp Addison Disease/ or addison.mp.
**2-Cancers**	
Lung (C33-34)	exp Lung Neoplasms/ or lung cancer c33 c34.mp.
Breast (C50)	exp Breast Neoplasms/ or breast cancer.mp. or breast cancer c50.mp.
Colorectal	(colorectal or colon$ or rectal or rectum or sigmoid).ti,ab,sh.) And (cancer$ or neoplasm$ or

	or tumor or carcinoma or adenoma or adenocarcinoma).ti,ab,sh.)
Cervix	Uterine Cervical Neoplasms/ or cancer cervix.mp. or cervical cancer.mp. or cervical neoplasms.mps or cer$ cancer.mp.
Cancer of unknown primary (c77-c80)	Neoplasms, Unknown Primary/ or cancer of unknown primary.mp.
Pancreas (c25)	exp Pancreatic Neoplasms/ or cancer pancrea$.mp. or Carcinoma, Pancreatic Ductal or pancreas$ carcinoma.mp.
Ovary (c56- c57.4)	Exp Ovarian Neoplasms/ or ((ovarian or ovary or ovar$) adj2 carcinoma).tw. or ovary c56.mp.
Uterus (c54-c55)	Exp Uterine Neoplasms/ or Endometrial neoplasms/ or cancer, uter$.mp. or ((uterine or uterus or uter$) adj2 carcinoma).tw. or uterine c54.mp.
Oesophagus (c15)	exp Esophageal Neoplasms/ or ((esoph$ or oesoph$) adj3 (carcinoma or cancer)).tw.
Brain, Other CNS & Intracranial Tumours (C70-C72, C75.1-C75.3, D32-D33, D35.2-D35.4, D42-D43, D44.3-D44.5)	exp Central Nervous System Neoplasms/ or brain c70.mp. or brain d33.mp.
Liver (c22)	Exp Liver Neoplasms/ or liver cancer c22.mp.
Melanoma Skin Cancer (c43)	exp Skin neoplasms/ or exp Carcinoma, Basal cell/ or melanoma skin cancer.mp. or melanoma c43.mp. or BCC.mp.
Non-Hodgkin Lymphoma (c82-c86)	Exp Lymphoma, Non-Hodgkin/ or non-hodgkin lymphoma.mp.
Kidney (c64-66, c68)	Exp Kidney Neoplasms/ or kidney cancer.mp. or kidney c64.mp. or Carcinoma, Renal Cell/ or renal cancer.mp. or ((kidney or rena? or neph$) adj3 (carcinoma or cancer or neoplas?)).tw.
Thyroid	Exp Thyroid Neoplasms/ or Thyroid cancer.mp. or thyroid carcinoma.mp.
Leukemias	Leukemia/ or leukemia.mp. or leukaemia.mp.
**3-Functional health conditions**	
Chronic pain	exp Chronic Pain/
Fibromyalgia	fibromyalgia.mp. or exp FIBROMYALGIA/
Low back pain	exp Low Back Pain/ or exp Back Pain/ or chronic back pain.mp.
Interstitial cystitis	interstitial cystitis.mp. or exp Cystitis, Interstitial/
Irritable bowel syndrome	irritable bowel syndrome.mp. or exp Irritable Bowel Syndrome/

Singh M, et al. BMJ Open 2022; 12:e066476. doi: 10.1136/bmjopen-2022-066476

Vulvodynia	vulvodynia.mp. or exp VULVODYNIA/
Tension headache	tension headache.mp. or exp Tension -Type Headache/
**4-Mental health condition**	
Serious mental illness	((affective and non-affective) or (psychos*s or psychotic) or (bipolar* or bi-polar* or mania* or manic-depress**) or (schizophrenia* or schizo-affective or schizophrenia*) or (delusion* or paranoi*)).mp.
Common mental illness	(depress* or dysthymi* or mood disorder* or affective disorder* or distress* or neuros*).mp. or (anxiet* or obsessive compulsive disorder* or OCD* PTSD or post-traumatic stress disorder or panic* or neurosis* or neurotic* or adjustment disorder*).mp.
**5-Metabolic health conditions**	
Types 2 diabetes	exp Diabetes Mellitus, Type 2/ or diabetes type 2.mp. or Type 2/ or (MODY or NIDDM or T2DM or T2D).mp. or (non insulin* depend* or noninsulin* depend* or noninsulin?depend* or non insulin?depend*).mp. or ((typ? 2 or typ? II or typ?2 or typ?II) adj3 diabet*).mp. or (((late or adult* or matur* or slow or stabl*) adj3 onset) and diabet*).mp.
Hypertension	exp hypertension/ or hypertens$.mp. or exp blood pressure/ or (blood pressure or bloodpressure).mp.

Appendix Table 5.1 Search strategy- Medline database-Autoimmune condition and pregnancy complications

1	exp Autoimmune Diseases/
2	(auto?immune adj2 (disease* or condition*)).mp.
3	exp Psoriasis/
4	psoria$.mp.
5	exp Vitiligo/
6	vitiligo.mp.
7	(leucoderma or leukoderma).mp.
8	exp Alopecia Areata/
9	alopecia areata.mp.
10	((alopecia adj totalis) or (alopecia adj universalis)).mp.
11	exp Lupus Erythematosus, Systemic/
12	(systemic lupus or sle).mp.
13	arthritis psoriatic.mp. or exp Arthritis, Psoriatic/

14	Ankylosing spondylitis.mp. or exp Spondylitis, Ankylosing/
15	Spondylarthritis/
16	(axial adj2 spondylarthritis).tw.
17	(axial adj2 spa).tw.
18	(ankylos$ or spondyl$).tw.
19	(bekhterev$ or bechterew$).tw.
20	(Marie adj struempell$).tw.
21	(AS or axSPA).tw.
22	Sacroiliitis/
23	((axial or spin$ or peripheral or vertebral or enthesitis) adj3 (joint$ or spondyloarthritis or arthritis or ankylosing)).tw.
24	exp Arthritis, Rheumatoid/
25	((rheumatoid or reumatoid or revmatoid or rheumatic or reumatic or revmatic or rheumat$ or reumat$ or revmarthrit$) adj3 (arthrit$ or artrit$ or diseas$ or condition$ or nodule$)).mp.
26	sjogren syndrome.mp. or exp Sjogren's Syndrome/
27	exp Celiac Disease/
28	(c?eliac adj disease*).mp.
29	inflammatory bowel disease.mp. or exp Inflammatory Bowel Diseases/
30	ulcerative colitis.mp. or exp Colitis, Ulcerative/
31	Crohn's disease.mp. or exp Crohn Disease/
32	multiple sclerosis.mp. or exp Multiple Sclerosis/
33	Systemic Sclerosis.mp.
34	exp Scleroderma, Systemic/
35	((systemic adj scler?) or sclero?).mp.
36	crest syndrome.mp. or exp CREST Syndrome/
37	graves disease.mp. or exp Graves Disease/
38	(grave? adj3 (diseas? or thyrotoxicos? or hyperthyr?)).mp.
39	exp Thyroiditis, Autoimmune/
40	autoimmune thyroid.mp.
41	Hashimoto Disease.mp.
42	exp Hashimoto Disease/
43	(thyroid adj autoantibodies).mp. [mp=title, book title, abstract, original title, name of substance word, subject heading word, floating sub-heading word, keyword heading word, organism supplementary concept word, protocol supplementary concept word, rare disease supplementary concept word, unique identifier, synonyms]
44	thyroid.mp. and exp autoantibodies/
45	myasthenia gravis.mp. or exp Myasthenia Gravis/
46	addison disease.mp. or exp Addison Disease/
47	type 1 diabetes.mp. or exp Diabetes Mellitus, Type 1/ or ("typ? 1 diabet*" or "typ? I diabet*" or "typ?1 diabet*" or "typ?I diabet*").mp. or (("insulin* depend*" or "insulin?depend*") not ("non-insulin* depend*" or "non insulindepend*")).mp. or (IDDM or T1DM or T1D).mp. or autoimmune diabetes.mp.
48	exp Pregnancy Complications/
49	(pregnancy adj complication?).mp.
50	exp Abortion, Spontaneous/
51	((recurrent adj2 miscarr?) or miscarr?).mp.
52	(early adj3 pregnancy loss?).mp.
53	miscarriage.mp.
54	exp Stillbirth/

55	(stillbirth or (still adj birth)).mp.
56	exp Fetal Death/
57	((f?etal adj death$) or (f?etal adj demise$)).mp.
58	exp Hypertension, Pregnancy-Induced/
59	(gestational hypertension or (pregnancy adj3 hypertensi$)).mp.
60	(preeclampsia or pre-eclampsia).mp.
61	exp Pre-Eclampsia/
62	(eclampsia or tox?emia).mp.
63	exp Eclampsia/
64	hellp.mp. or exp HELLP Syndrome/
65	exp Placenta Accreta/
66	(placenta adj accreta).mp.
67	placenta percreta.mp.
68	placenta increta.mp.
69	(morbidly adj3 adherent placenta).mp.
70	abnormally invasive placenta.mp.
71	exp Abruptio Placentae/
72	Placenta? abruption.mp.
73	exp Placenta Previa/
74	Placenta pr?evia.mp.
75	low lying placenta.mp.
76	exp Hyperemesis Gravidarum/
77	Hyperemesis Gravidarum.mp.
78	exp Morning Sickness/
79	morning sickness.mp.
80	exp Diabetes, Gestational/
81	GDM.mp.
82	(((pregnan$ or gestation$ or prenatal$ or antenatal$ or pre-natal$ or ante-natal$ or maternal$) adj2 diabet$) or gestational diabetes).mp.
83	((tub$$ adj3 pregnanc$) or (cornual adj3 pregnanc$) or (heterotopic adj3 pregnanc$) or (abdomin$ adj3 pregnanc$) or (extrauterine adj3 pregnanc$) or (interstitial adj3 pregnanc$) or (cervi$ adj3 pregnanc$) or (ovar$ adj3 pregnanc$) or (cesarean scar adj3 pregnanc$)).mp.
84	exp Pregnancy, Ectopic/
85	ectopic pregnancy.mp.
86	exp Gestational Trophoblastic Disease/
87	gestational trophoblastic.mp.
88	exp Hydatidiform Mole/
89	((hydatid? adj2 mole?) or (molar adj2 pregnanc?)).mp.
90	exp Choriocarcinoma/
91	choriocarcinoma.mp.
92	exp Pregnancy, Multiple/
93	((pregnanc* or gestation*) adj (twin* or triplet* or quadruplet* or quintuplet* or multiple or multi?f?et*)).mp.
94	(Monochorionic or dichorionic).mp.
95	exp Postpartum Hemorrhage/
96	(postpartum hemorrhage or post partum hemorrhage or postpartum haemorrhage or post partum haemorrhage).mp.
97	obstetric haemorrhage.mp.
98	obstetric labor, premature.mp.

99	exp Obstetric Labor, Premature/
100	(premature labor or premature labour or preterm labor or preterm labour or preterm birth).mp.
101	exp Cesarean Section/
102	exp Cesarean Section, Repeat/
103	(caesarean or cesarean or caesarian or cesarian or cesarien or caesarien or c-section or c section).mp.
104	cesarean.mp.
105	exp Extraction, Obstetrical/
106	exp Obstetrical Forceps/
107	((operative or instrumental or assisted or forcep* or ventouse* or vacuum*) adj1 (deliver* or birth*)).mp.
108	exp Infant, Low Birth Weight/
109	low birth weight.mp.
110	(low birth weight* adj4 very low birth weight*).mp.
111	exp Infant, Small for Gestational Age/
112	(small adj3 gestational age).mp.
113	(intra?uterine growth adj2 (restriction* or retardation)).mp.
114	iugr.ti,ab.
115	exp Fetal Growth Retardation/
116	(fetal growth adj2 (restriction? or retardation)).mp.
117	exp Depression, Postpartum/
118	postpartum depression.mp.
119	((postpartum* or post partum* or post-partum* or postnatal* or post natal* or post-natal* or perinatal* or peri natal* or peri-natal* or puerp*) and (depress* or dysthymi* or adjustment disorder* or mood disorder* or affective disorder*)).mp.
120	((postpartum* or post partum* or post-partum* or postnatal* or post natal* or post-natal or perinatal* or peri natal* or peri-natal* or puerp*) and (psychos#s or psychotic)).mp.
121	(psychosis adj3 after childbirth).mp.
122	(((third or fourth or 3rd or 4th) adj degree) and tear*).mp.
123	(((anal near adj2 sphincter) or (rectal adj mucosa) or rectum or (anal adj epithelium) or anus or (recto?vaginal adj2 fistulae) or (anorectal adj mucosa) or (anal adj skin)) and (tear* or injur* or damage* or lacerat* or rupture* or trauma)).mp.
124	((obstetric* and anal and sphincter and injur*) or (anal and sphincter and injur*)).mp.
125	(exp Pregnancy/ or exp Obstetrics/ or (pregnan* or obstetric*).mp.) and (exp Cholestasis/ or exp Cholestasis, Intrahepatic/)
126	pelvic girdle pain.mp. or exp Pelvic Girdle Pain/
127	(symphysis pubis adj3 (pain$ or dysfunction$)).mp.
128	Pubic Symphysis Diastasis.mp. or exp Pubic Symphysis Diastasis/
129	Sacroiliac joint dysfunction.mp.
130	PGP.mp.
131	1 or 2 or 3 or 4 or 5 or 6 or 7 or 8 or 9 or 10 or 11 or 12 or 13 or 14 or 15 or 16 or 17 or 18 or 19 or 20 or 21 or 22 or 23 or 24 or 25 or 26 or 27 or 28 or 29 or 30 or 31 or 32 or 33 or 34 or 35 or 36 or 37 or 39 or 40 or 41 or 42 or 43 or 44 or 45 or 46 or 47
132	48 or 49 or 50 or 51 or 52 or 53 or 54 or 55 or 56 or 57 or 58 or 59 or 60 or 61 or 62 or 63 or 64 or 65 or 66 or 67 or 68 or 69 or 70 or 71 or 72 or 73 or 74 or 75 or 76 or 77 or 78 or 79 or 80 or 81 or 82 or 83 or 84 or 85 or 86 or 87 or 88 or 89 or 90 or 91 or 92 or 93 or 94 or 95 or 96 or 97 or 98 or 99 or 100 or 101 or 102 or 103 or 104 or 105 or 106 or 107 or 108 or 109 or 110 or 111 or 112 or 113 or 114 or 115 or 116 or 117 or 118 or 119 or 120 or 121 or 122 or 123 or 124 or 125 or 126 or 127 or 128 or 129 or 130

133	131 and 132
134	(systematic$ adj2 (review$ or overview)).ti,ab.
135	(systematic$ adj5 review$).tw,sh.
136	meta-analysis.mp. or exp meta-analysis/
137	134 or 135 or 136
138	133 and 137

Appendix Table 5.2 search strategy- Medline database- Cancer and pregnancy complications

1.	exp Abortion, Spontaneous/
2.	(stillbirth or still birth).mp.
3.	(recurrent adj3 miscarr$).mp. [mp=title, abstract, original title, name of substance word, subject heading word, floating sub-heading word, keyword heading word, organism supplementary concept word, protocol supplementary concept word, rare disease supplementary concept word, unique identifier, synonyms]
4.	(miscarr$ or early pregnancy loss$).mp. [mp=title, abstract, original title, name of substance word, subject heading word, floating sub-heading word, keyword heading word, organism supplementary concept word, protocol supplementary concept word, rare disease supplementary concept word, unique identifier, synonyms]
5.	exp Stillbirth/
6.	exp Fetal Death/
7.	(f?etal death$ or f?etal demise$).mp.
8.	Hypertension, Pregnancy-Induced/
9.	(gestational hypertension or (pregnancy adj3 hypertensi$)).mp.
10.	(preeclampsia or pre-eclampsia).mp.
11.	exp Pre-Eclampsia/
12.	exp Eclampsia/
13.	(eclampsia or tox?emia).mp.
14.	HELLP.mp.
15.	exp HELLP Syndrome/
16.	placenta accreta.mp.
17.	exp Placenta Accreta/
18.	placenta percreta.mp.
19.	placenta increta.mp.
20.	morbidly adherent placenta.mp.
21.	abnormally invasive placenta.mp.
22.	Placenta$ abruption.mp.
23.	exp Abruptio Placentae/
24.	placenta pr?eia.mp.
25.	exp placenta previa/
26.	low lying placenta.mp.
27.	Hyperemesis Gravidarum.mp.
28.	Hyperemesis Gravidarum/

29.	morning sickness.mp.
30.	exp Morning Sickness/
31.	(((pregnan$ or gestation$ or prenatal$ or antenatal$ or pre-natal$ or ante-natal$ or maternal$) adj2 diabet$) or gestational diabetes).mp.
32.	exp Diabetes, Gestational/
33.	GDM.mp.
34.	ectopic pregnancy.mp.
35.	exp Pregnancy, Ectopic/
36.	((tub$$ adj3 pregnanc$) or (cornual adj3 pregnanc$) or (heterotopic adj3 pregnanc$) or (abdomin$ adj3 pregnanc$) or (extrauterine adj3 pregnanc$) or (interstitial adj3 pregnanc$) or (cervi$ adj3 pregnanc$) or (ovar$ adj3 pregnanc$) or (cesarean scar adj3 pregnanc$)).mp.
37.	exp Gestational Trophoblastic Disease/
38.	gestational trophoblastic.mp.
39.	exp Hydatidiform Mole/
40.	((hydatid? adj2 mole?) or (molar adj2 pregnanc?)).mp.
41.	exp Choriocarcinoma/ or choriocarcinoma.mp.
42.	exp Pregnancy, Multiple/
43.	exp Pregnancy, Multiple/ or ((pregnanc* or gestation*) adj (twin* or triplet* or quadruplet* or quintuplet* or multiple or multi?f?et*)).mp.
44.	(Monochorionic or dichorionic).mp.
45.	exp Postpartum Hemorrhage/
46.	(postpartum hemorrhage or post partum hemorrhage or postpartum haemorrhage or post partum haemorrhage).ti,ab.
47.	obstetric haemorrhage.mp.
48.	obstetric labor, premature.mp.
49.	exp Obstetric Labor, Premature/
50.	(premature labor or premature labour or preterm labor or preterm labour or preterm birth).mp.
51.	exp Cesarean Section, Repeat/ or cesarean.mp.
52.	exp Cesarean Section/
53.	(caesarean or cesarean or caesarian or cesarian or cesarien or caesarien or c-section or c section).mp.
54.	exp Extraction, Obstetrical/
55.	exp Obstetrical Forceps/
56.	((operative or instrumental or assisted or forcep* or ventouse* or vacuum*) adj1 (deliver* or birth*)).mp.
57.	low birth weight.mp.
58.	exp Infant, Low Birth Weight/
59.	(low birth weight* adj4 very low birth weight*).mp.
60.	exp Infant, Small for Gestational Age/
61.	small for gestational age.mp.
62.	(small adj3 gestational age).mp.
63.	(intra?uterine growth adj2 (restriction* or retardation)).mp.
64.	iugr.ti,ab.
65.	fetal growth retardation.mp.

66.	exp Fetal Growth Retardation/
67.	foetal growth retardation.mp.
68.	(fetal growth adj2 (restriction? or retardation)).mp.
69.	(foetal growth adj2 (restriction? or retardation)).mp.
70.	((postpartum* or post partum* or post-partum* or postnatal* or post natal* or post-natal* or perinatal* or peri natal* or peri-natal* or puerp*) and (depress* or dysthymi* or adjustment disorder* or mood disorder* or affective disorder*)).mp.
71.	(((third or fourth or 3rd or 4th) adj degree) and tear*).mp.
72.	(((anal near adj2 sphincter) or (rectal adj mucosa) or rectum or (anal adj epithelium) or anus or (recto?vaginal adj2 fistulae) or (anorectal adj mucosa) or (anal adj skin)) and (tear* or injur* or damage* or lacerat* or rupture* or trauma)).mp.
73.	((obstetric* and anal and sphincter and injur*) or (anal and sphincter and injur*)).mp.
74.	(exp Pregnancy/ or exp Obstetrics/ or (pregnan* or obstetric*).mp.) and (exp Cholestasis/ or exp Cholestasis, Intrahepatic/)
75	pregnancy complication*.mp.
76	exp Pregnancy Complications/
77	exp Lung Neoplasms/
78.	lung cancer c33 c34.mp.
79	exp Breast Neoplasms/
80	breast cancer.mp.
81	breast cancer c50.mp.
82	Uterine Cervical Neoplasms/
83	cancer cervix.mp.
84	cervical cancer.mp.
85	cervical neoplasms.mp.
86	cer$ cancer.mp.
87	Neoplasms, Unknown Primary/
88.	cancer of unknown primary.mp.
89	exp Pancreatic Neoplasms/
90	cancer pancrea$.mp.
91	Carcinoma, Pancreatic Ductal/
92	pancreas$ carcinoma.mp.
93	exp Ovarian Neoplasms/
94	((ovarian or ovary or ovar$) adj2 carcinoma).tw.
95	ovary c56.mp.
96	exp Uterine Neoplasms/
97	Endometrial neoplasms/
98	cancer, uter$.mp.
99	((uterine or uterus or uter$) adj2 carcinoma).tw.
100	uterine c54.mp.
101	exp Esophageal Neoplasms/
102	((esoph$ or oesoph$) adj3 (carcinoma or cancer)).tw.
103	exp Central Nervous System Neoplasms/
104	brain c70.mp.

105	brain d33.mp.
106	exp Liver Neoplasms/
107	liver cancer c22.mp.
108	exp Skin neoplasms/
109	melanoma skin cancer.mp.
110	melanoma c43.mp.
111	exp Kidney Neoplasms/
112	kidney cancer.mp.
113	kidney c64.mp.
114	Carcinoma, Renal Cell/
115	renal cancer.mp.
116	((kidney or rena? or neph$) adj3 (carcinoma or cancer or neoplas?)).tw.
117	((kidney or rena? or neph$) adj3 (carcinoma or cancer or neoplas?)).tw.
118	exp Thyroid Neoplasms/
119	Thyroid cancer.mp.
120	thyroid carcinoma.mp.
121	Leukemia/
122	leukemia.mp.
123	leukaemia.mp.
124	exp Colorectal Neoplasms/
125	colorectal cancer.mp.
126.	exp Lymphoma/
127	lymphoma.mp.
128	1 or 2 or 3 or 4 or 5 or 6 or 7or 8 or 9 or 10 or 11 or 12 or 13 or 14 or 15 or 16 or 17 or 18 or 19 or 20 or 21 or 22 or 23 or 24 or 25 or 26 or 27 or 28 or 29 or 30 or 31 or 32 or 33 or 34 or 35 or 36 or 37 or 38 or 39 or 40 or 41 or 42 or 43 or 44 or 45 or 46 or 47 or 48 or 49 or 50 or 51 or 52 or 53 or 54 or 55 or 56 or 57 or 58 or 59 or 60 or 61 or 62 or 63 or 64 or 65 or 66 or 67 or 68 or 69 or 71 or 72 or 73 or 74 or 75 or 76
129	77 or 78 or 79 or 80 or 81 or 82 or 83 or 84 or 85 or 86 or 87 or 88 or 89 or 90 or 91 or 92 or 93 or 94 or 95 or 96 or 97 or 98 or 99 or 100 or 101 or 102 or 103 or 104 or 105 or 106 or 107 or 108 or 109 or 110 or 111 or 112 or 113 or 114 or 115 or 116 or 117 or 118 or 119 or 120 0r 121 or 122 or 123 or 124 or 125 or 126 or 127
130	128 or 129
131	(systematic$ adj2 (review$ or overview)).ti,ab.
132	(systematic$ adj5 review$).tw,sh.
133	meta-analysis.mp. or exp meta-analysis/
134	131 or 132 or 133
135	130 and 134

Appendix Table 5.3 Search strategy- Medline database- Funtional conditions and pregnancy complications

1.	exp Abortion, Spontaneous/
2.	(stillbirth or still birth).mp.
3.	(recurrent adj3 miscarr$).mp. [mp=title, abstract, original title, name of substance word, subject heading word, floating sub-heading word, keyword heading word, organism supplementary concept word, protocol supplementary concept word, rare disease supplementary concept word, unique identifier, synonyms]
4.	(miscarr$ or early pregnancy loss$).mp. [mp=title, abstract, original title, name of substance word, subject heading word, floating sub-heading word, keyword heading word, organism supplementary concept word, protocol supplementary concept word, rare disease supplementary concept word, unique identifier, synonyms]
5.	exp Stillbirth/
6.	exp Fetal Death/
7.	(f?etal death$ or f?etal demise$).mp.
8.	Hypertension, Pregnancy-Induced/
9.	(gestational hypertension or (pregnancy adj3 hypertensi$)).mp.
10.	(preeclampsia or pre-eclampsia).mp.
11.	exp Pre-Eclampsia/
12.	exp Eclampsia/
13.	(eclampsia or tox?emia).mp.
14.	HELLP.mp.
15.	exp HELLP Syndrome/
16.	placenta accreta.mp.
17.	exp Placenta Accreta/
18.	placenta percreta.mp.
19.	placenta increta.mp.
20.	morbidly adherent placenta.mp.
21.	abnormally invasive placenta.mp.
22.	Placenta$ abruption.mp.
23.	exp Abruptio Placentae/
24.	placenta pr?eia.mp.
25.	exp placenta previa/
26.	low lying placenta.mp.
27.	Hyperemesis Gravidarum.mp.
28.	Hyperemesis Gravidarum/
29.	morning sickness.mp.
30.	exp Morning Sickness/
31.	(((pregnan$ or gestation$ or prenatal$ or antenatal$ or pre-natal$ or ante-natal$ or maternal$) adj2 diabet$) or gestational diabetes).mp.
32.	exp Diabetes, Gestational/
33.	GDM.mp.
34.	ectopic pregnancy.mp.
35.	exp Pregnancy, Ectopic/

36.	((tub$$ adj3 pregnanc$) or (cornual adj3 pregnanc$) or (heterotopic adj3 pregnanc$) or (abdomin$ adj3 pregnanc$) or (extrauterine adj3 pregnanc$) or (interstitial adj3 pregnanc$) or (cervi$ adj3 pregnanc$) or (ovar$ adj3 pregnanc$) or (cesarean scar adj3 pregnanc$)).mp.
37.	exp Gestational Trophoblastic Disease/
38.	gestational trophoblastic.mp.
39.	exp Hydatidiform Mole/
40.	((hydatid? adj2 mole?) or (molar adj2 pregnanc?)).mp.
41.	exp Choriocarcinoma/ or choriocarcinoma.mp.
42.	exp Pregnancy, Multiple/
43.	exp Pregnancy, Multiple/ or ((pregnanc* or gestation*) adj (twin* or triplet* or quadruplet* or quintuplet* or multiple or multi?f?et*)).mp.
44.	(Monochorionic or dichorionic).mp.
45.	exp Postpartum Hemorrhage/
46.	(postpartum hemorrhage or post partum hemorrhage or postpartum haemorrhage or post partum haemorrhage).ti,ab.
47.	obstetric haemorrhage.mp.
48.	obstetric labor, premature.mp.
49.	exp Obstetric Labor, Premature/
50.	(premature labor or premature labour or preterm labor or preterm labour or preterm birth).mp.
51.	exp Cesarean Section, Repeat/ or cesarean.mp.
52.	exp Cesarean Section/
53.	(caesarean or cesarean or caesarian or cesarian or cesarien or caesarien or c-section or c section).mp.
54.	exp Extraction, Obstetrical/
55.	exp Obstetrical Forceps/
56.	((operative or instrumental or assisted or forcep* or ventouse* or vacuum*) adj1 (deliver* or birth*)).mp.
57.	low birth weight.mp.
58.	exp Infant, Low Birth Weight/
59.	(low birth weight* adj4 very low birth weight*).mp.
60.	exp Infant, Small for Gestational Age/
61.	small for gestational age.mp.
62.	(small adj3 gestational age).mp.
63.	(intra?uterine growth adj2 (restriction* or retardation)).mp.
64.	iugr.ti,ab.
65.	fetal growth retardation.mp.
66.	exp Fetal Growth Retardation/
67.	foetal growth retardation.mp.
68.	(fetal growth adj2 (restriction? or retardation)).mp.
69.	(foetal growth adj2 (restriction? or retardation)).mp.
70.	((postpartum* or post partum* or post-partum* or postnatal* or post natal* or post-natal* or perinatal* or peri natal* or peri-natal* or puerp*) and (depress* or dysthymi* or adjustment disorder* or mood disorder* or affective disorder*)).mp.
71.	(((third or fourth or 3rd or 4th) adj degree) and tear*).mp.

72.	(((anal near adj2 sphincter) or (rectal adj mucosa) or rectum or (anal adj epithelium) or anus or (recto?vaginal adj2 fistulae) or (anorectal adj mucosa) or (anal adj skin)) and (tear* or injur* or damage* or lacerat* or rupture* or trauma)).mp.
73.	((obstetric* and anal and sphincter and injur*) or (anal and sphincter and injur*)).mp.
74.	(exp Pregnancy/ or exp Obstetrics/ or (pregnan* or obstetric*).mp.) and (exp Cholestasis/ or exp Cholestasis, Intrahepatic/)
75	pregnancy complication*.mp.
76	exp Pregnancy Complications/
77	exp FIBROMYALGIA/
78.	exp Low Back Pain/
79	exp Back Pain/
80	chronic back pain.mp.
81	interstitial cystitis.mp.
82	exp Cystitis, Interstitial/
83	irritable bowel syndrome.mp.
84	exp Irritable Bowel Syndrome/
85	vulvodynia.mp.
86	exp VULVODYNIA/
87	tension headache.mp.
88.	exp Tension -Type Headache/
89	1 or 2 or 3 or 4 or 5 or 6 or 7or 8 or 9 or 10 or 11 or 12 or 13 or 14 or 15 or 16 or 17 or 18 or 19 or 20 or 21 or 22 or 23 or 24 or 25 or 26 or 27 or 28 or 29 or 30 or 31 or 32 or 33 or 34 or 35 or 36 or 37 or 38 or 39 or 40 or 41 or 42 or 43 or 44 or 45 or 46 or 47 or 48 or 49 or 50 or 51 or 52 or 53 or 54 or 55 or 56 or 57 or 58 or 59 or 60 or 61 or 62 or 63 or 64 or 65 or 66 or 67 or 68 or 69 or 71 or 72 or 73 or 74 or 75 or 76
90	77 or 78 or 79 or 80 or 81 or 82 or 83 or 84 or 85 or 86 or 87 or 88
91	89 and 90
92	(systematic$ adj2 (review$ or overview)).ti,ab.
93	(systematic$ adj5 review$).tw,sh.
94	meta-analysis.mp. or exp meta-analysis/
95	92 or 93 or 94
96	91 and 95

Appendix Table 5.4 search strategy-mental health conditions and pregnancy complications

	Search term
1	pregnancy complication*.mp. or exp Pregnancy Complications/
2	exp Abortion, Spontaneous/ or ((recurrent adj3 miscarr$) or miscarr$ or early pregnancy loss$).mp.
3	(stillbirth or still birth).mp. or exp Stillbirth/ or exp Fetal Death/ or (f?etal death$ or f?etal demise$).mp.
4	Hypertension, Pregnancy-Induced/ or (gestational hypertension or (pregnancy adj3 hypertensi$)).mp.
5	(preeclampsia or pre-eclampsia).mp. or exp Pre-Eclampsia/
6	exp Eclampsia/ or (eclampsia or tox?emia).mp.
7	HELLP.mp. or exp HELLP Syndrome/
8	placenta accreta.mp. or exp Placenta Accreta/ or placenta percreta.mp. or placenta increta.mp. or morbidly adherent placenta.mp. or abnormally invasive placenta.mp.
9	Placenta$ abruption.mp. or exp Abruptio Placentae/
10	placenta pr?eia.mp. or exp placenta previa/ or exp low lying placenta/
11	Hyperemesis Gravidarum.mp. or Hyperemesis Gravidarum/ or morning sickness.mp. or exp Morning Sickness/
12	(((pregnan$ or gestation$ or prenatal$ or antenatal$ or pre-natal$ or ante-natal$ or maternal$) adj2 diabet$) or gestational diabetes).mp. or exp Diabetes, Gestational/ or GDM.mp.
13	ectopic pregnancy.mp. or exp Pregnancy, Ectopic/ or ((tub$$ adj3 pregnanc$) or (cornual adj3 pregnanc$) or (heterotopic adj3 pregnanc$) or (abdomin$ adj3 pregnanc$)

	or (extrauterine adj3 pregnanc$) or (interstitial adj3 pregnanc$) or (cervi$ adj3 pregnanc$) or (ovar$ adj3 pregnanc$) or (cesarean scar adj3 pregnanc$)).mp.
14	exp Gestational Trophoblastic Disease/ or gestational trophoblastic.mp. or exp Hydatidiform Mole/ or ((hydatid? adj2 mole?) or (molar adj2 pregnanc?)).mp. or exp Choriocarcinoma/ or choriocarcinoma.mp.
15	exp Pregnancy, Multiple/ or ((pregnanc* or gestation*) adj (twin* or triplet* or quadruplet* or quintuplet* or multiple or multi?f?et*)).mp. or (Monochorionic or dichorionic).mp.
16	exp Postpartum Hemorrhage/ or (postpartum hemorrhage or post partum hemorrhage or postpartum haemorrhage or post partum haemorrhage).ti,ab. or obstetric haemorrhage.mp.
17	obstetric labor, premature.mp. or exp Obstetric Labor, Premature/ or (premature labor or premature labour or preterm labor or preterm labour or preterm birth).mp.
18	exp Cesarean Section, Repeat/ or cesarean.mp. or exp Cesarean Section/ or (caesarean or cesarean or caesarian or cesarian or cesarien or caesarien or c-section or c section).mp. or exp Extraction, Obstetrical/ or exp Obstetrical Forceps/ or ((operative or instrumental or assisted or forcep* or ventouse* or vacuum*) adj1 (deliver* or birth*)).mp.
19	low birth weight.mp. or exp Infant, Low Birth Weight/ or (low birth weight* adj4 very low birth weight*).mp.
20	exp Infant, Small for Gestational Age/ or small for gestational age.mp. or (small adj3 gestational age).mp.
21	(intra?uterine growth adj2 (restriction* or retardation)).mp. or iugr.ti,ab. or fetal growth retardation.mp. or exp Fetal Growth Retardation/ or (fetal growth adj2 (restriction? or retardation)).mp.
22	((postpartum* or post partum* or post-partum* or postnatal* or post natal* or post-natal* or perinatal* or peri natal* or peri-natal* or puerp*) and (depress* or dysthymi* or adjustment disorder* or mood disorder* or affective disorder*)).mp.

Singh M, *et al. BMJ Open* 2022; 12:e066476. doi: 10.1136/bmjopen-2022-066476

23	(((postpartum* or post partum* or post-partum* or postnatal* or post natal* or post-natal or perinatal* or peri natal* or peri-natal* or puerp*) and (psychos#s or psychotic)) or psychosis after childbirth).mp.
24	((((third or fourth or 3rd or 4th) adj degree) and tear*) or (((anal near adj2 sphincter) or (rectal adj mucosa) or rectum or (anal adj epithelium) or anus or (recto?vaginal adj2 fistulae) or (anorectal adj mucosa) or (anal adj skin)) and (tear* or injur* or damage* or lacerat* or rupture* or trauma)) or ((obstetric* and anal and sphincter and injur*) or (anal and sphincter and injur*))).mp.
25	(exp Pregnancy/ or exp Obstetrics/ or (pregnan* or obstetric*).mp.) and (exp Cholestasis/ or exp Cholestasis, Intrahepatic/)
26	pelvic girdle pain.mp. or exp Pelvic Girdle Pain/ or (symphysis pubis adj3 (pain$ or dysfunction$)).mp. or Pubic Symphysis Diastasis.mp. or exp Pubic Symphysis Diastasis/ or Sacroiliac joint dysfunction.mp. or PGP.mp.
27	1 or 2 or 3 or 4 or 5 or 6 or 7 or 8 or 9 or 10 or 11 or 12 or 13 or 14 or 15 or 16 or 17 or 18 or 19 or 20 or 21 or 22 or 23 or 24 or 25 or 26
28	((affective and non-affective) or (psychos*s or psychotic) or (bipolar* or bi-polar* or mania* or manic-depress**) or (schizophrenia* or schizo-affective or schizophrenia*) or (delusion* or paranoi*)).mp.
29	(depress* or dysthymi* or mood disorder* or affective disorder* or distress* or neuros*).mp.
30	(anxiet* or obsessive compulsive disorder* or OCD* PTSD or post-traumatic stress disorder or panic* or neurosis* or neurotic* or adjustment disorder*).mp.
31	(systematic review or meta-analysis).mp.
32	29 or 30
33	27 and 28

34	27 and 28 and 31
35	27 and 32
36	27 and 31 and 32

Appendix Table 5.5 Search strategy- Medline database-metabolic conditions and pregnancy complications

	**Search terms**
1	pregnancy complication*.mp. or exp Pregnancy Complications/
2	exp Abortion, Spontaneous/ or ((recurrent adj3 miscarr$) or miscarr$ or early pregnancy loss$).mp.
3	(stillbirth or still birth).mp. or exp Stillbirth/ or exp Fetal Death/ or (f?etal death$ or f?etal demise$).mp.
4	Hypertension, Pregnancy-Induced/ or (gestational hypertension or (pregnancy adj3 hypertensi$)).mp.
5	(preeclampsia or pre-eclampsia).mp. or exp Pre-Eclampsia/
6	exp Eclampsia/ or (eclampsia or tox?emia).mp.
7	HELLP.mp. or exp HELLP Syndrome/
8	placenta accreta.mp. or exp Placenta Accreta/ or placenta percreta.mp. or placenta increta.mp. or morbidly adherent placenta.mp. or abnormally invasive placenta.mp.
9	Placenta$ abruption.mp. or exp Abruptio Placentae/
10	placenta pr?eia.mp. or exp placenta previa/ or exp low lying placenta/
11	Hyperemesis Gravidarum.mp. or Hyperemesis Gravidarum/ or morning sickness.mp. or exp Morning Sickness/

12	(((pregnan$ or gestation$ or prenatal$ or antenatal$ or pre-natal$ or ante-natal$ or maternal$) adj2 diabet$) or gestational diabetes).mp. or exp Diabetes, Gestational/ or GDM.mp.
13	ectopic pregnancy.mp. or exp Pregnancy, Ectopic/ or ((tub$$ adj3 pregnanc$) or (cornual adj3 pregnanc$) or (heterotopic adj3 pregnanc$) or (abdomin$ adj3 pregnanc$) or (extrauterine adj3 pregnanc$) or (interstitial adj3 pregnanc$) or (cervi$ adj3 pregnanc$) or (ovar$ adj3 pregnanc$) or (cesarean scar adj3 pregnanc$)).mp.
14	exp Gestational Trophoblastic Disease/ or gestational trophoblastic.mp. or exp Hydatidiform Mole/ or ((hydatid? adj2 mole?) or (molar adj2 pregnanc?)).mp.
15	exp Choriocarcinoma/ or choriocarcinoma.mp.
16	exp Pregnancy, Multiple/ or ((pregnanc* or gestation*) adj (twin* or triplet* or quadruplet* or quintuplet* or multiple or multi?f?et*)).mp. or (Monochorionic or dichorionic).mp.
17	exp Postpartum Hemorrhage/ or (postpartum hemorrhage or post partum hemorrhage or postpartum haemorrhage or post partum haemorrhage).ti,ab. or obstetric haemorrhage.mp.
18	obstetric labor, premature.mp. or exp Obstetric Labor, Premature/ or (premature labor or premature labour or preterm labor or preterm labour or preterm birth).mp.
19	exp Cesarean Section, Repeat/ or cesarean.mp. or exp Cesarean Section/ or (caesarean or cesarean or caesarian or cesarian or cesarien or caesarien or c-section or c section).mp.
20	exp Extraction, Obstetrical/ or exp Obstetrical Forceps/ or ((operative or instrumental or assisted or forcep* or ventouse* or vacuum*) adj1 (deliver* or birth*)).mp.
21	low birth weight.mp. or exp Infant, Low Birth Weight/ or (low birth weight* adj4 very low birth weight*).mp.
22	exp Infant, Small for Gestational Age/ or small for gestational age.mp. or (small adj3 gestational age).mp.
23	(intra?uterine growth adj2 (restriction* or retardation)).mp. or iugr.ti,ab.

24	fetal growth retardation.mp. or exp Fetal Growth Retardation/ or (fetal growth adj2 (restriction? or retardation)).mp.
25	postpartum depression.mp. or exp Depression, Postpartum/
26	((postpartum* or post partum* or post-partum* or postnatal* or post natal* or post-natal* or perinatal* or peri natal* or peri-natal* or puerp*) and (depress* or dysthymi* or adjustment disorder* or mood disorder* or affective disorder*)).mp.
27	(((postpartum* or post partum* or post-partum* or postnatal* or post natal* or post-natal or perinatal* or peri natal* or peri-natal* or puerp*) and (psychos#s or psychotic)) or psychosis after childbirth).mp.
28	(((third or fourth or 3rd or 4th) adj degree) and tear*).mp.
29	(((anal near adj2 sphincter) or (rectal adj mucosa) or rectum or (anal adj epithelium) or anus or (recto?vaginal adj2 fistulae) or (anorectal adj mucosa) or (anal adj skin)) and (tear* or injur* or damage* or lacerat* or rupture* or trauma)).mp. [mp=title, book title, abstract, original title, name of substance word, subject heading word, floating sub-heading word, keyword heading word, organism supplementary concept word, protocol supplementary concept word, rare disease supplementary concept word, unique identifier, synonyms]
30	((obstetric* and anal and sphincter and injur*) or (anal and sphincter and injur*)).mp.
31	(exp Pregnancy/ or exp Obstetrics/ or (pregnan* or obstetric*).mp.) and (exp Cholestasis/ or exp Cholestasis, Intrahepatic/)
32	1 or 2 or 3 or 4 or 5 or 6 or 7 or 8 or 9 or 10 or 11 or 12 or 13 or 14 or 15 or 16 or 17 or 18 or 19 or 20 or 21 or 22 or 23 or 24 or 25 or 26 or 27 or 28 or 29 or 30 or 31
33	exp Diabetes Mellitus, Type 2/ or diabetes type 2.mp. or Type 2/ or (MODY or NIDDM or T2DM or T2D).mp. or (non insulin* depend* or noninsulin* depend* or noninsulin?depend* or non insulin?depend*).mp. or ((typ? 2 or typ? II or typ?2 or typ?II) adj3 diabet*).mp. or (((late or adult* or matur* or slow or stabl*) adj3 onset) and diabet*).mp.
34	exp hypertension/ or hypertens$.mp. or exp blood pressure/ or (blood pressure or bloodpressure).mp.

35	33 or 34
36	systematic review.mp.
37	(systematic$ adj2 (review$ or overview)).ti,ab.
38	(systematic$ adj5 review$).tw,sh.
39	meta-analysis.mp. or exp meta-analysis/
40	36 or 37 or 38 or 39
41	32 and 35 and 40

References

1. Legistation UK-https://www.legislation.gov.uk/ukpga/1992/29/section/1.
2. National Institute for Health and Care Excellence. NICE Guideline [NG133]: Hypertension in pregnancy: diagnosis and management. 2019. https://www.nice.org.uk/guidance/ng133/chapter/Recommendations#management-of-gestational-hypertension.
3. Royal college of obstetrician and gynaecologists,Placenta praevia, placenta accreta and

vasa praevia,2018

https://www.rcog.org.uk/globalassets/documents/patients/patient-information-leaflets/pregnancy/pi-placenta-praevia-placenta-accreta-and-vasa-praevia.pdf.
4. Workalemahu T, Enquobahrie DA, Gelaye B, Thornton TA, Tekola-Ayele F, Sanchez SE, et al. Abruptio placentae risk and genetic variations in mitochondrial biogenesis and oxidative phosphorylation: replication of a candidate gene association study. American journal of obstetrics and gynecology. 2018;219(6):617. e1-. e17.
5. Martinelli KG, Garcia ÉM, Santos Neto ETd, Gama SGNd. Advanced maternal age and its association with placenta praevia and placental abruption: a meta-analysis. Cadernos de saude publica. 2018;34.
6. Royal college of obstetrician and gynaecologists,The Management of Nausea andVomiting

of Pregnancy and Hyperemesis Gravidarum

Green-top Guideline No. 69,2016

https://www.rcog.org.uk/globalassets/documents/guidelines/green-top-guidelines/gtg69-hyperemesis.pdf.
7. BMJ-Best practice,Gestational diabetes mellitus,2021

https://bestpractice.bmj.com/topics/en-gb/665.
8. Royal college of obstetrician and gynaecologists,,Ectopic pregnancy,2016

https://www.rcog.org.uk/en/patients/patient-leaflets/ectopic-pregnancy/.
9. Royal college of obstetrician and gynaecologists,,Molar pregnancy and gestational trophoblastic disease,2019

https://www.rcog.org.uk/en/patients/patient-leaflets/gestational-trophoblastic-disease-gtd/#:~:text=Molar%20pregnancy%20is%20an%20abnormal,from%20your%20uterus%20(womb).

10. Gestational trophoblastic disease,Cancer research UK https://www.cancerresearchuk.org/about-cancer/gestational-trophoblastic-disease-gtd accessed in Feb,2022.

11. Royal college of obstetrician and gynaecologists,,Multiple pregnancy: having more than one baby,2021

https://www.rcog.org.uk/en/patients/patient-leaflets/multiple-pregnancy-having-more-than-one-baby/.

12. Royal college of obstetrician and gynaecologists,Heavy bleeding after birth (postpartum haemorrhage,,2016

https://www.rcog.org.uk/en/patients/patient-leaflets/heavy-bleeding-after-birth-postpartum-haemorrhage/.

13. World health organisation

Preterm birth,2018

https://www.who.int/news-room/fact-sheets/detail/preterm-birth.

14. National maternal and Perinatal Audit- https://maternityaudit.org.uk/Audit/Charting/ClinicalByMeasure. 2017/2018.

15. World health organisation

Low birth weight,2019

-https://www.who.int/data/nutrition/nlis/info/low-birth-weight#:~:text=Low%20birth%20weight%20has%20been,2500%20grams%20(5.5%20pounds).

16. Royal college of obstetrician and gynaecologists,Small-for-Gestational-Age Fetus, Investigation and Management (Green-top Guideline No. 31),2013

https://www.rcog.org.uk/en/guidelines-research-services/guidelines/gtg31/.

17. National institute of health and care excellence,Depression - antenatal and postnatal,2020,https://cks.nice.org.uk/topics/depression-antenatal-postnatal/.

18. National institute of health and care excellence,Antenatal and postnatal mental health: clinical management and service guidance,2020, https://www.nice.org.uk/guidance/cg192/chapter/Introduction.

19. Royal college of obstetrician and gynaecologists Care of a third- or fourth-degre, e tear that occurred during childbirth (OASI),2019 https://www.rcpsych.ac.uk/mental-health/problems-disorders/postpartum-psychosis.

20. Royal college of obstetrician and gynaecologists ,Obstetric Cholestasis,(Green-top Guideline No. 43),2014

https://www.rcog.org.uk/globalassets/documents/patients/patient-information-leaflets/pregnancy/pi-obstetric-cholestasis.pdf

21. Pelvic girdle pain and pregnancy -<https://www.rcog.org.uk/for-the-public/browse-all-patient-information-leaflets/pelvic-girdle-pain-and-pregnancy/-assessed 27/05/2022>.

Singh M, *et al. BMJ Open* 2022; 12:e066476. doi: 10.1136/bmjopen-2022-066476
