# Supplementary material for: Association of pregnancy complications/risk factors with the development of future long-term health conditions in women: overarching protocol for umbrella reviews
Source: BMJ Open. 2022 Dec 29;12(12):e066476. doi: 10.1136/bmjopen-2022-066476 (PMC9806074; doi:10.1136/bmjopen-2022-066476)
Supplement: Author's manuscript [file bmjopen-2022-066476.draft_revisions.pdf]

BMJ Open is committed to open peer review. As part of this commitment we make the peer review history of every article we publish publicly available.

When an article is published we post the peer reviewers' comments and the authors' responses online. We also post the versions of the paper that were used during peer review. These are the versions that the peer review comments apply to.

The versions of the paper that follow are the versions that were submitted during the peer review process. They are not the versions of record or the final published versions. They should not be cited or distributed as the published version of this manuscript.

BMJ Open is an open access journal and the full, final, typeset and author-corrected version of record of the manuscript is available on our site with no access controls, subscription charges or pay-per-view fees (http://bmjopen.bmj.com).

If you have any questions on BMJ Open's open peer review process please email info.bmjopen@bmj.com

BMJ Open

The association of pregnancy complications/risk factors with the development of future health conditions in women : Overarching protocol for umbrella reviews

Journal:	*BMJ Open*
Manuscript ID	bmjopen-2022-066476
Article Type:	Protocol
Date Submitted by the Author:	18-Jul-2022
Complete List of Authors:	Singh, Megha; University of Birmingham, Applied health research<br>Crowe, Francesca; University of Birmingham, Institute of Applied Health Research<br>Thangaratinam, Shakila; Queen Mary University of London, BARC (Barts Research Centre for Women's Health), Blizard Institute, Barts and the London School of Medicine and Dentistry; University of Birmingham, Institute of Metabolism and Systems Research<br>Abel, Kathryn ; Institute of Brain Behaviour and Mental Health, Medical and Human Sciences; Manchester Mental Health & Social Care Trust<br>Black, Mairead; University of Aberdeen,<br>Okoth, Kelvin; University of Birmingham Edgbaston Campus, Institute of Applied Health Research<br>Riley, Richard; Keele University,<br>Eastwood, Kelly-Ann; University Hospitals Bristol and Weston NHS Foundation Trust<br>Hope, Holly; Manchester University,<br>Wambua, Steven; University of Birmingham, Applied health research<br>Healey, Jemma ; University of Aberdeen, 6. Aberdeen Centre for Women's Health Research, School of Medicine, Medical Science and Nutrition,<br>Lee, Siang Ing; University of Birmingham Institute of Applied Health Research,<br>Phillips, Katherine; University of Birmingham, Applied health research<br>Vowles, Zoe; Guy's and St. Thomas' NHS Foundation Trust<br>Cockburn, Neil; University of Birmingham, Applied health research<br>Moss, Ngawai; Queen Mary University of London<br>Nirantharakumar, Krishnarajah; University of Birmingham, Institute of Applied Health Research
Keywords:	Hypertension < CARDIOLOGY, ONCOLOGY, MENTAL HEALTH

SCHOLARONE™
Manuscripts

Title

The association of pregnancy complications/risk factors with the development of future health conditions in women: Overarching protocol for umbrella reviews

Investigators and Institutional Affiliations

Dr Megha Singh,[1] Dr Francesca Crowe,[1] Professor Shakila Thangaratinam,[2, 3] Professor Kathryn Abel,[4, 5] Dr Mairead Black,[6] Dr Kelvin Okoth,[1] Professor Richard Riley,[7] Dr Kelly-Ann Eastwood,[8, 9] Dr Holly Hope,[4] Mr Steven Wambua,[1] Ms Jemma Healey,[6] Dr Siang Ing Lee,[1] Dr Katherine Phillips,[1] Ms Zoe Vowles,[10] Dr Neil Cockburn,[1] Ms Ngawai Moss,[11] Professor Krishnarajah Nirantharakumar[1]

Institutional Affiliations

1. Institute of Applied Health Research, University of Birmingham, Birmingham, UK
2. WHO Collaborating Centre for Global Women's Health, Institute of Metabolism and Systems Research, University of Birmingham, Birmingham, UK
3. Department of Obstetrics and Gynaecology, Birmingham Women's and Children's NHS Foundation Trust, Birmingham, UK
4. Centre for Women's Mental Health, Division of Psychology and Mental Health, School of Health Sciences, Faculty of Biology Medicine & Health, The University of Manchester, UK
5. Greater Manchester Mental Health NHS Foundation Trust, Manchester, UK
6. Aberdeen Centre for Women's Health Research, School of Medicine, Medical Science and Nutrition, University of Aberdeen, UK
7. Centre for Prognosis Research, School of Primary, Community and Social Care, Keele University, Staffordshire ST5 5BG
8. Centre for Public Health, Queen's University of Belfast, UK
9. St Michael's Hospital, University Hospitals Bristol NHS Foundation Trust, UK
10. Guy's and St. Thomas' NHS Foundation Trust, UK
11. Patient and public representative

Email Address of the Authors

Dr Megha Singh-m.singh.6@bham.ac.uk

Dr Francesca Crowe F.Crowe@bham.ac.uk

Professor Shakila Thangaratinam S.Thangaratinam.1@bham.ac.uk

Professor Kathryn Abel kathryn.m.abel@manchester.ac.uk

Dr Mairead Blackmairead.black@abdn.ac.uk

Dr Kelvin Okoth K.Okoth@bham.ac.uk

Professor Richard Riley r.riley@keele.ac.uk

Dr Kelly-Ann Eastwood k.eastwood@qub.ac.uk

Dr Holly Hope holly.hope@manchester.ac.uk

For peer review only - http://bmjopen.bmj.com/site/about/guidelines.xhtml

For peer review only

Mr Steven Wambua ssw107@student.bham.ac.uk

Ms Jemma Healey jemma.healey@abdn.ac.uk

Dr Siang Ing Lee S.I.Lee@bham.ac.uk

Dr Katherine Phillips k.phillips.1@bham.ac.uk

Ms Zoe Vowles Zoe.Vowles@gstt.nhs.uk

Dr Neil Cockburn n.cockburn@bham.ac.uk

Ms Ngawai Moss ngawai@btinternet.com

Professor Krishnarajah Nirantharakumar " K.Nirantharan@bham.ac.uk

Corresponding Author- Dr. Megha Singh, Applied health research, University of Birmingham, UK. Email-m.singh.6@bham.ac.uk

Word count – 2720

Key Words-Pregnancy complications, Cancer, chronic pain, Hypertension, Diabetes, Autoimmune conditions

Abstract

Introduction

With good medical care, most pregnancy complications like pre-eclampsia, gestational diabetes etc. resolve after childbirth. However, pregnancy complications are known to be associated with an increased risk of new long-term health conditions for the women later in life, such as cardiovascular disease.

This umbrella review aims to summarise systematic reviews evaluating the association between pregnancy complications and five long-term health conditions: metabolic health conditions (diabetes and hypertension), cancer, autoimmune conditions, mental health conditions and functional disorders.

Methods and analysis

We will conduct searches in Medline, Embase and the Cochrane database of systematic reviews without any language restrictions. We will include systematic reviews with or without meta-analyses that studied the association between pregnancy complications and the future risk of the five long-term health conditions in women. Pregnancy complications were identified from existing core outcome sets for pregnancy and consultation with experts.

Two reviewers will independently screen the articles Data will be synthesised with both narrative and quantitative methods. Where a meta-analysis has been carried out, we will report the combined effect size from individual studies. For binary data, pooled odds ratios with 95% confidence intervals (CIs) will be presented. For continuous data, we will use the mean difference with 95% CIs. The findings will be presented in forest plots to assess

For peer review only - http://bmjopen.bmj.com/site/about/guidelines.xhtml

heterogeneity. Methodological quality of the studies will be evaluated with AMSTAR 2 tool and the Cochrane risk of bias tool. The corrected covered area method will be used to assess the impact of overlap in reviews.

The findings from the umbrella review will be used to inform the design of prediction models, which will predict the risk of women developing these five health conditions following a pregnancy complication.

Ethics and dissemination

No ethical approvals required. Findings will be disseminated through publications in peer reviewed journals and conference presentations.

For peer review only

Strengths and Limitations

1-These umbrella reviews will generate highest level of evidence by combining the results from systematic reviews in this topic.

2-This review will include reviews from all languages as no restriction of language is being implied

3-Screening of studies, data extraction and quality appraisal will be done by two reviewers.

4-Same data extraction and quality appraisal forms will be used across the reviews and will be piloted before use.

5-For few conditions which are rare, there might not be a systematic review evidence and hence would not be included in this review which is the limitation of this review.

Detailed Research Plan

Introduction

Although maternal deaths have decreased globally by 38 percent in the last two decades due to improved and accessible medical care, the occurrence of pregnancy complications has seen an increasing trend (1-4). For instance the incidence of hypertensive disorders of pregnancy increased from 16 to 18 million globally, which equates to a total of 11 % between 1990 and 2019,while prevalence of gestational diabetes has also increased during the same time period alongside rising levels of obesity and inactivity (5).

Globally 295 000 maternal deaths occurred in 2017, equivalent to 810 deaths per day, from preventable causes related to pregnancy and childbirth(6, 7).Complications arising during

pregnancy and childbirth continue to be leading causes of maternal deaths, often attributed to postpartum haemorrhage or hypertensive disorders during pregnancy(8).

Most pregnancy complications like hypertensive disorders and gestational diabetes resolve after birth, however, they are associated with increased risk of complications in future pregnancies as well as long term physical and mental health condition. During pregnancy associated with complications or risk factors, the maternal organs undergo significant physiological changes, such as increased cardiac output and inflammatory response, which can be re-activated by age related changes in later life, resulting in development of long-term health condition (9-12).

Women who have experienced pre-eclampsia have an increased risk of developing type 2 diabetes along with increased risk of hypertension, ischaemic heart disease, stroke and venous thromboembolism(13-16). Depression and anxiety disorders are common after Miscarriage, stillbirth and preterm births(17-19). Studies have also identified pregnancy complications such as miscarriage and pre-term birth are associated with future risk of breast cancer (20, 21)

While it is well established that gestational diabetes increases the risk of developing type 2 diabetes by tenfold, it is also associated with an increased risk of cardiovascular disease and cancer in in later life.(22, 23) Even complications often considered less serious such as hyperemesis was associated with a 70% increased risk of developing rheumatoid arthritis Relationship between miscarriage and future development of autoimmune diseases have also been identified.(24, 25)

Despite there being a number of systematic reviews that have described the link between adverse complications during pregnancy and a multitude of outcomes. the awareness low as and there is need of robust synthesis of this evidence for use by health service researchers, health professionals and policy makers to establish the relationship and also to resolve the conflicting evidences.

We have previously published an umbrella review studying the association between pregnancy complications and cardiovascular diseases (26). In this umbrella review protocol, we describe our objectives and methods to investigate the association between pregnancy complications and five other groups of long-term health conditions, namely metabolic conditions (diabetes and hypertension), cancer, autoimmune conditions, mental health conditions and functional conditions. The umbrella reviews will aid in collating the necessary evidence to delineate the association between adverse pregnancy complications and subsequent occurrence of these health conditions. This will help develop early detection strategies and inform the prognostic factors to develop risk prediction model to predict future occurrence of these long-term health conditions.

Protocol development

This Umbrella review protocol was developed following Joanna Briggs Institute Methodology for Umbrella Reviews and Preferred Reporting Items for Systematic Reviews and Meta-Analyses(27). The anticipated start is March 2022. Any changes will be reported in

For peer review only - http://bmjopen.bmj.com/site/about/guidelines.xhtml

detail. The protocol has been registered with Prospero Registration number is: CRD42022323718

Aims and objectives

Aims

This umbrella review will identify, appraise, and consolidate higher level evidence in the form of systematic reviews and/or meta-analyses into a single readable/usable document and provide recommendations and policy for future research. To review existing evidence from systematic reviews and epidemiological studies of pregnancy complications/risk factors associated with a woman's future risk of group of long-term health conditions.

Objectives

To identify and appraise higher level evidence (systematic reviews and meta-analysis also primary studies where applicable) reporting on the association between pregnancy complications/ risk factors (Table 1) and future risk of

1) Metabolic health conditions
2) Functional health conditions
3) Autoimmune conditions
4) Cancers
5) Mental health conditions

The health conditions included in each of the above groups are listed below(Table 2).

Research plan/Methods

The reviews aim to combine all the available evidence and present with one concrete inference. It will primarily include systematic reviews (with or without meta-analyses). Where there is no existing systematic review, the research team will undertake a scoping review to assess whether to conduct a new systematic review. The reporting of this umbrella review will be in accordance to the PRISMA guidelines (28).

Study design

Systematic reviews of observational and interventional studies with or without meta-analysis that have assessed the association between the pregnancy complications/risk factors and future risk of health conditions in women will be considered for this umbrella review. Primary studies will be considered where appropriate (29, 30).

Population

The population will include pregnant women no age restriction.

Exposures

For peer review only - http://bmjopen.bmj.com/site/about/guidelines.xhtml

Exposures are adverse pregnancy complications/risk factors which will be identified by scoping searches and consultation with experts. The list of pregnancy complications and risk factors identified to date are listed below in Table 1 and the definitions are listed in (Appendix Table 1). Depending upon outcome in question few the exposures might not be considered.

Table 1: Pregnancy complications and risk factors

1. **Pregnancy loss**
   - Miscarriage/ Recurrent miscarriage/ Spontaneous pregnancy loss
   - Stillbirth
2. **Hypertensive disorders of pregnancy**
   - Gestational hypertension
   - Pre-eclampsia- early or late onset
   - Recurrent pre-eclampsia
   - Eclampsia
   - HELLP (Hemolysis, Elevated Liver enzymes and Low Platelets) syndrome
3. **Placental disorders**
   - Placenta previa
   - Placental abruption
   - Placenta accreta
   - Placenta percreta
4. **Hyperemesis gravidarum**
5. **Gestational diabetes mellitus**
6. **Ectopic pregnancy**
7. **Molar pregnancy/ Choriocarcinoma**
8. **Multiple pregnancy/Twin-pregnancies/ Multiple gestation**
9. **Obstetric haemorrhage (postpartum)**
10. **Pre-term birth/ Recurrent pre-term birth**
11. **Mode of birth: Caesarean, Instrumental**
12. **Low birth weight**
    - Low birth weight
    - Small for gestational age
    - Intra-uterine growth retardation / intra-uterine growth restriction
    - Fetal growth restriction
13. **post-partum depression**
14. **Puerperal psychosis**
15. **Perineal trauma-3rd and 4th degree tear**
16. **Obstetric cholestasis**
17. **Pelvic girdle pain**

Comparator

Pregnant women without the exposure of interest will be controls.

Outcomes

For peer review only - http://bmjopen.bmj.com/site/about/guidelines.xhtml

The outcome of interest is the following 5 groups of long-term health conditions.

Table 2: List of Health conditions(outcomes)

Condition	
**Mental health**	Serious mental illness-affective psychosis (bipolar, mania) and non-affective psychoses (schizophrenia, paranoia and psychoses nos). Common mental illness-mood disorders (depression, dysthymia) and neurotic (anxiety, PTSD, OCD etc) disorders
**Functional conditions**	fibromyalgia, chronic pain, chronic back pain, tension headache, irritable bowel syndrome, interstitial cystitis, vulvodynia
**Autoimmune disorders**	psoriasis, vitiligo, alopecia areata, systemic lupus erythematosus, psoriatic arthritis, ankylosing spondylitis, rheumatoid arthritis, Sjogren's syndrome, systemic sclerosis, coeliac disease, Crohn's disease, ulcerative colitis, multiple sclerosis, Grave's disease, Hashimoto's disease, type 1 diabetes, myasthenia gravis
**Metabolic conditions**	diabetes Type 2 and hypertension.
**Cancers**	lung, breast, colorectal, cervix, cancer of unknown primary, pancreas, ovary, uterus/endometrium, brain, (other central nervous system) and intracranial tumours, liver, melanoma skin cancer, lymphoma, kidney, thyroid, leukaemia.

For peer review only

Search Strategy

We will conduct searches in Medline, Embase and the Cochrane database of systematic reviews from inception. We will be looking for systematic reviews with or without meta-analyses that examines the association between pregnancy complications and future risk of the long-term health condition in women.

There will be no restriction of language or setting when selecting the studies.

The search strategy will be developed using subject headings and free text keywords using the concepts for pregnancy complications and risk factors listed in Table 1 and for the specific disease in the group of conditions to be studied.

The reference list of included studies will also be searched. The search terms for Medline are provided in Appendix Table 2 and 3. These will be modified for use in other databases.

A two-pronged approach will be adapted, firstly search strategy will be restricted to systematic reviews to identify which pregnancy complications/ risk factors will have evidence from systematic reviews. We will subsequently run a search strategy for primary studies where necessary.

Searches will be updated periodically to identify emerging systematic reviews.

Study selection

Once the literature search is completed, reference management software will be used to manage the identified literature (e.g., Endnote, Mendeley or Reference Manager). After removing duplicate studies, two independent reviewers will conduct the title and abstract screening and ineligible studies will be excluded.

For peer review only - http://bmjopen.bmj.com/site/about/guidelines.xhtml

Full text screening of eligible studies will be conducted by two independent reviewers and a third senior reviewer will be consulted to resolve any discrepancy. The full text will be translated if non-English studies are identified.

The list of excluded studies will be maintained with the reasons for exclusion documented. The details of the steps involved in study selection will be reported using the PRISMA flowchart (31).

Exclusion criteria

The following types of publications will not be included: protocols, review articles, conference abstracts, guidelines, consensus, documents or expert position papers, summaries, comments, letters, and brief reports. The reviews that include theoretical studies or text or opinion as their primary source of evidence and all narrative reviews that provided no quantitative data will not be included.(27)

Quality assessment

A Measurement Tool to Assess systematic Reviews 2 (AMSTAR 2) will be used to assess the methodological quality by two independent reviewers. Out of the 16 points of the AMSTAR 2 tool, one point will be awarded for each of the criteria met (32). The reviews will be rated at high, moderate, low or critically low quality.

Non-randomised trials will be rated as having low, moderate, serious or critical or no information on which to base a judgement about the risk of bias. For randomised trials they will be rated as low, some concerns or high risk of bias.

To resolve any disagreements, a third reviewer will be consulted.

Update of existing reviews

Up to 50% of systematic reviews are out of date after 5.5 years (33). The recommended methods for updating existing systematic reviews and meta-analyses will be used where a need for update is identified (28, 34). In case the reviews need to be updated, only high and moderate quality reviews will be eligible for updates (35).

Overlap

Overlapping reviews refers to a situation where two or more reviews are examining the same research question and may include the same primary studies. The degree of overlap will be presented graphically using a citation matrix. A citation matrix is a plot of the included primary studies in the rows and systematic reviews in the columns. Overlap will be quantified by the method of corrected covered area (36). Quality rating will be used as the selection criteria where higher-quality reviews will be considered over lower-quality reviews.

Data extraction

A standardised data extraction form will be used. The data extraction form will be piloted prior to use. Data will be extracted from the final list of included studies which will then be checked by the second reviewers in case of conflict, a third reviewer will be consulted.

For peer review only - http://bmjopen.bmj.com/site/about/guidelines.xhtml

The data will be extracted in the following template using Microsoft Excel.

1. Study ID
2. Author/s
3. Year of publication
4. Geographical area
5. Aim of the review
6. Database searched
7. Search period
8. Population
9. Heath care setting
10. Exposures
11. Comparator
12. Outcomes
13. Covariates
14. Study design(s)
15. Definition of exposure
16. Definition of outcome
17. Data synthesis method
18. Quality assessment tool
19. Quality of the included primary studies as assessed by review authors
20. Number of studies included in qualitative analysis (narrative synthesis where meta-analysis was not done / possible by the review authors)
21. Number of meta-analyses
22. Number of studies included in each meta-analysis
23. Summary estimates of each meta-analysis and its related 95% confidence intervals
24. Author's conclusion
25. Review limitation
26. Additional comments

Recently published studies will be added into the narration of the reviews where no meta-analysis has been performed.

Data analysis

Study characteristics will be presented in a tabular format. The study will synthesise the data in both narrative and quantitative methods. Where a meta-analysis has been carried out, we will report the summary result. Where the review does not provide a summary result, we will explore the key findings and use these to inform a narrative overview of the key findings. Based on the data extracted from individual studies, systematic review or meta-analysis, data may need to be converted before pooling. For binary data, pooled odds ratios with 95% confidence intervals (CIs) will be presented. For continuous data, we will use the mean difference or standardised mean difference with 95% CIs.

The findings will be presented in forest plots to assess the heterogeneity of the study findings. The I^2 statistic will be used to evaluate statistical heterogeneity. All statistical analysis will be conducted using Stata and Microsoft Excel.

For peer review only - http://bmjopen.bmj.com/site/about/guidelines.xhtml

Ethics and dissemination

No ethical approvals required. Findings will be disseminated through publications in peer reviewed journals and conferences.

Patient Public involvement

Patient and public involvement (PPI) representatives (RP and NM) were involved in formulating the research question and study design. They have also played key role in collaboration with clinicians and researchers to identify and consider the list of pregnancy complications and health outcomes in the study. NM has been part of our regular meetings and also provided input to improve the manuscript. They will also play a key role in disseminating the results once the reviews have been undertaken.

Conflict of Interest

No conflict of interest reported.

Acknowledgements

Patient representatives and MuM-PreDiCT team

Authors contributions

MS was responsible for drafting the initial manuscript.KN, FC, ST, MB, KAE, HH, JH, SW, ZW, KP, NM, NC, SIL, ST were responsible for revising the manuscript critically for important intellectual content. The authors have approved the final submitted version and are accountable for all aspects of the work.

Funding

This work was funded by the Strategic Priority Fund "Tackling multimorbidity at scale" programme [grant number-MR/W014432/1 delivered by the Medical Research Council and the National Institute for Health and Care Research in partnership with the Economic and Social Research Council and in collaboration with the Engineering and Physical Sciences Research Council.

References

1. Kramer MS. The epidemiology of adverse pregnancy outcomes: an overview. J Nutr. 2003;133(5 Suppl 2):1592s-6s.
2. Zhang F, Dong L, Zhang CP, Li B, Wen J, Gao W, et al. Increasing prevalence of gestational diabetes mellitus in Chinese women from 1999 to 2008. Diabet Med. 2011;28(6):652-7.
3. Lavery JA, Friedman AM, Keyes KM, Wright JD, Ananth CV. Gestational diabetes in the United States: temporal changes in prevalence rates between 1979 and 2010. Bjog. 2017;124(5):804-13.
4. Bornstein E, Eliner Y, Chervenak FA, Grünebaum A. Concerning trends in maternal risk factors in the United States: 1989–2018. EClinicalMedicine. 2020;29:100657.

For peer review only - http://bmjopen.bmj.com/site/about/guidelines.xhtml

5. Wang, W., Xie, X., Yuan, T. et al. Epidemiological trends of maternal hypertensive disorders of pregnancy at the global, regional, and national levels: a population-based study. BMC Pregnancy Childbirth 21, 364 (2021). https://doi.org/10.1186/s12884-021-03809-2. 2021.
6. World health organisation,Maternal mortalityhttps://www.who.int/news-room/fact-sheets/detail/maternal-mortality(16/02/2022) ,(assessed Feb,2022).
7. Dr Lale Say M, Doris Chou, MD,Alison Gemmill, MPH,Özge Tunçalp, MD,Ann-Beth Moller, MSc,Jane Daniels, PhD,A Metin Gülmezoglu, MD,Marleen Temmerman, MD,Leontine Alkema, PhD. Global causes of maternal death: a WHO systematic analysis. 2014;VOLUME 2,(ISSUE 6, E323-E333).
8. World health organisation,maternal deaths fact sheet,https://www.who.int/news-room/fact-sheets/detail/maternal-mortality,22nd february,2022.
9. Sattar N, Greer IA. Pregnancy complications and maternal cardiovascular risk: opportunities for intervention and screening? BMJ. 2002 Jul 20;325(7356):157-60. doi: 10.1136/bmj.325.7356.157. PMID: 12130616; PMCID: PMC1123678.,(assessed Feb,2022). 2002.
10. Neiger R. Long-Term Effects of Pregnancy Complications on Maternal Health: A Review. Journal of Clinical Medicine. 2017;6(8):76.
11. Panaitescu AM PM, Ciobanu AM, Gica N, Cimpoca-Raptis BA. . Pregnancy Complications Can Foreshadow Future Disease-Long-Term Outcomes of a Complicated Pregnancy. Medicina (Kaunas). 2021;57(12):1320. Published 2021 Dec 1. doi:10.3390/medicina57121320. 2021.
12. Williams D. Pregnancy: a stress test for life. Curr Opin Obstet Gynecol. 2003 Dec;15(6):465-71. doi: 10.1097/00001703-200312000-00002. PMID: 14624211..,(assessed Feb,2022)

2003.
13. Bellamy L, Casas JP, Hingorani AD, Williams DJ. Pre-eclampsia and risk of cardiovascular disease and cancer in later life: systematic review and meta-analysis. Bmj. 2007;335(7627):974.
14. Robbins CL, Hutchings Y, Dietz PM, Kuklina EV, Callaghan WM. History of preterm birth and subsequent cardiovascular disease: a systematic review. Am J Obstet Gynecol. 2014;210(4):285-97.
15. Rich-Edwards JW. Reproductive health as a sentinel of chronic disease in women. Womens Health (Lond). 2009;5(2):101-5.
16. Wu P, Kwok CS, Haththotuwa R, Kotronias RA, Babu A, Fryer AA, et al. Pre-eclampsia is associated with a twofold increase in diabetes: a systematic review and meta-analysis. Diabetologia. 2016;59(12):2518-26.
17. Knight M BK, Tuffnell D, Shakespeare J, Kotnis R, Kenyon S, Kurinczuk JJ (Eds.) on behalf of MBRRACE-UK. Saving Lives, Improving Mothers' Care - Lessons learned to inform maternity care from the UK and Ireland Confidential

Enquiries into Maternal Deaths and Morbidity 2015-17. Oxford: National Perinatal Epidemiology Unit, University of

Oxford 2019

2015-17.
18. Brier N. Anxiety after miscarriage: a review of the empirical literature and implications for clinical practice. Birth. 2004;31(2):138-42.
19. Anderson C, Cacola P. Implications of preterm birth for maternal mental health and infant development. MCN: The American Journal of Maternal/Child Nursing. 2017;42(2):108-14.
20. Brind J, Chinchilli VM, Severs WB, Summy-Long J. Induced abortion as an independent risk factor for breast cancer: a comprehensive review and meta-analysis. J Epidemiol Community Health. 1996;50(5):481-96.
21. Wingo PA, Newsome K, Marks JS, Calle EE, Parker SL. The risk of breast cancer following spontaneous or induced abortion. Cancer Causes Control. 1997;8(1):93-108.
22. Burlina S, Dalfrà MG, Chilelli NC, Lapolla A. Gestational Diabetes Mellitus and Future Cardiovascular Risk: An Update. International Journal of Endocrinology. 2016;2016:2070926.

For peer review only - http://bmjopen.bmj.com/site/about/guidelines.xhtml

23. Daly B, Toulis KA, Thomas N, Gokhale K, Martin J, Webber J, et al. Increased risk of ischemic heart disease, hypertension, and type 2 diabetes in women with previous gestational diabetes mellitus, a target group in general practice for preventive interventions: a population-based cohort study. PLoS medicine. 2018;15(1):e1002488.
24. Brennan P, Bankhead C, Silman A, Symmons D, editors. Oral contraceptives and rheumatoid arthritis: results from a primary care-based incident case-control study. Seminars in arthritis and rheumatism; 1997: Elsevier.
25. Jørgensen KT, Pedersen BV, Jacobsen S, Biggar RJ, Frisch M. National cohort study of reproductive risk factors for rheumatoid arthritis in Denmark: a role for hyperemesis, gestational hypertension and pre-eclampsia? Annals of the rheumatic diseases. 2010;69(2):358-63.
26. Okoth K, Chandan JS, Marshall T, Thangaratinam S, Thomas GN, Nirantharakumar K, et al. Association between the reproductive health of young women and cardiovascular disease in later life: umbrella review. BMJ. 2020;371.
27. Institute TJB. Joanna Briggs Institute Reviewers' Manual: 2014 edition / Supplement. 2014.
28. Smith V, Devane D, Begley CM, Clarke M. Methodology in conducting a systematic review of systematic reviews of healthcare interventions. BMC medical research methodology. 2011;11(1):1-6.
29. Haddaway NR, Land M, Macura B. "A little learning is a dangerous thing": A call for better understanding of the term 'systematic review'. Environment International. 2017;99:356-60.
30. (ed.) GB-Z. Umbrella Reviews: Evidence Synthesis with Overviews of Reviews and Meta-Epidemiologic Studies, chapter 1 page 6.6.
31. Page MJ, McKenzie JE, Bossuyt PM, Boutron I, Hoffmann TC, Mulrow CD, et al. The PRISMA 2020 statement: an updated guideline for reporting systematic reviews. Bmj. 2021;372:n71.
32. Shea BJ, Reeves BC, Wells G, Thuku M, Hamel C, Moran J, et al. AMSTAR 2: a critical appraisal tool for systematic reviews that include randomised or non-randomised studies of healthcare interventions, or both. Bmj. 2017;358:j4008.
33. Bougioukas KI, Liakos A, Tsapas A, Ntzani E, Haidich A-B. Preferred reporting items for overviews of systematic reviews including harms checklist: a pilot tool to be used for balanced reporting of benefits and harms. Journal of clinical epidemiology. 2018;93:9-24.
34. Senn SJ. Overstating the evidence–double counting in meta-analysis and related problems. BMC Medical Research Methodology. 2009;9(1):1-7.
35. Garner P, Hopewell S, Chandler J, MacLehose H, Akl EA, Beyene J, et al. When and how to update systematic reviews: consensus and checklist. BMJ. 2016;354:i3507.
36. Lunny C, Pieper D, Thabet P, Kanji S. Managing overlap of primary study results across systematic reviews: practical considerations for authors of overviews of reviews. BMC Med Res Methodol. 2021;21(1):140.

For peer review only

The association of pregnancy complications/risk factors with the development of future health conditions in women: Overarching protocol for umbrella reviews

Appendix/Supplementary material

Appendix Table 1: Definitions of pregnancy complications and risk factors

Pregnancy Specific factors	Definitions
Miscarriage/ Recurrent miscarriage/ Spontaneous pregnancy loss.	Spontaneous loss of a pregnancy before 24 weeks of gestation.<br>Early miscarriage, before 13 weeks of gestation.<br>Late miscarriage, between 13 and 24 weeks of gestation<br>Recurrent miscarriage is loss of three or more consecutive pregnancies before 24 weeks of gestation. (36)
Stillbirth	child born after the 24th week of pregnancy who did not show any signs of life after being born(37)
Pre-eclampsia- early and late onset<br>Recurrent pre-eclampsia<br><br>Gestational hypertension	New onset of hypertension (over 140 mmHg systolic or over 90 mmHg diastolic) after 20 weeks of pregnancy and the coexistence of 1 or more of the following new-onset conditions:<br>- proteinuria<br>- other maternal organ dysfunction (renal insufficiency, liver involvement, neurological complications, haematological complications)<br>- uteroplacental dysfunction.(38)
Eclampsia<br>HELLP	The new onset of seizures or coma in a pregnant woman with pre-eclampsia<br>HELLP (Hemolysis, Elevated Liver enzymes and Low Platelets) syndrome is a life-threatening pregnancy complication usually considered to be a variant of pre-eclampsia(38)
Gestational hypertension	New hypertension presenting after 20 weeks of pregnancy without significant proteinuria.(38)
Placenta previa<br>Placenta accreta<br>Placenta percreta	Placenta previa is low lying placenta after 20 weeks of pregnancy.(39)<br>Placenta accrete is where placenta is stuck to the uterine muscle.(39)<br>Placenta percreta is a condition where placenta attaches itself and grows through the uterus and potentially to the nearby organs (such as the bladder).(39)
Hyperemesis gravidarum	Severe, protracted nausea and vomiting associated with weight loss of more than 5% of pre pregnancy weight, dehydration and electrolyte imbalances.(40)
Gestational diabetes mellitus	hyperglycaemia in pregnancy that is above diagnostic thresholds for diabetes

For peer review only - http://bmjopen.bmj.com/site/about/guidelines.xhtml

	• a fasting plasma glucose level of 5.6 mmol/litre or above or<br>• a 2-hour plasma glucose level of 7.8 mmol/litre or above.(41)
Ectopic pregnancy	If the fertilised egg fails to move from the fallopian tube into the uterus, where the pregnancy grows and develops and the fertilised egg may implant and start to develop outside the uterus.(42)
Molar pregnancy/ Choriocarcinoma	Abnormal cells or tumours that start in the womb from cells that would normally develop into the placenta are called Gestational trophoblastic disease (GTD)<br>Gestational trophoblastic disease (GTD) forms a group of disorders spanning the conditions of complete and partial molar pregnancies through to the malignant conditions of invasive mole, choriocarcinoma. (43, 44)
Multiple pregnancy/Twin-pregnancies/ Multiple gestation	Term used when one is expecting two or more babies at the same time (twins, triplets or more)(45)
Obstetric / postpartum haemorrhage	Heavy bleeding after birth. PPH can be primary or secondary. Primary PPH -blood loss 500 ml (a pint) or more of blood within the first 24 hours after the birth of the baby. Secondary PPH occurs when there is abnormal or heavy vaginal bleeding between 24 hours and 12 weeks after the birth.(46)
Pre-term birth/ Recurrent pre-term birth	Babies born alive before 37 weeks of pregnancy are completed. extremely preterm (less than 28 weeks),very preterm (28 to 32 weeks),moderate to late preterm (32 to 37 weeks).(47)
Mode of birth: Caesarean, Instrumental	Birth of a singleton baby between 37+0 and 42+6 weeks of gestation, who give birth vaginally with the assistance of instruments(ventouse and forceps births).(48)<br><br>Birth of a singleton baby between 37+0 and 42+6 weeks of gestation, by caesarean section(elective or emergency).(48)
Low birth weight	Weight at birth of < 2500 grams (5.5 pounds).(49)
Small for gestational age<br>Intra-uterine growth retardation<br>Intra uterine growth restriction<br>Fetal growth restriction	Growth restriction implies a pathological restriction of the genetic growth potential. As a result, growth restricted fetuses may manifest evidence of fetal compromise (abnormal Doppler studies, reduced liquor volume).<br>Small for gestational age Term babies with a birth weight below the 10th centile, and below the 2nd centile are provided for reference.(48)<br><br>fetal growth restriction is defined as birth weight <3rd centile. We used severe small for gestational age at birth as a proxy for fetal growth restriction.(50)
Post-partum depression	Depression like symptoms including low mood etc that starts within one or two months or several months of giving birth.

For peer review only - http://bmjopen.bmj.com/site/about/guidelines.xhtml

	Symptoms might last at least two weeks depending on the severity.(51)
Puerperal psychosis	Or Puerperal psychosis is a severe mental illness that starts suddenly in the days, or weeks, after having a baby. Symptoms vary and can include high mood (mania), depression, confusion, hallucinations and delusions.(52)
Perineal trauma-3rd and 4th degree tears	Severe perineal tears extending into the anal sphincter, anal mucosa are known as "third degree" and "fourth degree" tears also known as obstetric anal sphincter injury – OASI)(48, 53)
Pelvic girdle pain (PGP)	Pelvic girdle pain or symphysis pubis dysfunction (SPD).refers to pain in the front and/or the back of your pelvis .It also affect other areas such as the hips or thighs and the sacroiliac joints at the back and/or the symphysis pubis joint at the front.(54)
Obstetric cholestasis	Disorder that affects your liver during pregnancy causing build-up of bile acids in the body. The main symptom is itching of the skin and no rash. The symptoms get better when your baby has been born.(55)

Appendix Table 2. Search terms for pregnancy complications / risk factors (exposure)

Pregnancy complications/risk factors	pregnancy complication*.mp. or exp Pregnancy Complications/
Miscarriage/ Recurrent miscarriage/ Spontaneous pregnancy loss	exp Abortion, Spontaneous/ or ((recurrent adj3 miscarr$) or miscarr$ or early pregnancy loss$).mp.
Stillbirth	(stillbirth or still birth).mp. or exp Stillbirth/ or exp Fetal Death/ or (f?etal death$ or f?etal demise$).mp.
Hypertensive disorders of pregnancy /Gestational hypertension	Hypertension, Pregnancy-Induced/ or (gestational hypertension or (pregnancy adj3 hypertensi$)).mp.
Pre-eclampsia- early or late onset	(preeclampsia or pre-eclampsia).mp. or exp Pre-Eclampsia/
Eclampsia	exp Eclampsia/ or (eclampsia or tox?emia).mp.
HELLP (Homolysis, Elevated Liver enzymes and Low Platelets) syndrome	HELLP.mp. or exp HELLP Syndrome/
Placenta previa	placenta accreta.mp. or exp Placenta Accreta/ or placenta percreta.mp. or placenta increta.mp. or morbidly adherent placenta.mp. or abnormally invasive placenta.mp.
Placental abruption	Placenta$ abruption.mp. or exp Abruptio Placentae/

For peer review only - http://bmjopen.bmj.com/site/about/guidelines.xhtml

Placenta accrete and placenta percreta	placenta pr?eia.mp. or exp placenta previa/ or exp low lying placenta/
Hyperemesis gravidarum	Hyperemesis Gravidarum.mp. or Hyperemesis Gravidarum/ or morning sickness.mp. or exp Morning Sickness/
Gestational diabetes mellitus	(((pregnan$ or gestation$ or prenatal$ or antenatal$ or pre-natal$ or ante-natal$ or maternal$) adj2 diabet$) or gestational diabetes).mp. or exp Diabetes, Gestational/ or GDM.mp.
Ectopic pregnancy	ectopic pregnancy.mp. or exp Pregnancy, Ectopic/ or ((tub$$ adj3 pregnanc$) or (cornual adj3 pregnanc$) or (heterotopic adj3 pregnanc$) or (abdomin$ adj3 pregnanc$) or (extrauterine adj3 pregnanc$) or (interstitial adj3 pregnanc$) or (cervi$ adj3 pregnanc$) or (ovar$ adj3 pregnanc$) or (cesarean scar adj3 pregnanc$)).mp.
Molar pregnancy/ Choriocarcinoma	exp Gestational Trophoblastic Disease/ or gestational trophoblastic.mp. or exp Hydatidiform Mole/ or ((hydatid? adj2 mole?) or (molar adj2 pregnanc?)).mp. exp Choriocarcinoma/ or choriocarcinoma.mp.
Multiple pregnancy/Twin-pregnancies/ Multiple gestation	exp Pregnancy, Multiple/ or ((pregnanc* or gestation*) adj (twin* or triplet* or quadruplet* or quintuplet* or multiple or multi?f?et*)).mp. or (Monochorionic or dichorionic).mp.
Obstetric haemorrhage (postpartum)	exp Postpartum Hemorrhage/ or (postpartum hemorrhage or post partum hemorrhage or postpartum haemorrhage or post partum haemorrhage).ti,ab. or obstetric haemorrhage.mp.
Pre-term birth/ Recurrent pre-term birth	obstetric labor, premature.mp. or exp Obstetric Labor, Premature/ or (premature labor or premature labour or preterm labor or preterm labour or preterm birth).mp.
Mode of delivery: Caesarean or Instrumental	exp Cesarean Section, Repeat/ or cesarean.mp. or exp Cesarean Section/ or (caesarean or cesarean or caesarian or cesarian or cesarien or caesarien or c-section or c section).mp.

For peer review only - http://bmjopen.bmj.com/site/about/guidelines.xhtml

	exp Extraction, Obstetrical/ or exp Obstetrical Forceps/ or ((operative or instrumental or assisted or forcep* or ventouse* or vacuum*) adj1 (deliver* or birth*)).mp.
Low birth weight	low birth weight.mp. or exp Infant, Low Birth Weight/ or (low birth weight* adj4 very low birth weight*).mp.
Small for gestational age	exp Infant, Small for Gestational Age/ or small for gestational age.mp. or (small adj3 gestational age).mp.
Intra-uterine growth retardation / intra-uterine growth restriction	(intra?uterine growth adj2 (restriction* or retardation)).mp. or iugr.ti,ab<br>fetal growth retardation.mp. or exp Fetal Growth Retardation/ or (fetal growth adj2 (restriction? or retardation)).mp.
postpartum depression	postpartum depression.mp. or exp Depression, Postpartum/
Puerperal psychosis	((postpartum* or post partum* or post-partum* or postnatal* or post natal* or post-natal* or perinatal* or peri natal* or peri-natal* or puerp*) and (depress* or dysthymi* or adjustment disorder* or mood disorder* or affective disorder*)).mp.<br><br>(((postpartum* or post partum* or post-partum* or postnatal* or post natal* or post-natal or perinatal* or peri natal* or peri-natal* or puerp*) and (psychos#s or psychotic)) or psychosis after childbirth).mp.
Perineal trauma(3rd and 4th degree)	(((third or fourth or 3rd or 4th) adj degree) and tear*).mp.<br><br>(((anal near adj2 sphincter) or (rectal adj mucosa) or rectum or (anal adj epithelium) or anus or (recto?vaginal adj2 fistulae) or (anorectal adj mucosa) or (anal adj skin)) and (tear* or injur* or damage* or lacerat* or rupture* or trauma)).mp.<br><br>((obstetric* and anal and sphincter and injur*) or (anal and sphincter and injur*)).mp.

For peer review only - http://bmjopen.bmj.com/site/about/guidelines.xhtml

Obstetric cholestasis	(exp Pregnancy/ or exp Obstetrics/ or (pregnan* or obstetric*).mp.) and (exp Cholestasis/ or exp Cholestasis, Intrahepatic/)

Appendix Table 3: Search terms for long-term health conditions (outcomes)

**Autoimmune health conditions**	
**Health conditions**	**Search terms**
Autoimmune conditions	exp Autoimmune Diseases/ or (auto?immune adj2 (disease* or condition*)).mp.
Psoriasis	exp Psoriasis/ or psoria$.mp.
Vitiligo	vitiligo.mp. or exp Vitiligo/ or (leucoderma or leukoderma).mp.
Alopecia areata	alopecia areata.mp. or exp Alopecia Areata/ or (alopecia adj totalis).mp. or (alopecia adj universalis).mp.
Systemic lupus erythematosus	exp Lupus Erythematosus, Systemic/ or systemic lupus.mp. or sle.mp.
Psoriatic arthritis	exp Arthritis, Psoriatic/ or psoriatic.mp. or (psoria? adj (arthriti? or arthropath?)).mp. or ((arthriti? or arthropath?) adj psoria?).mp.
Ankylosing spondylitis	Ankylosing spondylitis.mp. or exp Spondylitis, Ankylosing/
Rheumatoid arthritis	exp Arthritis, Rheumatoid/ or rheumatoid artheritis.mp. or ((rheumatoid or reumatoid or revmatoid or rheumatic or reumatic or revmatic or rheumat$ or reumat$ or revmarthrit$) adj3 (arthrit$ or artrit$ or diseas$ or condition$ or nodule$)).mp.
Sjogern's syndrome	exp Sjogren's Syndrome/ or sjogren.mp.
Coeliac disease	celiac disease.mp. or exp Celiac Disease/ or (c?eliac adj disease*).mp.
Inflammatory bowel disease	exp Inflammatory Bowel Diseases/ or inflammatory bowel.mp.
Ulcerative colitis	ulcerative colitis.mp. or exp Colitis, Ulcerative/
Crohn's disease	Crohn's disease.mp. or exp Crohn Disease/
Multiple sclerosis	multiple sclerosis.mp. or exp Multiple Sclerosis/
Systemic sclerosis	Systemic Sclerosis.mp. or exp Scleroderma, Systemic/ or ((systemic adj scler?) or sclero?).mp. or crest syndrome.mp. or exp CREST Syndrome/
Graves' disease	graves disease.mp. or exp Graves Disease/ or (grave? adj6 (diseas? or thyrotoxicos? or hyperthyr?)).mp.

For peer review only - http://bmjopen.bmj.com/site/about/guidelines.xhtml

Hashimoto's disease	exp Thyroiditis, Autoimmune/ or or autoimmune thyroid.mp. or Hashimoto Disease/
Myasthenia gravis	exp Myasthenia Gravis/ or mysathenia.mp.
Addison's disease	exp Addison Disease/ or addison.mp.
Type 1 diabetes	type 1 diabetes.mp. or exp Diabetes Mellitus, Type 1/ or ("typ? 1 diabet*" or "typ? I diabet*" or "typ?1 diabet*" or "typ?I diabet*").mp. or (("insulin* depend*" or "insulin?depend*") not ("non-insulin* depend*" or "non insulindepend*")).mp. or (IDDM or T1DM or T1D).mp. or autoimmune diabetes.mp.
**Metabolic health conditions**	
Types 2 diabetes	exp Diabetes Mellitus, Type 2/ or diabetes type 2.mp. or Type 2/ or (MODY or NIDDM or T2DM or T2D).mp. or (non insulin* depend* or noninsulin* depend* or noninsulin?depend* or non insulin?depend*).mp. or ((typ? 2 or typ? II or typ?2 or typ?II) adj3 diabet*).mp. or (((late or adult* or matur* or slow or stabl*) adj3 onset) and diabet*).mp.
Hypertension	exp hypertension/ or hypertens$.mp. or exp blood pressure/ or (blood pressure or bloodpressure).mp.
**Cancers**	
Lung (C33-34)	exp Lung Neoplasms/ or lung cancer c33 c34.mp.
Breast (C50)	exp Breast Neoplasms/ or breast cancer.mp. or breast cancer c50.mp.
Cervix	Uterine Cervical Neoplasms/ or cancer cervix.mp. or cervical cancer.mp. or cervical neoplasms.mps or cer$ cancer.mp.
Cancer of unknown primary (c77-c80)	Neoplasms, Unknown Primary/ or cancer of unknown primary.mp.
Pancreas (c25)	exp Pancreatic Neoplasms/ or cancer pancrea$.mp. or Carcinoma, Pancreatic Ductal or pancreas$ carcinoma.mp.
Ovary (c56- c57.4)	Exp Ovarian Neoplasms/ or ((ovarian or ovary or ovar$) adj2 carcinoma).tw. or ovary c56.mp.
Uterus (c54-c55)	Exp Uterine Neoplasms/ or Endometrial neoplasms/ or cancer, uter$.mp. or ((uterine or uterus or uter$) adj2 carcinoma).tw. or uterine c54.mp.
Oesophagus (c15)	exp Esophageal Neoplasms/ or ((esoph$ or oesoph$) adj3 (carcinoma or cancer)).tw.
Brain, Other CNS & Intracranial Tumours (C70-C72, C75.1-C75.3,	exp Central Nervous System Neoplasms/ or brain c70.mp. or brain d33.mp.

For peer review only - http://bmjopen.bmj.com/site/about/guidelines.xhtml

D32-D33, D35.2-D35.4, D42-D43, D44.3-D44.5)	
Liver (c22)	Exp Liver Neoplasms/ or liver cancer c22.mp.
Melanoma Skin Cancer (c43)	exp Skin neoplasms/ or exp Carcinoma, Basal cell/ or melanoma skin cancer.mp. or melanoma c43.mp. or BCC.mp.
Non-Hodgkin Lymphoma (c82-c86)	Exp Lymphoma, Non-Hodgkin/ or non-hodgkin lymphoma.mp.
Kidney (c64-66, c68)	Exp Kidney Neoplasms/ or kidney cancer.mp. or kidney c64.mp. or Carcinoma, Renal Cell/ or renal cancer.mp. or ((kidney or rena? or neph$) adj3 (carcinoma or cancer or neoplas?)).tw.
Thyroid	Exp Thyroid Neoplasms/ or Thyroid cancer.mp. or thyroid carcinoma.mp.
Leukemias	Leukemia/ or leukemia.mp. or leukaemia.mp.
**Functional health conditions**	
Chronic pain	exp Chronic Pain/
Fibromyalgia	fibromyalgia.mp. or exp FIBROMYALGIA/
Low back pain	exp Low Back Pain/ or exp Back Pain/ or chronic back pain.mp.
Interstitial cystitis	interstitial cystitis.mp. or exp Cystitis, Interstitial/
Irritable bowel syndrome	irritable bowel syndrome.mp. or exp Irritable Bowel Syndrome/
Vulvodynia	vulvodynia.mp. or exp VULVODYNIA/
Tension headache	tension headache.mp. or exp Tension -Type Headache/
**Mental health Conditions**	
Serious mental illness	((affective and non-affective) or (psychos*s or psychotic) or (bipolar* or bi-polar* or mania* or manic-depress**) or (schizophrenia* or schizo-affective or schizophrenia*) or (delusion* or paranoi*)).mp.
Common mental illness	(depress* or dysthymi* or mood disorder* or affective disorder* or distress* or neuros*).mp. or (anxiet* or obsessive compulsive disorder* or OCD* PTSD or post-traumatic stress disorder or panic* or neurosis* or neurotic* or adjustment disorder*).mp.

For peer review only - http://bmjopen.bmj.com/site/about/guidelines.xhtml

References

36. Royal college of obstetrician and gynaecologists,(2011) The investigation and treatment of couples with recurrent first-trimester and second-trimester miscarriage. Royal College of Obstetricians and Gynaecologists. http://www.rcog.org.uk

37. Legistation UK-https://www.legislation.gov.uk/ukpga/1992/29/section/1.

38. National Institute for Health and Care Excellence. NICE Guideline [NG133]: Hypertension in pregnancy: diagnosis and management. 2019. https://www.nice.org.uk/guidance/ng133/chapter/Recommendations#management-of-gestational-hypertension.

39. Royal college of obstetrician and gynaecologists,Placenta praevia, placenta accreta and vasa praevia,2018 https://www.rcog.org.uk/globalassets/documents/patients/patient-information-leaflets/pregnancy/pi-placenta-praevia-placenta-accreta-and-vasa-praevia.pdf.

40. Royal college of obstetrician and gynaecologists,The Management of Nausea andVomiting of Pregnancy and Hyperemesis Gravidarum Green-top Guideline No. 69,2016 https://www.rcog.org.uk/globalassets/documents/guidelines/green-top-guidelines/gtg69-hyperemesis.pdf.

41. BMJ-Best practice,Gestational diabetes mellitus,2021 https://bestpractice.bmj.com/topics/en-gb/665.

42. Royal college of obstetrician and gynaecologists,,Ectopic pregnancy,2016 https://www.rcog.org.uk/en/patients/patient-leaflets/ectopic-pregnancy/.

43. Royal college of obstetrician and gynaecologists,,Molar pregnancy and gestational trophoblastic disease,2019 https://www.rcog.org.uk/en/patients/patient-leaflets/gestational-trophoblastic-disease-gtd/#:~:text=Molar%20pregnancy%20is%20an%20abnormal,from%20your%20uterus%20(womb).

44. Gestational trophoblastic disease,Cancer research UK https://www.cancerresearchuk.org/about-cancer/gestational-trophoblastic-disease-gtd accessed in Feb,2022.

45. Royal college of obstetrician and gynaecologists,,Multiple pregnancy: having more than one baby,2021 https://www.rcog.org.uk/en/patients/patient-leaflets/multiple-pregnancy-having-more-than-one-baby/.

46. Royal college of obstetrician and gynaecologists,Heavy bleeding after birth (postpartum haemorrhage,,2016 https://www.rcog.org.uk/en/patients/patient-leaflets/heavy-bleeding-after-birth-postpartum-haemorrhage/.

47. World health organisation Preterm birth,2018 https://www.who.int/news-room/fact-sheets/detail/preterm-birth.

48. National maternal and Perinatal Audit-https://maternityaudit.org.uk/Audit/Charting/ClinicalByMeasure. 2017/2018.

49. World health organisation

For peer review only

Low birth weight,2019

-https://www.who.int/data/nutrition/nlis/info/low-birth-weight#:~:text=Low%20birth%20weight%20has%20been,2500%20grams%20(5.5%20pounds).

50. Royal college of obstetrician and gynaecologists,Small-for-Gestational-Age Fetus, Investigation and Management (Green-top Guideline No. 31),2013

https://www.rcog.org.uk/en/guidelines-research-services/guidelines/gtg31/.

51. National institute of health and care excellence,Depression - antenatal and postnatal,2020,https://cks.nice.org.uk/topics/depression-antenatal-postnatal/.

52. National institute of health and care excellence,Antenatal and postnatal mental health: clinical management and service guidance,2020, https://www.nice.org.uk/guidance/cg192/chapter/Introduction.

53. Royal college of obstetrician and gynaecologists Care of a third- or fourth-degre, e tear that occurred during childbirth (OASI),2019 https://www.rcpsych.ac.uk/mental-health/problems-disorders/postpartum-psychosis.

54. Pelvic girdle pain and pregnancy -<https://www.rcog.org.uk/for-the-public/browse-all-patient-information-leaflets/pelvic-girdle-pain-and-pregnancy/-assessed 27/05/2022>.

55. Royal college of obstetrician and gynaecologists ,Obstetric Cholestasis,(Green-top Guideline No. 43),2014

https://www.rcog.org.uk/globalassets/documents/patients/patient-information-leaflets/pregnancy/pi-obstetric-cholestasis.pdf

For peer review only

BMJ Open

The association of pregnancy complications/risk factors with the development of future long-term health conditions in women: overarching protocol for umbrella reviews

Journal:	*BMJ Open*
Manuscript ID	bmjopen-2022-066476.R1
Article Type:	Protocol
Date Submitted by the Author:	01-Nov-2022
Complete List of Authors:	Singh, Megha; University of Birmingham, Applied health research<br>Crowe, Francesca; University of Birmingham, Institute of Applied Health Research<br>Thangaratinam, Shakila; Queen Mary University of London, BARC (Barts Research Centre for Women's Health), Blizard Institute, Barts and the London School of Medicine and Dentistry; University of Birmingham, Institute of Metabolism and Systems Research<br>Abel, Kathryn ; Institute of Brain Behaviour and Mental Health, Medical and Human Sciences; Manchester Mental Health & Social Care Trust<br>Black, Mairead; University of Aberdeen,<br>Okoth, Kelvin; University of Birmingham Edgbaston Campus, Institute of Applied Health Research<br>Riley, Richard; Keele University,<br>Eastwood, Kelly-Ann; University Hospitals Bristol and Weston NHS Foundation Trust<br>Hope, Holly; Manchester University,<br>Wambua, Steven; University of Birmingham, Applied health research<br>Healey, Jemma ; University of Aberdeen, 6. Aberdeen Centre for Women's Health Research, School of Medicine, Medical Science and Nutrition,<br>Lee, Siang Ing; University of Birmingham Institute of Applied Health Research,<br>Phillips, Katherine; University of Birmingham, Applied health research<br>Vowles, Zoe; Guy's and St. Thomas' NHS Foundation Trust<br>Cockburn, Neil; University of Birmingham, Applied health research<br>Moss, Ngawai; Queen Mary University of London<br>Nirantharakumar, Krishnarajah; University of Birmingham, Institute of Applied Health Research
**Primary Subject Heading**:	Obstetrics and gynaecology
Secondary Subject Heading:	Sexual health, Reproductive medicine, Public health, Obstetrics and gynaecology, Mental health
Keywords:	Hypertension < CARDIOLOGY, MENTAL HEALTH, Diabetes in pregnancy < DIABETES & ENDOCRINOLOGY

Title

The association of pregnancy complications/risk factors with the development of future long-term health conditions in women: overarching protocol for umbrella reviews

Investigators and Institutional Affiliations

Dr Megha Singh,[1] Dr Francesca Crowe,[1] Professor Shakila Thangaratinam,[2, 3] Professor Kathryn Abel,[4, 5] Dr Mairead Black,[6] Dr Kelvin Okoth,[1] Professor Richard Riley,[7] Dr Kelly-Ann Eastwood,[8, 9] Dr Holly Hope,[4] Mr Steven Wambua,[1] Ms Jemma Healey,[6] Dr Siang Ing Lee,[1] Dr Katherine Phillips,[1] Ms Zoe Vowles,[10] Dr Neil Cockburn,[1] Ms Ngawai Moss,[11] Professor Krishnarajah Nirantharakumar[1]

Institutional Affiliations

1. Institute of Applied Health Research, University of Birmingham, Birmingham, UK
2. WHO Collaborating Centre for Global Women's Health, Institute of Metabolism and Systems Research, University of Birmingham, Birmingham, UK
3. Department of Obstetrics and Gynaecology, Birmingham Women's and Children's NHS Foundation Trust, Birmingham, UK
4. Centre for Women's Mental Health, Division of Psychology and Mental Health, School of Health Sciences, Faculty of Biology Medicine & Health, The University of Manchester, UK
5. Greater Manchester Mental Health NHS Foundation Trust, Manchester, UK
6. Aberdeen Centre for Women's Health Research, School of Medicine, Medical Science and Nutrition, University of Aberdeen, UK
7. Centre for Prognosis Research, School of Primary, Community and Social Care, Keele University, Staffordshire ST5 5BG
8. Centre for Public Health, Queen's University of Belfast, UK
9. St Michael's Hospital, University Hospitals Bristol NHS Foundation Trust, UK
10. Guy's and St. Thomas' NHS Foundation Trust, UK
11. Patient and public representative

Email Address of the Authors

Dr Megha Singh-m.singh.6@bham.ac.uk

Dr Francesca Crowe F.Crowe@bham.ac.uk

Professor Shakila Thangaratinam S.Thangaratinam.1@bham.ac.uk

Professor Kathryn Abel kathryn.m.abel@manchester.ac.uk

Dr Mairead Blackmairead.black@abdn.ac.uk

Dr Kelvin Okoth K.Okoth@bham.ac.uk

Professor Richard Riley r.riley@keele.ac.uk

Dr Kelly-Ann Eastwood k.eastwood@qub.ac.uk

Dr Holly Hope holly.hope@manchester.ac.uk

Mr Steven Wambua ssw107@student.bham.ac.uk

For peer review only - http://bmjopen.bmj.com/site/about/guidelines.xhtml

Ms Jemma Healey jemma.healey@abdn.ac.uk

Dr Siang Ing Lee S.I.Lee@bham.ac.uk

Dr Katherine Phillips k.phillips.1@bham.ac.uk

Ms Zoe Vowles Zoe.Vowles@gstt.nhs.uk

Dr Neil Cockburn n.cockburn@bham.ac.uk

Ms Ngawai Moss ngawai@btinternet.com

Professor Krishnarajah Nirantharakumar " K.Nirantharan@bham.ac.uk

Corresponding Author- Dr. Megha Singh, Applied health research , University of Birmingham, UK. Email-m.singh.6@bham.ac.uk

Word count – 2720

Key Words-Pregnancy complications, Cancer, Chronic pain, Hypertension, Diabetes, Autoimmune conditions

Abstract

Introduction

With good medical care, most pregnancy complications like pre-eclampsia, gestational diabetes etc. resolve after childbirth. However, pregnancy complications are known to be associated with an increased risk of new long-term health conditions for the women later in life, such as cardiovascular disease. These umbrella reviews aim to summarise systematic reviews evaluating the association between pregnancy complications and five long-term health conditions: metabolic health conditions (diabetes and hypertension), cancer, autoimmune conditions, mental health conditions, and functional disorders.

Methods and analysis

We will conduct searches in Medline, Embase, and the Cochrane database of systematic reviews without any language restrictions. We will include systematic reviews with or without meta-analyses that studied the association between pregnancy complications and the future risk of the five long-term health conditions in women. Pregnancy complications were identified from existing core outcome sets for pregnancy and consultation with experts. Two reviewers will independently screen the articles Data will be synthesised with both narrative and quantitative methods. Where a meta-analysis has been carried out, we will report the combined effect size from individual studies. For binary data, pooled odds ratios with 95% confidence intervals (CIs) will be presented. For continuous data, we will use the mean difference with 95% CIs. The findings will be presented in forest plots to assess heterogeneity. The methodological quality of the studies will be evaluated with AMSTAR 2 tool or the Cochrane risk of bias tool. The corrected covered area method will be used to assess the impact of overlap in reviews. The findings will be used to inform the design of prediction models, which will predict the risk of women developing these five health conditions following a pregnancy complication.

For peer review only - http://bmjopen.bmj.com/site/about/guidelines.xhtml

Ethics and dissemination

No ethical approvals required. Findings will be disseminated through publications in peer reviewed journals and conference presentations.

Strengths and limitations

1-These umbrella reviews will combine the results from existing systematic reviews in the topic and compile in a single document.
2-These reviews will include reviews from all languages without any restriction of language.
3- Screening of studies and quality assessment of the reviews will be independently done by both reviewers. Overlap of the reviews will be taken into account.
4-Same data extraction and quality appraisal forms will be used across the reviews and will be piloted before use.
5-There might not be an existing systematic review for a few of the rare conditions and hence will not be included in these umbrella reviews, which is a limitation.

Detailed Research Plan

Introduction

Although maternal deaths have decreased globally by 38 percent in the last two decades due to improved and accessible medical care, the occurrence of pregnancy complications has seen an increasing trend (1-4). For instance the incidence of hypertensive disorders of pregnancy increased from 16 to 18 million globally, which equates to a total of 11 % between 1990 and 2019,while prevalence of gestational diabetes has also increased during the same time period alongside rising levels of obesity and inactivity (5). Prior analyses consistently identify environmental risk factors such as air pollution, poverty, alcohol intake, diet, smoking, obesity and weight gain in pregnancy with gestational diabetes and pre-eclampsia in the mother and pre-term birth and low birth weight among the offspring. Both common (ie depression and anxiety) and serious (affective and non-affective psychotic disorders) associate with poor health in pregnancy and miscarriage, and low birth weight and . Polycystic ovarian syndrome (PCOS) increases the risk of gestational diabetes and periodontal disease has also been identified as a potential risk factor for poor preterm birth and low birth weight(6-12).

Globally 295 000 maternal deaths occurred in 2017, equivalent to 810 deaths per day, from preventable causes related to pregnancy and childbirth(13, 14). Complications arising during pregnancy and childbirth continue to be leading causes of maternal deaths, often attributed to postpartum haemorrhage or hypertensive disorders during pregnancy(15). Most pregnancy complications like hypertensive disorders and gestational diabetes resolve after birth, however, they are associated with an increased risk of complications in future pregnancies as well as long-term physical and mental health conditions. During pregnancy associated with complications or risk factors, the maternal organs undergo significant physiological changes, such as increased cardiac output and inflammatory response, which can be re-activated by age related changes in later life, resulting in development of long-term

For peer review only - http://bmjopen.bmj.com/site/about/guidelines.xhtml

health condition (16-19). Women who have experienced pre-eclampsia have an increased risk of developing type 2 diabetes along with increased risk of hypertension, ischaemic heart disease, stroke and venous thromboembolism(20-23). Depression and anxiety disorders are common after miscarriage, stillbirth and preterm births(24-26). Studies have also identified pregnancy complications such as miscarriage and pre-term birth are associated with future risk of breast cancer (27, 28).

While it is well established that gestational diabetes increases the risk of developing type 2 diabetes by tenfold, it is also associated with an increased risk of cardiovascular disease and cancer in in later life.(29, 30).Even complications often considered less serious such as hyperemesis was associated with a 70% increased risk of developing rheumatoid arthritis Relationship between miscarriage and future development of autoimmune diseases have also been identified.(31, 32).There are systematic reviews which have shown the relationship between pregnancy complications and risk factors and future development of health conditions, but there is a need of further research to generate robust evidence which can further be utilised for the betterment of maternal health post pregnancy.

We have previously published an umbrella review studying the association between pregnancy complications and cardiovascular diseases (33). In this umbrella review protocol, we describe our objectives and methods to investigate the association between pregnancy complications and five other groups of long-term health conditions, namely autoimmune conditions, cancers, functional conditions, mental health conditions and metabolic conditions(diabetes and hypertension). The umbrella reviews will aid in collating the necessary evidence to delineate the association between adverse pregnancy complications and subsequent occurrence of these health conditions. This will help develop early detection strategies and inform the prognostic factors to develop risk prediction model to predict future occurrence of these long-term health conditions.

Protocol development

This umbrella review protocol was developed following Joanna Briggs institute methodology for umbrella reviews, The reporting done through preferred reporting Items for systematic reviews and meta-analyses protocols(PRISMA-P) guidelines(appendix table 1)(34). The reporting of the reviews will be done using the preferred reporting items for systematic reviews and meta-analyses (PRISMA) guidelines. The protocol has been registered with Prospero Registration number is: CRD42022323718. In line with the Prospero registration the start date is April,2022. Any changes will be reported in detail.

Aims and objectives

Aims

These umbrella reviews will identify, appraise, and consolidate higher level evidence in the form of systematic reviews with or without meta-analyses into a single readable/usable document and provide recommendations and policy for future research. To review existing evidence from systematic reviews of pregnancy complications/risk factors associated with a woman's future risk of group of long-term health conditions.

Objectives

For peer review only - http://bmjopen.bmj.com/site/about/guidelines.xhtml

To identify and appraise higher level evidence (systematic reviews and meta-analysis) reporting on the association between pregnancy complications/ risk factors (Table 1) and future risk of

1) Autoimmune conditions
2) Cancers
3) Functional health conditions
4) Mental health conditions
5) Metabolic health conditions

If there are no systematic reviews for certain exposures and outcomes, we may consider conducting a separate systematic review that would be peer-reviewed. We will not include primary studies in our umbrella reviews, but some of the systematic reviews may include interventional studies. The health conditions included in each of the above groups are listed below(Table 2).

Research plan/Methods

The reviews aim to combine all the available evidence and present with one concrete inference. It will primarily include systematic reviews (with or without meta-analyses). Where there is no existing systematic review, the research team will undertake a scoping review to assess whether to conduct a new systematic review. The reporting of these umbrella reviews will be in accordance to the PRISMA guidelines (35).

Study design

Systematic reviews of observational and interventional studies with or without meta-analysis that have assessed the association between the pregnancy complications/risk factors and future risk of long-term health conditions in women will be considered for these umbrella review (36, 37). As the purpose of an umbrella review is to identify and synthesis evidence from systematic reviews, we will include all relevant systematic reviews. Some of these systematic reviews may include interventional studies. Any secondary analysis of looking at collected data in the interventional studies around exposure and outcome of interest will be included though it does not relate to the exposure being the intervention in the particular study.

Population

The population will include pregnant women no age restriction.

Exposures

Exposures are adverse pregnancy complications/risk factors which will be identified by scoping searches and consultation with experts. The list of pregnancy complications and risk factors identified are listed below in table 1 and the definitions are listed in appendix table 2. Depending upon outcome in question few the exposures might not be considered.

Table 1: Pregnancy complications and risk factors

For peer review only - http://bmjopen.bmj.com/site/about/guidelines.xhtml

1. Pregnancy loss
- Miscarriage/ Recurrent miscarriage/ Spontaneous pregnancy loss
- Stillbirth

2. Hypertensive disorders of pregnancy
- Gestational hypertension
- Pre-eclampsia- early or late onset
- Recurrent pre-eclampsia
- Eclampsia
- HELLP (Hemolysis, Elevated Liver enzymes and Low Platelets) syndrome

3. Placental disorders
- Placenta previa
- Placental abruption
- Placenta accreta
- Placenta percreta

4. Hyperemesis gravidarum

5. Gestational diabetes mellitus

6. Ectopic pregnancy

7. Molar pregnancy/ Choriocarcinoma

8. Multiple pregnancy/Twin-pregnancies/ Multiple gestation

9. Obstetric haemorrhage (postpartum)

10. Pre-term birth/ Recurrent pre-term birth

11. Mode of birth: Caesarean, Instrumental

12. Low birth weight
- Low birth weight
- Small for gestational age
- Intra-uterine growth retardation / intra-uterine growth restriction
- Fetal growth restriction

13. post-partum depression

14. Puerperal psychosis

15. Perineal trauma-3rd and 4th degree tear

16. Obstetric cholestasis

17. Pelvic girdle pain

For peer review only

Comparator

Pregnant women without the exposure of interest will be controls.

Outcomes

The outcome of interest is the following 5 groups of long-term health conditions.

Table 2: List of Health conditions(outcomes)

Condition	

For peer review only - http://bmjopen.bmj.com/site/about/guidelines.xhtml

**Autoimmune conditions**	Psoriasis, vitiligo, alopecia areata, systemic lupus erythematosus, psoriatic arthritis, ankylosing spondylitis, rheumatoid arthritis, sjögren's syndrome, systemic sclerosis, coeliac disease, crohn's disease, ulcerative colitis, (inflammatory bowel disease), multiple sclerosis, grave's disease, hashimoto's disease, type 1 diabetes, myasthenia gravis, addisons disease
**Cancers**	lung, breast, colorectal, cervix, cancer of unknown primary, pancreas, ovary, uterus/endometrium, brain, (other central nervous system) and intracranial tumours, liver, melanoma skin cancer, lymphoma, kidney, thyroid, leukaemia.
**Functional conditions**	fibromyalgia, chronic pain, chronic back pain, tension headache, irritable bowel syndrome, interstitial cystitis, vulvodynia,irritable bowel syndrome
**Mental health**	Serious mental illness-affective psychosis (bipolar, mania) and non-affective psychoses (schizophrenia, paranoia and psychoses nos). Common mental illness-mood disorders (depression, dysthymia) and neurotic (generalised anxiety, panic PTSD, OCD etc) disorders
**Metabolic conditions**	diabetes Type 2 and hypertension.

Search Strategy

We will conduct searches in Medline, Embase and the Cochrane database of systematic reviews from inception. We will be looking for systematic reviews with or without meta-analyses that examines the association between pregnancy complications and future risk of the long-term health condition in women. There will be no restriction of language or setting when selecting the studies.

The search strategy will be developed using subject headings and free text keywords using the concepts for pregnancy complication/ risk factors(listed in appendix table 3)and for the specific disease in the group of long-term health conditions to be studied(listed in appendix table 4). The reference list of included studies will also be searched. The search terms and detailed search strategy for Medline are provided in Appendix Table 3,4 and 5.1-5.5 respectively. These will be modified for use in other databases .A two-pronged approach will be adapted, firstly search strategy will be restricted to systematic reviews to identify which pregnancy complications/ risk factors will have evidence from systematic reviews. We will subsequently run a search strategy for primary studies if update of the review is considered. Searches will be updated periodically to identify emerging systematic reviews.

Study selection

Once the literature search is completed, reference management software will be used to manage the identified literature (e.g., Endnote, Mendeley or Reference Manager). After removing duplicate studies, two independent reviewers will conduct the title and abstract screening and ineligible studies will be excluded. Full text screening of eligible studies will be conducted by two independent reviewers and a third senior reviewer will be consulted to resolve any discrepancy. The full text will be translated if non-English studies are identified. For non-English articles, the authors will be consulted if they have expertise in the language. Followed by contacting university language departments and fellow researchers will be approached. If no one is identified with expertise in the concerned language then a professional will be hired for the translation.(38).The list of excluded studies will be

For peer review only - http://bmjopen.bmj.com/site/about/guidelines.xhtml

maintained with the reasons for exclusion documented. The details of the steps involved in study selection will be reported using the PRISMA flowchart (39).

Exclusion criteria

The following types of publications will not be included: protocols, review articles, conference abstracts, guidelines, consensus, documents or expert position papers, summaries, comments, letters, and brief reports. The reviews that include theoretical studies or text or opinion as their primary source of evidence and all narrative reviews that provided no quantitative data will not be included(34).

Quality assessment

Two reviewers will independently perform the quality assessment of the reviews using AMSTAR 2 tool.. Out of the 16 points of the AMSTAR 2 tool, one point will be awarded for each of the criteria met (40). The reviews will be rated at high, moderate, low or critically low quality. The critical domains like protocol registration, literature search detailed and including grey literature. Risk of bias of included primary studies, meta-analysis conducted appropriately, risk of bias consideration in reporting/interpretion of results and reporting of publication bias will be taken in consideration for rating the review. The reviews will not be excluded even if they are rated as being low quality. Reviews that do not mention the quality assessment of primary studies might be excluded but this will be at the reviewer's discretion. To resolve any disagreements, a third reviewer will be consulted.

Update of existing reviews

Up to 50% of systematic reviews are out of date after 5.5 years (41). The recommended methods for updating existing systematic reviews and meta-analyses will be used where a need for update is identified and the update of the review will be only be considered by authors based on these guidelines and where required(35, 42). In case the reviews need to be updated, only high and moderate quality reviews will be eligible for updates (43).

Overlap

Overlapping reviews refers to a situation where two or more reviews are examining the same research question and may include the same primary studies. The degree of overlap will be presented graphically using a citation matrix. A citation matrix is a plot of the included primary studies in the rows and systematic reviews in the columns. Overlap will be quantified by the method of corrected covered area (44). Quality rating will be used as the selection criteria where higher-quality reviews will be considered over lower-quality reviews.

Data extraction

A standardised data extraction form will be used. The data extraction form will be piloted prior to use. Data will be extracted from the final list of included studies which will then be checked by the second reviewers in case of conflict, a third reviewer will be consulted. Two reviewers will be involved in data extraction. After the first reviewer has completed the data extraction, the second reviewer will check the data extraction sheet and provide any

For peer review only - http://bmjopen.bmj.com/site/about/guidelines.xhtml

comments. Any differences will be resolved by discussion and a third reviewer will be consulted if necessary.

The data will be extracted in the following template using Microsoft Excel.

1. Study ID
2. Author/s
3. Year of publication
4. Geographical area
5. Aim of the review
6. Database searched
7. Search period
8. Population
9. Heath care setting
10. Exposures
11. Comparator
12. Outcomes
13. Covariates
14. Study design(s)
15. Definition of exposure
16. Definition of outcome
17. Data synthesis method
18. Quality assessment tool
19. Quality of the included primary studies as assessed by review authors
20. Number of studies included in qualitative analysis (narrative synthesis where meta-analysis was not done / possible by the review authors)
21. Number of meta-analyses
22. Number of studies included in each meta-analysis
23. Summary estimates of each meta-analysis and its related 95% confidence intervals
24. Author's conclusion
25. Review limitation
26. Additional comments

Recently published studies will be added into the narration of the reviews where no meta-analysis has been performed.

Data analysis

Study characteristics will be presented in a tabular format. The study will synthesise the data in both narrative and quantitative methods. Where a meta-analysis has been carried out, we will report the summary result. Where the review does not provide a summary result, we will explore the key findings and use these to inform a narrative overview of the key findings. Based on the data extracted from individual studies, systematic review or meta-analysis, data may need to be converted before pooling. For binary data, pooled odds ratios with 95% confidence intervals (CIs) will be presented. For continuous data, we will use the mean difference or standardised mean difference with 95% CIs. While analysis the results reported by the sysytematic reviews ,there may be differences in population

characteristics it might not be possible to combine all results from the included studies. If we do combine them, we may present effect sizes according to certain population characteristics such as country/region to account for differences in nutritional status. We will try to consider all heterogeneity including clinical, methodological and statistical and will ensure this is covered in the discussion section of the manuscripts of the umbrella reviews. The findings will be presented in forest plots to assess the heterogeneity of the study findings. The I^2 statistic will be used to evaluate statistical heterogeneity. All statistical analysis will be conducted using Stata and Microsoft Excel. Based on age and ethnicity subgroup analysis will be considered where appropriate. Publication bias will be assessed both quantitatively and by funnel plot(45).GRADE will be used to estimate the strength of evidence(38).

Patient Public involvement

Patient and public involvement (PPI) representatives (RP and NM) were involved in formulating the research question and study design. They have also played key role in collaboration with clinicians and researchers to identify and consider the list of pregnancy complications and health outcomes in the study. NM has been part of our regular meetings and also provided input to improve the manuscript. They will also play a key role in disseminating the results once the reviews have been undertaken.

Ethics and dissemination

No ethical approvals required. Findings will be disseminated through publications in peer reviewed journals and conferences.

Conflict of Interest

No conflict of interest reported.

Acknowledgements

Patient representatives and MuM-PreDiCT team

Authors contributions

MS was responsible for drafting the initial manuscript.KN, FC, ST, MB, KAE, HH, JH, SW, ZW, KP, NM, NC, SIL, ST,KO and KA,RR were responsible for revising the manuscript critically for important intellectual content. The authors have approved the final submitted version and are accountable for all aspects of the work.

Funding

This work was funded by the Strategic Priority Fund “Tackling multimorbidity at scale” programme [grant number-MR/W014432/1 delivered by the Medical Research Council and the National Institute for Health and Care Research in partnership with the Economic and

For peer review only - http://bmjopen.bmj.com/site/about/guidelines.xhtml

Social Research Council and in collaboration with the Engineering and Physical Sciences Research Council. The funders have no role in development of this protocol.

References

1. Kramer MS. The epidemiology of adverse pregnancy outcomes: an overview. J Nutr. 2003;133(5 Suppl 2):1592s-6s.
2. Zhang F, Dong L, Zhang CP, Li B, Wen J, Gao W, et al. Increasing prevalence of gestational diabetes mellitus in Chinese women from 1999 to 2008. Diabet Med. 2011;28(6):652-7.
3. Lavery JA, Friedman AM, Keyes KM, Wright JD, Ananth CV. Gestational diabetes in the United States: temporal changes in prevalence rates between 1979 and 2010. Bjog. 2017;124(5):804-13.
4. Bornstein E, Eliner Y, Chervenak FA, Grünebaum A. Concerning trends in maternal risk factors in the United States: 1989–2018. EClinicalMedicine. 2020;29:100657.
5. Wang, W., Xie, X., Yuan, T. et al. Epidemiological trends of maternal hypertensive disorders of pregnancy at the global, regional, and national levels: a population-based study. BMC Pregnancy Childbirth 21, 364 (2021). https://doi.org/10.1186/s12884-021-03809-2. 2021.
6. Alder J, Fink N, Bitzer J, Hösli I, Holzgreve W. Depression and anxiety during pregnancy: a risk factor for obstetric, fetal and neonatal outcome? A critical review of the literature. The Journal of Maternal-Fetal & Neonatal Medicine. 2007;20(3):189-209.
7. Ashrafi M, Sheikhan F, Arabipoor A, Hosseini R, Nourbakhsh F, Zolfaghari Z. Gestational diabetes mellitus risk factors in women with polycystic ovary syndrome (PCOS). European Journal of Obstetrics & Gynecology and Reproductive Biology. 2014;181:195-9.
8. Bai W, Li Y, Niu Y, Ding Y, Yu X, Zhu B, et al. Association between ambient air pollution and pregnancy complications: a systematic review and meta-analysis of cohort studies. Environmental research. 2020;185:109471.
9. Kęska K, Szcześniak MW, Adamus A, Czernicka M. Waterlogging-stress-responsive LncRNAs, their regulatory relationships with miRNAs and target genes in cucumber (Cucumis sativus L.). International journal of molecular sciences. 2021;22(15):8197.
10. Langley-Evans SC, Pearce J, Ellis S. Overweight, obesity and excessive weight gain in pregnancy as risk factors for adverse pregnancy outcomes: A narrative review. Journal of Human Nutrition and Dietetics. 2022;35(2):250-64.
11. Patra J, Bakker R, Irving H, Jaddoe VW, Malini S, Rehm J. Dose–response relationship between alcohol consumption before and during pregnancy and the risks of low birthweight, preterm birth and small for gestational age (SGA)—a systematic review and meta-analyses. BJOG: An International Journal of Obstetrics & Gynaecology. 2011;118(12):1411-21.
12. Scannapieco FA, Bush RB, Paju S. Periodontal disease as a risk factor for adverse pregnancy outcomes. A systematic review. Annals of Periodontology. 2003;8(1):70-8.
13. World health organisation,Maternal mortalityhttps://www.who.int/news-room/fact-sheets/detail/maternal-mortality(16/02/2022) ,(assessed Feb,2022).
14. Dr Lale Say M, Doris Chou, MD,Alison Gemmill, MPH,Özge Tunçalp, MD,Ann-Beth Moller, MSc,Jane Daniels, PhD,A Metin Gülmezoglu, MD,Marleen Temmerman, MD,Leontine Alkema, PhD. Global causes of maternal death: a WHO systematic analysis. 2014;VOLUME 2,(ISSUE 6, E323-E333).
15. World health organisation,maternal deaths fact sheet,https://www.who.int/news-room/fact-sheets/detail/maternal-mortality,22nd february,2022.
16. Sattar N, Greer IA. Pregnancy complications and maternal cardiovascular risk: opportunities for intervention and screening? BMJ. 2002 Jul 20;325(7356):157-60. doi: 10.1136/bmj.325.7356.157. PMID: 12130616; PMCID: PMC1123678.,(assessed Feb,2022). 2002.
17. Neiger R. Long-Term Effects of Pregnancy Complications on Maternal Health: A Review. Journal of Clinical Medicine. 2017;6(8):76.

For peer review only - http://bmjopen.bmj.com/site/about/guidelines.xhtml

18. Panaitescu AM PM, Ciobanu AM, Gica N, Cimpoca-Raptis BA. . Pregnancy Complications Can Foreshadow Future Disease-Long-Term Outcomes of a Complicated Pregnancy. Medicina (Kaunas). 2021;57(12):1320. Published 2021 Dec 1. doi:10.3390/medicina57121320. 2021.
19. Williams D. Pregnancy: a stress test for life. Curr Opin Obstet Gynecol. 2003 Dec;15(6):465-71. doi: 10.1097/00001703-200312000-00002. PMID: 14624211..,(assessed Feb,2022)

2003.
20. Bellamy L, Casas JP, Hingorani AD, Williams DJ. Pre-eclampsia and risk of cardiovascular disease and cancer in later life: systematic review and meta-analysis. Bmj. 2007;335(7627):974.
21. Robbins CL, Hutchings Y, Dietz PM, Kuklina EV, Callaghan WM. History of preterm birth and subsequent cardiovascular disease: a systematic review. Am J Obstet Gynecol. 2014;210(4):285-97.
22. Rich-Edwards JW. Reproductive health as a sentinel of chronic disease in women. Womens Health (Lond). 2009;5(2):101-5.
23. Wu P, Kwok CS, Haththotuwa R, Kotronias RA, Babu A, Fryer AA, et al. Pre-eclampsia is associated with a twofold increase in diabetes: a systematic review and meta-analysis. Diabetologia. 2016;59(12):2518-26.
24. Knight M BK, Tuffnell D, Shakespeare J, Kotnis R, Kenyon S, Kurinczuk JJ (Eds.) on behalf of MBRRACE-UK. Saving Lives, Improving Mothers' Care - Lessons learned to inform maternity care from the UK and Ireland Confidential

Enquiries into Maternal Deaths and Morbidity 2015-17. Oxford: National Perinatal Epidemiology Unit, University of

Oxford 2019

2015-17.
25. Brier N. Anxiety after miscarriage: a review of the empirical literature and implications for clinical practice. Birth. 2004;31(2):138-42.
26. Anderson C, Cacola P. Implications of preterm birth for maternal mental health and infant development. MCN: The American Journal of Maternal/Child Nursing. 2017;42(2):108-14.
27. Brind J, Chinchilli VM, Severs WB, Summy-Long J. Induced abortion as an independent risk factor for breast cancer: a comprehensive review and meta-analysis. J Epidemiol Community Health. 1996;50(5):481-96.
28. Wingo PA, Newsome K, Marks JS, Calle EE, Parker SL. The risk of breast cancer following spontaneous or induced abortion. Cancer Causes Control. 1997;8(1):93-108.
29. Burlina S, Dalfrà MG, Chilelli NC, Lapolla A. Gestational Diabetes Mellitus and Future Cardiovascular Risk: An Update. International Journal of Endocrinology. 2016;2016:2070926.
30. Daly B, Toulis KA, Thomas N, Gokhale K, Martin J, Webber J, et al. Increased risk of ischemic heart disease, hypertension, and type 2 diabetes in women with previous gestational diabetes mellitus, a target group in general practice for preventive interventions: a population-based cohort study. PLoS medicine. 2018;15(1):e1002488.
31. Brennan P, Bankhead C, Silman A, Symmons D, editors. Oral contraceptives and rheumatoid arthritis: results from a primary care-based incident case-control study. Seminars in arthritis and rheumatism; 1997: Elsevier.
32. Jørgensen KT, Pedersen BV, Jacobsen S, Biggar RJ, Frisch M. National cohort study of reproductive risk factors for rheumatoid arthritis in Denmark: a role for hyperemesis, gestational hypertension and pre-eclampsia? Annals of the rheumatic diseases. 2010;69(2):358-63.
33. Okoth K, Chandan JS, Marshall T, Thangaratinam S, Thomas GN, Nirantharakumar K, et al. Association between the reproductive health of young women and cardiovascular disease in later life: umbrella review. BMJ. 2020;371.
34. Institute TJB. Joanna Briggs Institute Reviewers' Manual: 2014 edition / Supplement. 2014.
35. Smith V, Devane D, Begley CM, Clarke M. Methodology in conducting a systematic review of systematic reviews of healthcare interventions. BMC medical research methodology. 2011;11(1):1-6.

For peer review only - http://bmjopen.bmj.com/site/about/guidelines.xhtml

36. Haddaway NR, Land M, Macura B. "A little learning is a dangerous thing": A call for better understanding of the term 'systematic review'. Environment International. 2017;99:356-60.
37. (ed.) GB-Z. Umbrella Reviews: Evidence Synthesis with Overviews of Reviews and Meta-Epidemiologic Studies, chapter 1 page 6.6.
38. Rockliffe L. Including non-English language articles in systematic reviews: A reflection on processes for identifying low-cost sources of translation support. Research Synthesis Methods. 2022;13(1):2-5.
39. Page MJ, McKenzie JE, Bossuyt PM, Boutron I, Hoffmann TC, Mulrow CD, et al. The PRISMA 2020 statement: an updated guideline for reporting systematic reviews. Bmj. 2021;372:n71.
40. Shea BJ, Reeves BC, Wells G, Thuku M, Hamel C, Moran J, et al. AMSTAR 2: a critical appraisal tool for systematic reviews that include randomised or non-randomised studies of healthcare interventions, or both. Bmj. 2017;358:j4008.
41. Bougioukas KI, Liakos A, Tsapas A, Ntzani E, Haidich A-B. Preferred reporting items for overviews of systematic reviews including harms checklist: a pilot tool to be used for balanced reporting of benefits and harms. Journal of clinical epidemiology. 2018;93:9-24.
42. Senn SJ. Overstating the evidence–double counting in meta-analysis and related problems. BMC Medical Research Methodology. 2009;9(1):1-7.
43. Garner P, Hopewell S, Chandler J, MacLehose H, Akl EA, Beyene J, et al. When and how to update systematic reviews: consensus and checklist. BMJ. 2016;354:i3507.
44. Lunny C, Pieper D, Thabet P, Kanji S. Managing overlap of primary study results across systematic reviews: practical considerations for authors of overviews of reviews. BMC Med Res Methodol. 2021;21(1):140.
45. Egger M, Smith GD, Schneider M, Minder C. Bias in meta-analysis detected by a simple, graphical test. Bmj. 1997;315(7109):629-34.

For peer review only

For peer review only - http://bmjopen.bmj.com/site/about/guidelines.xhtml

The association of pregnancy complications/risk factors with the development of future long-term health conditions in women: overarching protocol for umbrella reviews

Appendix/Supplementary material

- Appendix Table 1: PRISMA-P (Preferred Reporting Items for Systematic review and Meta-Analysis Protocols) 2015 checklist: recommended items to address in a systematic review protocol).
- Appendix Table 2: Definitions of pregnancy complications and risk factors
- Appendix Table 3: Search terms for pregnancy complications/risk factors (exposure)
- Appendix Table 4: Search terms for long-term health conditions (outcomes)
- Appendix Table 5.1 Search strategy- Medline Database-Autoimmune condition and pregnancy complications
- Appendix Table 5.2 search strategy- Medline database- Cancer and pregnancy complications
- Appendix Table 5.3 search strategy- Medline database- Functional conditions and pregnancy complications
- Appendix Table 5.4 search strategy- Medline database- Mental health condition and pregnancy complications
- Appendix Table 5.5 search strategy- Medline database- Metabolic conditions and pregnancy complications

Appendix Table 1: Definitions of pregnancy complications and risk factors

Pregnancy Specific factors	Definitions
Miscarriage/ Recurrent miscarriage/ Spontaneous pregnancy loss.	Spontaneous loss of a pregnancy before 24 weeks of gestation.<br>Early miscarriage, before 13 weeks of gestation.<br>Late miscarriage, between 13 and 24 weeks of gestation<br>Recurrent miscarriage is loss of three or more consecutive pregnancies before 24 weeks of gestation (36).
Stillbirth	child born after the 24th week of pregnancy who did not show any signs of life after being born(1).
Pre-eclampsia- early and late onset<br>Recurrent pre-eclampsia	New onset of hypertension (over 140 mmHg systolic or over 90 mmHg diastolic) after 20 weeks of pregnancy and the coexistence of 1 or more of the following new-onset conditions:<br>- proteinuria

For peer review only - http://bmjopen.bmj.com/site/about/guidelines.xhtml

	- other maternal organ dysfunction (renal insufficiency, liver involvement, neurological complications, haematological complications)<br>- uteroplacental dysfunction(2).
Eclampsia<br>HELLP	The new onset of seizures or coma in a pregnant woman with pre-eclampsia<br>HELLP (Hemolysis, Elevated Liver enzymes and Low Platelets) syndrome is a life-threatening pregnancy complication usually considered to be a variant of pre-eclampsia(2).
Gestational hypertension	New hypertension presenting after 20 weeks of pregnancy without significant proteinuria(2).
Placenta previa	Placenta previa is low lying placenta after 20 weeks of pregnancy(3).
Placenta accreta	Placenta accrete is where placenta is stuck to the uterine muscle(3).
Placenta percreta	Placenta percreta is a condition where placenta attaches itself and grows through the uterus and potentially to the nearby organs (such as the bladder)(3).
Placental abruption	Placental abruption is the early separation of a placenta from the lining of the uterus before completion of the second stage of labor. It is one of the causes of bleeding during the second half of pregnancy. Placental abruption is also called abruptio placentae(4, 5).
Hyperemesis gravidarum	Severe, protracted nausea and vomiting associated with weight loss of more than 5% of pre pregnancy weight, dehydration and electrolyte imbalances(6).
Gestational diabetes mellitus	hyperglycaemia in pregnancy that is above diagnostic thresholds for diabetes<br>• a fasting plasma glucose level of 5.6 mmol/litre or above or<br>• a 2-hour plasma glucose level of 7.8 mmol/litre or above(7).
Ectopic pregnancy	If the fertilised egg fails to move from the fallopian tube into the uterus, where the pregnancy grows and develops and the fertilised egg may implant and start to develop outside the uterus(8).
Molar pregnancy/ Choriocarcinoma	Abnormal cells or tumours that start in the womb from cells that would normally develop into the placenta are called Gestational trophoblastic disease (GTD)<br>Gestational trophoblastic disease (GTD) forms a group of disorders spanning the conditions of complete and partial molar pregnancies through to the malignant conditions of invasive mole, choriocarcinoma(9, 10).

For peer review only - http://bmjopen.bmj.com/site/about/guidelines.xhtml

Multiple pregnancy/Twin-pregnancies/ Multiple gestation	Term used when one is expecting two or more babies at the same time (twins, triplets or more)(11).
Obstetric / postpartum haemorrhage (PPH)	Heavy bleeding after birth postpartum haemorrhage (PPH) can be primary or secondary. Primary PPH -blood loss 500 ml (a pint) or more of blood within the first 24 hours after the birth of the baby. Secondary PPH occurs when there is abnormal or heavy vaginal bleeding between 24 hours and 12 weeks after the birth(12).
Pre-term birth/ Recurrent pre-term birth	Babies born alive before 37 weeks of pregnancy are completed. extremely preterm (less than 28 weeks),very preterm (28 to 32 weeks),moderate to late preterm (32 to 37 weeks)(13).
Mode of birth: Instrumental	Birth of a singleton/multiple baby/babies between 37+0 and 42+6 weeks of gestation, who give birth vaginally with the assistance of instruments(ventouse and forceps births)(14).
Mode of birth Caesarean	Birth of a singleton/ multiple baby/babies baby between 37+0 and 42+6 weeks of gestation, by caesarean section(elective or emergency)(14).
Low birth weight	Weight at birth of < 2500 grams (5.5 pounds)(15).
Small for gestational age	Small for gestational age Term babies with a birth weight below the 10th centile, and below the 2nd centile are provided for reference(14).
Intra-uterine growth retardation Intra uterine growth restriction	Growth restriction implies a pathological restriction of the genetic growth potential. As a result, growth restricted fetuses may manifest evidence of fetal compromise (abnormal Doppler studies, reduced liquor volume).<br>Small for gestational age Term babies with a birth weight below the 10th centile, and below the 2nd centile are provided for reference(14).
Fetal growth restriction	fetal growth restriction is defined as birth weight <3rd centile. We used severe small for gestational age at birth as a proxy for fetal growth restriction(16).
Post-partum depression	Depression like symptoms including low mood etc that starts within one or two months or several months of giving birth. Symptoms might last at least two weeks depending on the severity(17).
Puerperal psychosis	Puerperal psychosis is a severe mental illness that starts suddenly in the days, or weeks, after having a baby. Symptoms vary and can include high mood (mania), depression, confusion, hallucinations and delusions(18).
Perineal trauma-3rd and 4th degree tears	Severe perineal tears extending into the anal sphincter, anal mucosa are known as “third degree” and “fourth degree” tears also known as obstetric anal sphincter injury – OASI)(14, 19).

For peer review only - http://bmjopen.bmj.com/site/about/guidelines.xhtml

Obstetric cholestasis	Disorder that affects your liver during pregnancy causing build-up of bile acids in the body. The main symptom is itching of the skin and no rash. The symptoms get better when your baby has been born(20).
Pelvic girdle pain (PGP)	Pelvic girdle pain or symphysis pubis dysfunction (SPD).refers to pain in the front and/or the back of your pelvis .It also affect other areas such as the hips or thighs and the sacroiliac joints at the back and/or the symphysis pubis joint at the front(21).

Appendix Table 2. Search terms for pregnancy complications / risk factors (exposure)

Pregnancy complications/risk factors	pregnancy complication*.mp. or exp Pregnancy Complications/
Miscarriage/ Recurrent miscarriage/ Spontaneous pregnancy loss	exp Abortion, Spontaneous/ or ((recurrent adj3 miscarr$) or miscarr$ or early pregnancy loss$).mp.
Stillbirth	(stillbirth or still birth).mp. or exp Stillbirth/ or exp Fetal Death/ or (f?etal death$ or f?etal demise$).mp.
Hypertensive disorders of pregnancy /Gestational hypertension	Hypertension, Pregnancy-Induced/ or (gestational hypertension or (pregnancy adj3 hypertensi$)).mp.
Pre-eclampsia- early or late onset	(preeclampsia or pre-eclampsia).mp. or exp Pre-Eclampsia/
Eclampsia	exp Eclampsia/ or (eclampsia or tox?emia).mp.
HELLP (Homolysis, Elevated Liver enzymes and Low Platelets) syndrome	HELLP.mp. or exp HELLP Syndrome/
Placenta previa	placenta accreta.mp. or exp Placenta Accreta/ or placenta percreta.mp. or placenta increta.mp. or morbidly adherent placenta.mp. or abnormally invasive placenta.mp.
Placental abruption	Placenta$ abruption.mp. or exp Abruptio Placentae/
Placenta accrete and placenta percreta	placenta pr?eia.mp. or exp placenta previa/ or exp low lying placenta/
Hyperemesis gravidarum	Hyperemesis Gravidarum.mp. or Hyperemesis Gravidarum/ or morning sickness.mp. or exp Morning Sickness/
Gestational diabetes mellitus	(((pregnan$ or gestation$ or prenatal$ or antenatal$ or pre-natal$ or ante-natal$ or maternal$) adj2 diabet$) or gestational

For peer review only - http://bmjopen.bmj.com/site/about/guidelines.xhtml

	diabetes).mp. or exp Diabetes, Gestational/ or GDM.mp.
Ectopic pregnancy	ectopic pregnancy.mp. or exp Pregnancy, Ectopic/ or ((tub$$ adj3 pregnanc$) or (cornual adj3 pregnanc$) or (heterotopic adj3 pregnanc$) or (abdomin$ adj3 pregnanc$) or (extrauterine adj3 pregnanc$) or (interstitial adj3 pregnanc$) or (cervi$ adj3 pregnanc$) or (ovar$ adj3 pregnanc$) or (cesarean scar adj3 pregnanc$)).mp.
Molar pregnancy/ Choriocarcinoma	exp Gestational Trophoblastic Disease/ or gestational trophoblastic.mp. or exp Hydatidiform Mole/ or ((hydatid? adj2 mole?) or (molar adj2 pregnanc?)).mp. exp Choriocarcinoma/ or choriocarcinoma.mp.
Multiple pregnancy/Twin-pregnancies/ Multiple gestation	exp Pregnancy, Multiple/ or ((pregnanc* or gestation*) adj (twin* or triplet* or quadruplet* or quintuplet* or multiple or multi?f?et*)).mp. or (Monochorionic or dichorionic).mp.
Obstetric haemorrhage (postpartum)	exp Postpartum Hemorrhage/ or (postpartum hemorrhage or post partum hemorrhage or postpartum haemorrhage or post partum haemorrhage).ti,ab. or obstetric haemorrhage.mp.
Pre-term birth/ Recurrent pre-term birth	obstetric labor, premature.mp. or exp Obstetric Labor, Premature/ or (premature labor or premature labor or preterm labor or preterm labor or preterm birth).mp.
Mode of delivery: Caesarean or Instrumental	exp Cesarean Section, Repeat/ or cesarean.mp. or exp Cesarean Section/ or (caesarean or cesarean or caesarian or cesarian or cesarien or caesarien or c-section or c section).mp.<br><br>exp Extraction, Obstetrical/ or exp Obstetrical Forceps/ or ((operative or instrumental or assisted or forcep* or ventouse* or vacuum*) adj1 (deliver* or birth*)).mp.
Low birth weight	low birth weight.mp. or exp Infant, Low Birth Weight/ or (low birth weight* adj4 very low birth weight*).mp.

For peer review only - http://bmjopen.bmj.com/site/about/guidelines.xhtml

Small for gestational age	exp Infant, Small for Gestational Age/ or small for gestational age.mp. or (small adj3 gestational age).mp.
Intra-uterine growth retardation / intra-uterine growth restriction<br><br>Fetal growth retardation	(intra?uterine growth adj2 (restriction* or retardation)).mp. or iugr.ti,ab<br>fetal growth retardation.mp. or exp Fetal Growth Retardation/ or (fetal growth adj2 (restriction? or retardation)).mp.
postpartum depression	postpartum depression.mp. or exp Depression, Postpartum/
Puerperal psychosis	((postpartum* or post partum* or post-partum* or postnatal* or post natal* or post-natal* or perinatal* or peri natal* or peri-natal* or puerp*) and (depress* or dysthymi* or adjustment disorder* or mood disorder* or affective disorder*)).mp.<br><br>(((postpartum* or post partum* or post-partum* or postnatal* or post natal* or post-natal or perinatal* or peri natal* or peri-natal* or puerp*) and (psychos#s or psychotic)) or psychosis after childbirth).mp.
Perineal trauma(3rd and 4th degree)	(((third or fourth or 3rd or 4th) adj degree) and tear*).mp.<br><br>(((anal near adj2 sphincter) or (rectal adj mucosa) or rectum or (anal adj epithelium) or anus or (recto?vaginal adj2 fistulae) or (anorectal adj mucosa) or (anal adj skin)) and (tear* or injur* or damage* or lacerat* or rupture* or trauma)).mp.<br><br>((obstetric* and anal and sphincter and injur*) or (anal and sphincter and injur*)).mp.
Obstetric cholestasis	(exp Pregnancy/ or exp Obstetrics/ or (pregnan* or obstetric*).mp.) and (exp Cholestasis/ or exp Cholestasis, Intrahepatic/)
Pelvic girdle pain(PGP)	pelvic girdle pain.mp. or exp Pelvic Girdle Pain/ or(symphysis pubis adj3 (pain$ or dysfunction$)).mp. or Pubic Symphysis Diastasis.mp. or exp Pubic Symphysis

For peer review only

	Diastasis/ or Sacroiliac joint dysfunction.mp. or PGP.mp.

Appendix Table 3: Search terms for long-term health conditions (outcomes)

**1-Autoimmune health conditions**	
**Health conditions**	**Search terms**
Autoimmune conditions	exp Autoimmune Diseases/ or (auto?immune adj2 (disease* or condition*)).mp.
Psoriasis	exp Psoriasis/ or psoria$.mp.
Vitiligo	vitiligo.mp. or exp Vitiligo/ or (leucoderma or leukoderma).mp.
Alopecia areata	alopecia areata.mp. or exp Alopecia Areata/ or (alopecia adj totalis).mp. or (alopecia adj universalis).mp.
Systemic lupus erythematosus	exp Lupus Erythematosus, Systemic/ or systemic lupus.mp. or sle.mp.
Psoriatic arthritis	exp Arthritis, Psoriatic/ or psoriatic.mp. or (psoria? adj (arthriti? or arthropath?)).mp. or ((arthriti? or arthropath?) adj psoria?).mp.
Ankylosing spondylitis	Ankylosing spondylitis.mp. or exp Spondylitis, Ankylosing/
Rheumatoid arthritis	exp Arthritis, Rheumatoid/ or rheumatoid artheritis.mp. or ((rheumatoid or reumatoid or revmatoid or rheumatic or reumatic or revmatic or rheumat$ or reumat$ or revmarthrit$) adj3 (arthrit$ or artrit$ or diseas$ or condition$ or nodule$)).mp.
Sjögren's's syndrome	exp Sjogren's Syndrome/ or sjogren.mp.
Systemic sclerosis	Systemic Sclerosis.mp. or exp Scleroderma, Systemic/ or ((systemic adj scler?) or sclero?).mp. or crest syndrome.mp. or exp CREST Syndrome/
Coeliac disease	celiac disease.mp. or exp Celiac Disease/ or (c?eliac adj disease*).mp.
Ulcerative colitis	ulcerative colitis.mp. or exp Colitis, Ulcerative/
Crohn's disease	Crohn's disease.mp. or exp Crohn Disease/
Inflammatory bowel disease	exp Inflammatory Bowel Diseases/ or inflammatory bowel.mp.
Multiple sclerosis	multiple sclerosis.mp. or exp Multiple Sclerosis/
Graves' disease	graves disease.mp. or exp Graves Disease/ or (grave? adj6 (diseas? or thyrotoxicos? or hyperthyr?)).mp.
Hashimoto's disease	exp Thyroiditis, Autoimmune/ or or autoimmune thyroid.mp. or Hashimoto Disease/

For peer review only - http://bmjopen.bmj.com/site/about/guidelines.xhtml

Type 1 diabetes	type 1 diabetes.mp. or exp Diabetes Mellitus, Type 1/ or ("typ? 1 diabet*" or "typ? I diabet*" or "typ?1 diabet*" or "typ?I diabet*").mp. or (("insulin* depend*" or "insulin?depend*") not ("non-insulin* depend*" or "non insulindepend*")).mp. or (IDDM or T1DM or T1D).mp. or autoimmune diabetes.mp.
Myasthenia gravis	exp Myasthenia Gravis/ or mysathenia.mp.
Addison's disease	exp Addison Disease/ or addison.mp.
**2-Cancers**	
Lung (C33-34)	exp Lung Neoplasms/ or lung cancer c33 c34.mp.
Breast (C50)	exp Breast Neoplasms/ or breast cancer.mp. or breast cancer c50.mp.
Colorectal	(colorectal or colon$ or rectal or rectum or sigmoid).ti,ab,sh.) And (cancer$ or neoplasm$ or or tumor or carcinoma or adenoma or adenocarcinoma).ti,ab,sh.)
Cervix	Uterine Cervical Neoplasms/ or cancer cervix.mp. or cervical cancer.mp. or cervical neoplasms.mps or cer$ cancer.mp.
Cancer of unknown primary (c77-c80)	Neoplasms, Unknown Primary/ or cancer of unknown primary.mp.
Pancreas (c25)	exp Pancreatic Neoplasms/ or cancer pancrea$.mp. or Carcinoma, Pancreatic Ductal or pancreas$ carcinoma.mp.
Ovary (c56- c57.4)	Exp Ovarian Neoplasms/ or ((ovarian or ovary or ovar$) adj2 carcinoma).tw. or ovary c56.mp.
Uterus (c54-c55)	Exp Uterine Neoplasms/ or Endometrial neoplasms/ or cancer, uter$.mp. or ((uterine or uterus or uter$) adj2 carcinoma).tw. or uterine c54.mp.
Oesophagus (c15)	exp Esophageal Neoplasms/ or ((esoph$ or oesoph$) adj3 (carcinoma or cancer)).tw.
Brain, Other CNS & Intracranial Tumours (C70-C72, C75.1-C75.3, D32-D33, D35.2-D35.4, D42-D43, D44.3-D44.5)	exp Central Nervous System Neoplasms/ or brain c70.mp. or brain d33.mp.
Liver (c22)	Exp Liver Neoplasms/ or liver cancer c22.mp.
Melanoma Skin Cancer (c43)	exp Skin neoplasms/ or exp Carcinoma, Basal cell/ or melanoma skin cancer.mp. or melanoma c43.mp. or BCC.mp.
Non-Hodgkin Lymphoma (c82-c86)	Exp Lymphoma, Non-Hodgkin/ or non-hodgkin lymphoma.mp.
Kidney (c64-66, c68)	Exp Kidney Neoplasms/ or kidney cancer.mp. or kidney c64.mp. or Carcinoma, Renal Cell/ or

For peer review only

For peer review only - http://bmjopen.bmj.com/site/about/guidelines.xhtml

	renal cancer.mp. or ((kidney or rena? or neph$) adj3 (carcinoma or cancer or neoplas?)).tw.
Thyroid	Exp Thyroid Neoplasms/ or Thyroid cancer.mp. or thyroid carcinoma.mp.
Leukemias	Leukemia/ or leukemia.mp. or leukaemia.mp.
**3-Functional health conditions**	
Chronic pain	exp Chronic Pain/
Fibromyalgia	fibromyalgia.mp. or exp FIBROMYALGIA/
Low back pain	exp Low Back Pain/ or exp Back Pain/ or chronic back pain.mp.
Interstitial cystitis	interstitial cystitis.mp. or exp Cystitis, Interstitial/
Irritable bowel syndrome	irritable bowel syndrome.mp. or exp Irritable Bowel Syndrome/
Vulvodynia	vulvodynia.mp. or exp VULVODYNIA/
Tension headache	tension headache.mp. or exp Tension -Type Headache/
**4-Mental health condition**	
Serious mental illness	((affective and non-affective) or (psychos*s or psychotic) or (bipolar* or bi-polar* or mania* or manic-depress**) or (schizophrenia* or schizo-affective or schizophrenia*) or (delusion* or paranoi*)).mp.
Common mental illness	(depress* or dysthymi* or mood disorder* or affective disorder* or distress* or neuros*).mp. or (anxiet* or obsessive compulsive disorder* or OCD* PTSD or post-traumatic stress disorder or panic* or neurosis* or neurotic* or adjustment disorder*).mp.
**5-Metabolic health conditions**	
Types 2 diabetes	exp Diabetes Mellitus, Type 2/ or diabetes type 2.mp. or Type 2/ or (MODY or NIDDM or T2DM or T2D).mp. or (non insulin* depend* or noninsulin* depend* or noninsulin?depend* or non insulin?depend*).mp. or ((typ? 2 or typ? II or typ?2 or typ?II) adj3 diabet*).mp. or (((late or adult* or matur* or slow or stabl*) adj3 onset) and diabet*).mp.
Hypertension	exp hypertension/ or hypertens$.mp. or exp blood pressure/ or (blood pressure or bloodpressure).mp.

For peer review only

For peer review only - http://bmjopen.bmj.com/site/about/guidelines.xhtml

Appendix Table 4:PRISMA-P (Preferred Reporting Items for Systematic review and Meta-Analysis Protocols) 2015 checklist: recommended items to address in a systematic review protocol)

Section and topic	Item No	Checklist item	Paragraph and Page
ADMINISTRATIVE INFORMATION			
Title:		The association of pregnancy complications/risk factors with the development of future long-term health conditions in women: overarching protocol for umbrella reviews	
Identification	1a	Identify the report as a protocol of a systematic review	Page 4,line 141-147
Update	1b	If the protocol is for an update of a previous systematic review, identify as such	NA
Registration	2	If registered, provide the name of the registry (such as PROSPERO) and registration number	Page 4,line 145
Authors:			
Contact	3a	Provide name, institutional affiliation, e-mail address of all protocol authors; provide physical mailing address of corresponding author	Page1 and 2,line 4-46
Contributions	3b	Describe contributions of protocol authors and identify the guarantor of the review	Page 10,line 344-349
Amendments	4	If the protocol represents an amendment of a previously completed or published protocol, identify as such and list changes; otherwise, state plan for documenting important protocol amendments	NA
Support:			
Sources	5a	Indicate sources of financial or other support for the review	Page 10,line 351-356

For peer review only - http://bmjopen.bmj.com/site/about/guidelines.xhtml

Sponsor	5b	Provide name for the review funder and/or sponsor	Page 10,line 351-356
Role of sponsor or funder	5c	Describe roles of funder(s), sponsor(s), and/or institution(s), if any, in developing the protocol	Page 10,line 356
INTRODUCTION			
Rationale	6	Describe the rationale for the review in the context of what is already known	Page ,line 131-140
Objectives	7	Provide an explicit statement of the question(s) the review will address with reference to participants, interventions, comparators, and outcomes (PICO)	Page 4,line 150-154
METHODS			
Eligibility criteria	8	Specify the study characteristics (such as PICO, study design, setting, time frame) and report characteristics (such as years considered, language, publication status) to be used as criteria for eligibility for the review	Page 5,line 161-199
Information sources	9	Describe all intended information sources (such as electronic databases, contact with study authors, trial registers or other grey literature sources) with planned dates of coverage	Page 7,line 200-215
Search strategy	10	Present draft of search strategy to be used for at least one electronic database, including planned limits, such that it could be repeated	Appendix Table 5.1-5.5
Study records:			
Data management	11a	Describe the mechanism(s) that will be used to manage records and data throughout the review	Page 7,line 217-218
Selection process	11b	State the process that will be used for selecting studies (such as two independent reviewers) through each phase of the review (that is, screening, eligibility and inclusion in meta-analysis)	Page 7,line 217-226
Data collection process	11c	Describe planned method of extracting data from reports (such as piloting forms, done independently, in duplicate), any processes for obtaining and confirming data from investigators	Page 8 and 9,line 265-304

For peer review only - http://bmjopen.bmj.com/site/about/guidelines.xhtml

Data items	12	List and define all variables for which data will be sought (such as PICO items, funding sources), any pre-planned data assumptions and simplifications	Appendix table 1
Outcomes and prioritization	13	List and define all outcomes for which data will be sought, including prioritization of main and additional outcomes, with rationale	Table 2
Risk of bias in individual studies	14	Describe anticipated methods for assessing risk of bias of individual studies, including whether this will be done at the outcome or study level, or both; state how this information will be used in data synthesis	Page 8,line 238-248
Data synthesis	15a	Describe criteria under which study data will be quantitatively synthesised	Page 9and 10,line 306-325
	15b	If data are appropriate for quantitative synthesis, describe planned summary measures, methods of handling data and methods of combining data from studies, including any planned exploration of consistency (such as I^2, Kendall's τ)	Page 9and 10,line 306-325
	15c	Describe any proposed additional analyses (such as sensitivity or subgroup analyses, meta-regression)	Page 10,line 323
	15d	If quantitative synthesis is not appropriate, describe the type of summary planned	Page 10,line 308
Meta-bias(es)	16	Specify any planned assessment of meta-bias(es) (such as publication bias across studies, selective reporting within studies)	Page 10,line325
Confidence in cumulative evidence	17	Describe how the strength of the body of evidence will be assessed (such as GRADE)	Page 10,line326

For peer review only

Appendix Table 4.1 Search strategy- Medline database-Autoimmune condition and pregnancy complications

1	exp Autoimmune Diseases/
2	(auto?immune adj2 (disease* or condition*)).mp.
3	exp Psoriasis/
4	psoria$.mp.
5	exp Vitiligo/

For peer review only - http://bmjopen.bmj.com/site/about/guidelines.xhtml

6	vitiligo.mp.
7	(leucoderma or leukoderma).mp.
8	exp Alopecia Areata/
9	alopecia areata.mp.
10	((alopecia adj totalis) or (alopecia adj universalis)).mp.
11	exp Lupus Erythematosus, Systemic/
12	(systemic lupus or sle).mp.
13	arthritis psoriatic.mp. or exp Arthritis, Psoriatic/
14	Ankylosing spondylitis.mp. or exp Spondylitis, Ankylosing/
15	Spondylarthritis/
16	(axial adj2 spondylarthritis).tw.
17	(axial adj2 spa).tw.
18	(ankylos$ or spondyl$).tw.
19	(bekhterev$ or bechterew$).tw.
20	(Marie adj struempell$).tw.
21	(AS or axSPA).tw.
22	Sacroiliitis/
23	((axial or spin$ or peripheral or vertebral or enthesitis) adj3 (joint$ or spondyloarthritis or arthritis or ankylosing)).tw.
24	exp Arthritis, Rheumatoid/
25	((rheumatoid or reumatoid or revmatoid or rheumatic or reumatic or revmatic or rheumat$ or reumat$ or revmarthrit$) adj3 (arthrit$ or artrit$ or diseas$ or condition$ or nodule$)).mp.
26	sjogren syndrome.mp. or exp Sjogren's Syndrome/
27	exp Celiac Disease/
28	(c?eliac adj disease*).mp.
29	inflammatory bowel disease.mp. or exp Inflammatory Bowel Diseases/
30	ulcerative colitis.mp. or exp Colitis, Ulcerative/
31	Crohn's disease.mp. or exp Crohn Disease/
32	multiple sclerosis.mp. or exp Multiple Sclerosis/
33	Systemic Sclerosis.mp.
34	exp Scleroderma, Systemic/
35	((systemic adj scler?) or sclero?).mp.
36	crest syndrome.mp. or exp CREST Syndrome/
37	graves disease.mp. or exp Graves Disease/
38	(grave? adj3 (diseas? or thyrotoxicos? or hyperthyr?)).mp.
39	exp Thyroiditis, Autoimmune/
40	autoimmune thyroid.mp.
41	Hashimoto Disease.mp.
42	exp Hashimoto Disease/
43	(thyroid adj autoantibodies).mp. [mp=title, book title, abstract, original title, name of substance word, subject heading word, floating sub-heading word, keyword heading word, organism supplementary concept word, protocol supplementary concept word, rare disease supplementary concept word, unique identifier, synonyms]
44	thyroid.mp. and exp autoantibodies/
45	myasthenia gravis.mp. or exp Myasthenia Gravis/
46	addison disease.mp. or exp Addison Disease/
47	type 1 diabetes.mp. or exp Diabetes Mellitus, Type 1/ or ("typ? 1 diabet*" or "typ? I diabet*" or "typ?1 diabet*" or "typ?I diabet*").mp. or (("insulin* depend*" or

For peer review only

	"insulin?depend*") not ("non-insulin* depend*" or "non insulindepend*")).mp. or (IDDM or T1DM or T1D).mp. or autoimmune diabetes.mp.
48	exp Pregnancy Complications/
49	(pregnancy adj complication?).mp.
50	exp Abortion, Spontaneous/
51	((recurrent adj2 miscarr?) or miscarr?).mp.
52	(early adj3 pregnancy loss?).mp.
53	miscarriage.mp.
54	exp Stillbirth/
55	(stillbirth or (still adj birth)).mp.
56	exp Fetal Death/
57	((f?etal adj death$) or (f?etal adj demise$)).mp.
58	exp Hypertension, Pregnancy-Induced/
59	(gestational hypertension or (pregnancy adj3 hypertensi$)).mp.
60	(preeclampsia or pre-eclampsia).mp.
61	exp Pre-Eclampsia/
62	(eclampsia or tox?emia).mp.
63	exp Eclampsia/
64	hellp.mp. or exp HELLP Syndrome/
65	exp Placenta Accreta/
66	(placenta adj accreta).mp.
67	placenta percreta.mp.
68	placenta increta.mp.
69	(morbidly adj3 adherent placenta).mp.
70	abnormally invasive placenta.mp.
71	exp Abruptio Placentae/
72	Placenta? abruption.mp.
73	exp Placenta Previa/
74	Placenta pr?evia.mp.
75	low lying placenta.mp.
76	exp Hyperemesis Gravidarum/
77	Hyperemesis Gravidarum.mp.
78	exp Morning Sickness/
79	morning sickness.mp.
80	exp Diabetes, Gestational/
81	GDM.mp.
82	(((pregnan$ or gestation$ or prenatal$ or antenatal$ or pre-natal$ or ante-natal$ or maternal$) adj2 diabet$) or gestational diabetes).mp.
83	((tub$$ adj3 pregnanc$) or (cornual adj3 pregnanc$) or (heterotopic adj3 pregnanc$) or (abdomin$ adj3 pregnanc$) or (extrauterine adj3 pregnanc$) or (interstitial adj3 pregnanc$) or (cervi$ adj3 pregnanc$) or (ovar$ adj3 pregnanc$) or (cesarean scar adj3 pregnanc$)).mp.
84	exp Pregnancy, Ectopic/
85	ectopic pregnancy.mp.
86	exp Gestational Trophoblastic Disease/
87	gestational trophoblastic.mp.
88	exp Hydatidiform Mole/
89	((hydatid? adj2 mole?) or (molar adj2 pregnanc?)).mp.
90	exp Choriocarcinoma/
91	choriocarcinoma.mp.

For peer review only - http://bmjopen.bmj.com/site/about/guidelines.xhtml

92	exp Pregnancy, Multiple/
93	((pregnanc* or gestation*) adj (twin* or triplet* or quadruplet* or quintuplet* or multiple or multi?f?et*)).mp.
94	(Monochorionic or dichorionic).mp.
95	exp Postpartum Hemorrhage/
96	(postpartum hemorrhage or post partum hemorrhage or postpartum haemorrhage or post partum haemorrhage).mp.
97	obstetric haemorrhage.mp.
98	obstetric labor, premature.mp.
99	exp Obstetric Labor, Premature/
100	(premature labor or premature labour or preterm labor or preterm labour or preterm birth).mp.
101	exp Cesarean Section/
102	exp Cesarean Section, Repeat/
103	(caesarean or cesarean or caesarian or cesarian or cesarien or caesarien or c-section or c section).mp.
104	cesarean.mp.
105	exp Extraction, Obstetrical/
106	exp Obstetrical Forceps/
107	((operative or instrumental or assisted or forcep* or ventouse* or vacuum*) adj1 (deliver* or birth*)).mp.
108	exp Infant, Low Birth Weight/
109	low birth weight.mp.
110	(low birth weight* adj4 very low birth weight*).mp.
111	exp Infant, Small for Gestational Age/
112	(small adj3 gestational age).mp.
113	(intra?uterine growth adj2 (restriction* or retardation)).mp.
114	iugr.ti,ab.
115	exp Fetal Growth Retardation/
116	(fetal growth adj2 (restriction? or retardation)).mp.
117	exp Depression, Postpartum/
118	postpartum depression.mp.
119	((postpartum* or post partum* or post-partum* or postnatal* or post natal* or post-natal* or perinatal* or peri natal* or peri-natal* or puerp*) and (depress* or dysthymi* or adjustment disorder* or mood disorder* or affective disorder*)).mp.
120	((postpartum* or post partum* or post-partum* or postnatal* or post natal* or post-natal or perinatal* or peri natal* or peri-natal* or puerp*) and (psychos#s or psychotic)).mp.
121	(psychosis adj3 after childbirth).mp.
122	(((third or fourth or 3rd or 4th) adj degree) and tear*).mp.
123	(((anal near adj2 sphincter) or (rectal adj mucosa) or rectum or (anal adj epithelium) or anus or (recto?vaginal adj2 fistulae) or (anorectal adj mucosa) or (anal adj skin)) and (tear* or injur* or damage* or lacerat* or rupture* or trauma)).mp.
124	((obstetric* and anal and sphincter and injur*) or (anal and sphincter and injur*)).mp.
125	(exp Pregnancy/ or exp Obstetrics/ or (pregnan* or obstetric*).mp.) and (exp Cholestasis/ or exp Cholestasis, Intrahepatic/)
126	pelvic girdle pain.mp. or exp Pelvic Girdle Pain/
127	(symphysis pubis adj3 (pain$ or dysfunction$)).mp.
128	Pubic Symphysis Diastasis.mp. or exp Pubic Symphysis Diastasis/
129	Sacroiliac joint dysfunction.mp.
130	PGP.mp.

For peer review only - http://bmjopen.bmj.com/site/about/guidelines.xhtml

131	1 or 2 or 3 or 4 or 5 or 6 or 7 or 8 or 9 or 10 or 11 or 12 or 13 or 14 or 15 or 16 or 17 or 18 or 19 or 20 or 21 or 22 or 23 or 24 or 25 or 26 or 27 or 28 or 29 or 30 or 31 or 32 or 33 or 34 or 35 or 36 or 37 or 39 or 40 or 41 or 42 or 43 or 44 or 45 or 46 or 47
132	48 or 49 or 50 or 51 or 52 or 53 or 54 or 55 or 56 or 57 or 58 or 59 or 60 or 61 or 62 or 63 or 64 or 65 or 66 or 67 or 68 or 69 or 70 or 71 or 72 or 73 or 74 or 75 or 76 or 77 or 78 or 79 or 80 or 81 or 82 or 83 or 84 or 85 or 86 or 87 or 88 or 89 or 90 or 91 or 92 or 93 or 94 or 95 or 96 or 97 or 98 or 99 or 100 or 101 or 102 or 103 or 104 or 105 or 106 or 107 or 108 or 109 or 110 or 111 or 112 or 113 or 114 or 115 or 116 or 117 or 118 or 119 or 120 or 121 or 122 or 123 or 124 or 125 or 126 or 127 or 128 or 129 or 130
133	131 and 132
134	(systematic$ adj2 (review$ or overview)).ti,ab.
135	(systematic$ adj5 review$).tw,sh.
136	meta-analysis.mp. or exp meta-analysis/
137	134 or 135 or 136
138	133 and 137

Appendix Table 5.2 search strategy- Medline database- Cancer and pregnancy complications

1.	exp Abortion, Spontaneous/
2.	(stillbirth or still birth).mp.
3.	(recurrent adj3 miscarr$).mp. [mp=title, abstract, original title, name of substance word, subject heading word, floating sub-heading word, keyword heading word, organism supplementary concept word, protocol supplementary concept word, rare disease supplementary concept word, unique identifier, synonyms]
4.	(miscarr$ or early pregnancy loss$).mp. [mp=title, abstract, original title, name of substance word, subject heading word, floating sub-heading word, keyword heading word, organism supplementary concept word, protocol supplementary concept word, rare disease supplementary concept word, unique identifier, synonyms]
5.	exp Stillbirth/
6.	exp Fetal Death/
7.	(f?etal death$ or f?etal demise$).mp.
8.	Hypertension, Pregnancy-Induced/
9.	(gestational hypertension or (pregnancy adj3 hypertensi$)).mp.
10.	(preeclampsia or pre-eclampsia).mp.
11.	exp Pre-Eclampsia/
12.	exp Eclampsia/
13.	(eclampsia or tox?emia).mp.
14.	HELLP.mp.
15.	exp HELLP Syndrome/
16.	placenta accreta.mp.
17.	exp Placenta Accreta/
18.	placenta percreta.mp.
19.	placenta increta.mp.
20.	morbidly adherent placenta.mp.

For peer review only - http://bmjopen.bmj.com/site/about/guidelines.xhtml

21.	abnormally invasive placenta.mp.
22.	Placenta$ abruption.mp.
23.	exp Abruptio Placentae/
24.	placenta pr?eia.mp.
25.	exp placenta previa/
26.	low lying placenta.mp.
27.	Hyperemesis Gravidarum.mp.
28.	Hyperemesis Gravidarum/
29.	morning sickness.mp.
30.	exp Morning Sickness/
31.	(((pregnan$ or gestation$ or prenatal$ or antenatal$ or pre-natal$ or ante-natal$ or maternal$) adj2 diabet$) or gestational diabetes).mp.
32.	exp Diabetes, Gestational/
33.	GDM.mp.
34.	ectopic pregnancy.mp.
35.	exp Pregnancy, Ectopic/
36.	((tub$$ adj3 pregnanc$) or (cornual adj3 pregnanc$) or (heterotopic adj3 pregnanc$) or (abdomin$ adj3 pregnanc$) or (extrauterine adj3 pregnanc$) or (interstitial adj3 pregnanc$) or (cervi$ adj3 pregnanc$) or (ovar$ adj3 pregnanc$) or (cesarean scar adj3 pregnanc$)).mp.
37.	exp Gestational Trophoblastic Disease/
38.	gestational trophoblastic.mp.
39.	exp Hydatidiform Mole/
40.	((hydatid? adj2 mole?) or (molar adj2 pregnanc?)).mp.
41.	exp Choriocarcinoma/ or choriocarcinoma.mp.
42.	exp Pregnancy, Multiple/
43.	exp Pregnancy, Multiple/ or ((pregnanc* or gestation*) adj (twin* or triplet* or quadruplet* or quintuplet* or multiple or multi?f?et*)).mp.
44.	(Monochorionic or dichorionic).mp.
45.	exp Postpartum Hemorrhage/
46.	(postpartum hemorrhage or post partum hemorrhage or postpartum haemorrhage or post partum haemorrhage).ti,ab.
47.	obstetric haemorrhage.mp.
48.	obstetric labor, premature.mp.
49.	exp Obstetric Labor, Premature/
50.	(premature labor or premature labour or preterm labor or preterm labour or preterm birth).mp.
51.	exp Cesarean Section, Repeat/ or cesarean.mp.
52.	exp Cesarean Section/
53.	(caesarean or cesarean or caesarian or cesarian or cesarien or caesarien or c-section or c section).mp.
54.	exp Extraction, Obstetrical/
55.	exp Obstetrical Forceps/
56.	((operative or instrumental or assisted or forcep* or ventouse* or vacuum*) adj1 (deliver* or birth*)).mp.
57.	low birth weight.mp.

For peer review only - http://bmjopen.bmj.com/site/about/guidelines.xhtml

58.	exp Infant, Low Birth Weight/
59.	(low birth weight* adj4 very low birth weight*).mp.
60.	exp Infant, Small for Gestational Age/
61.	small for gestational age.mp.
62.	(small adj3 gestational age).mp.
63.	(intra?uterine growth adj2 (restriction* or retardation)).mp.
64.	iugr.ti,ab.
65.	fetal growth retardation.mp.
66.	exp Fetal Growth Retardation/
67.	foetal growth retardation.mp.
68.	(fetal growth adj2 (restriction? or retardation)).mp.
69.	(foetal growth adj2 (restriction? or retardation)).mp.
70.	((postpartum* or post partum* or post-partum* or postnatal* or post natal* or post-natal* or perinatal* or peri natal* or peri-natal* or puerp*) and (depress* or dysthymi* or adjustment disorder* or mood disorder* or affective disorder*)).mp.
71.	(((third or fourth or 3rd or 4th) adj degree) and tear*).mp.
72.	(((anal near adj2 sphincter) or (rectal adj mucosa) or rectum or (anal adj epithelium) or anus or (recto?vaginal adj2 fistulae) or (anorectal adj mucosa) or (anal adj skin)) and (tear* or injur* or damage* or lacerat* or rupture* or trauma)).mp.
73.	((obstetric* and anal and sphincter and injur*) or (anal and sphincter and injur*)).mp.
74.	(exp Pregnancy/ or exp Obstetrics/ or (pregnan* or obstetric*).mp.) and (exp Cholestasis/ or exp Cholestasis, Intrahepatic/)
75	pregnancy complication*.mp.
76	exp Pregnancy Complications/
77	exp Lung Neoplasms/
78.	lung cancer c33 c34.mp.
79	exp Breast Neoplasms/
80	breast cancer.mp.
81	breast cancer c50.mp.
82	Uterine Cervical Neoplasms/
83	cancer cervix.mp.
84	cervical cancer.mp.
85	cervical neoplasms.mp.
86	cer$ cancer.mp.
87	Neoplasms, Unknown Primary/
88.	cancer of unknown primary.mp.
89	exp Pancreatic Neoplasms/
90	cancer pancrea$.mp.
91	Carcinoma, Pancreatic Ductal/
92	pancreas$ carcinoma.mp.
93	exp Ovarian Neoplasms/
94	((ovarian or ovary or ovar$) adj2 carcinoma).tw.
95	ovary c56.mp.
96	exp Uterine Neoplasms/

For peer review only - http://bmjopen.bmj.com/site/about/guidelines.xhtml

97	Endometrial neoplasms/
98	cancer, uter$.mp.
99	((uterine or uterus or uter$) adj2 carcinoma).tw.
100	uterine c54.mp.
101	exp Esophageal Neoplasms/
102	((esoph$ or oesoph$) adj3 (carcinoma or cancer)).tw.
103	exp Central Nervous System Neoplasms/
104	brain c70.mp.
105	brain d33.mp.
106	exp Liver Neoplasms/
107	liver cancer c22.mp.
108	exp Skin neoplasms/
109	melanoma skin cancer.mp.
110	melanoma c43.mp.
111	exp Kidney Neoplasms/
112	kidney cancer.mp.
113	kidney c64.mp.
114	Carcinoma, Renal Cell/
115	renal cancer.mp.
116	((kidney or rena? or neph$) adj3 (carcinoma or cancer or neoplas?)).tw.
117	((kidney or rena? or neph$) adj3 (carcinoma or cancer or neoplas?)).tw.
118	exp Thyroid Neoplasms/
119	Thyroid cancer.mp.
120	thyroid carcinoma.mp.
121	Leukemia/
122	leukemia.mp.
123	leukaemia.mp.
124	exp Colorectal Neoplasms/
125	colorectal cancer.mp.
126.	exp Lymphoma/
127	lymphoma.mp.
128	1 or 2 or 3 or 4 or 5 or 6 or 7or 8 or 9 or 10 or 11 or 12 or 13 or 14 or 15 or 16 or 17 or 18 or 19 or 20 or 21 or 22 or 23 or 24 or 25 or 26 or 27 or 28 or 29 or 30 or 31 or 32 or 33 or 34 or 35 or 36 or 37 or 38 or 39 or 40 or 41 or 42 or 43 or 44 or 45 or 46 or 47 or 48 or 49 or 50 or 51 or 52 or 53 or 54 or 55 or 56 or 57 or 58 or 59 or 60 or 61 or 62 or 63 or 64 or 65 or 66 or 67 or 68 or 69 or 71 or 72 or 73 or 74 or 75 or 76
129	77 or 78 or 79 or 80 or 81 or 82 or 83 or 84 or 85 or 86 or 87 or 88 or 89 or 90 or 91 or 92 or 93 or 94 or 95 or 96 or 97 or 98 or 99 or 100 or 101 or 102 or 103 or 104 or 105 or 106 or 107 or 108 or 109 or 110 or 111 or 112 or 113 or 114 or 115 or 116 or 117 or 118 or 119 or 120 0r 121 or 122 or 123 or 124 or 125 or 126 or 127

For peer review only - http://bmjopen.bmj.com/site/about/guidelines.xhtml

130	128 or 129
131	(systematic$ adj2 (review$ or overview)).ti,ab.
132	(systematic$ adj5 review$).tw,sh.
133	meta-analysis.mp. or exp meta-analysis/
134	131 or 132 or 133
135	130 and 134

Appendix Table 5.3 Search strategy- Medline database- Funtional conditions and pregnancy complications

For peer review only

1.	exp Abortion, Spontaneous/
2.	(stillbirth or still birth).mp.
3.	(recurrent adj3 miscarr$).mp. [mp=title, abstract, original title, name of substance word, subject heading word, floating sub-heading word, keyword heading word, organism supplementary concept word, protocol supplementary concept word, rare disease supplementary concept word, unique identifier, synonyms]
4.	(miscarr$ or early pregnancy loss$).mp. [mp=title, abstract, original title, name of substance word, subject heading word, floating sub-heading word, keyword heading word, organism supplementary concept word, protocol supplementary concept word, rare disease supplementary concept word, unique identifier, synonyms]
5.	exp Stillbirth/
6.	exp Fetal Death/
7.	(f?etal death$ or f?etal demise$).mp.
8.	Hypertension, Pregnancy-Induced/
9.	(gestational hypertension or (pregnancy adj3 hypertensi$)).mp.
10.	(preeclampsia or pre-eclampsia).mp.
11.	exp Pre-Eclampsia/
12.	exp Eclampsia/
13.	(eclampsia or tox?emia).mp.
14.	HELLP.mp.
15.	exp HELLP Syndrome/
16.	placenta accreta.mp.
17.	exp Placenta Accreta/
18.	placenta percreta.mp.
19.	placenta increta.mp.
20.	morbidly adherent placenta.mp.
21.	abnormally invasive placenta.mp.
22.	Placenta$ abruption.mp.
23.	exp Abruptio Placentae/
24.	placenta pr?eia.mp.
25.	exp placenta previa/
26.	low lying placenta.mp.
27.	Hyperemesis Gravidarum.mp.

For peer review only - http://bmjopen.bmj.com/site/about/guidelines.xhtml

28.	Hyperemesis Gravidarum/
29.	morning sickness.mp.
30.	exp Morning Sickness/
31.	(((pregnan$ or gestation$ or prenatal$ or antenatal$ or pre-natal$ or ante-natal$ or maternal$) adj2 diabet$) or gestational diabetes).mp.
32.	exp Diabetes, Gestational/
33.	GDM.mp.
34.	ectopic pregnancy.mp.
35.	exp Pregnancy, Ectopic/
36.	((tub$$ adj3 pregnanc$) or (cornual adj3 pregnanc$) or (heterotopic adj3 pregnanc$) or (abdomin$ adj3 pregnanc$) or (extrauterine adj3 pregnanc$) or (interstitial adj3 pregnanc$) or (cervi$ adj3 pregnanc$) or (ovar$ adj3 pregnanc$) or (cesarean scar adj3 pregnanc$)).mp.
37.	exp Gestational Trophoblastic Disease/
38.	gestational trophoblastic.mp.
39.	exp Hydatidiform Mole/
40.	((hydatid? adj2 mole?) or (molar adj2 pregnanc?)).mp.
41.	exp Choriocarcinoma/ or choriocarcinoma.mp.
42.	exp Pregnancy, Multiple/
43.	exp Pregnancy, Multiple/ or ((pregnanc* or gestation*) adj (twin* or triplet* or quadruplet* or quintuplet* or multiple or multi?f?et*)).mp.
44.	(Monochorionic or dichorionic).mp.
45.	exp Postpartum Hemorrhage/
46.	(postpartum hemorrhage or post partum hemorrhage or postpartum haemorrhage or post partum haemorrhage).ti,ab.
47.	obstetric haemorrhage.mp.
48.	obstetric labor, premature.mp.
49.	exp Obstetric Labor, Premature/
50.	(premature labor or premature labour or preterm labor or preterm labour or preterm birth).mp.
51.	exp Cesarean Section, Repeat/ or cesarean.mp.
52.	exp Cesarean Section/
53.	(caesarean or cesarean or caesarian or cesarian or cesarien or caesarien or c-section or c section).mp.
54.	exp Extraction, Obstetrical/
55.	exp Obstetrical Forceps/
56.	((operative or instrumental or assisted or forcep* or ventouse* or vacuum*) adj1 (deliver* or birth*)).mp.
57.	low birth weight.mp.
58.	exp Infant, Low Birth Weight/
59.	(low birth weight* adj4 very low birth weight*).mp.
60.	exp Infant, Small for Gestational Age/
61.	small for gestational age.mp.
62.	(small adj3 gestational age).mp.
63.	(intra?uterine growth adj2 (restriction* or retardation)).mp.
64.	iugr.ti,ab.

For peer review only - http://bmjopen.bmj.com/site/about/guidelines.xhtml

65.	fetal growth retardation.mp.
66.	exp Fetal Growth Retardation/
67.	foetal growth retardation.mp.
68.	(fetal growth adj2 (restriction? or retardation)).mp.
69.	(foetal growth adj2 (restriction? or retardation)).mp.
70.	((postpartum* or post partum* or post-partum* or postnatal* or post natal* or post-natal* or perinatal* or peri natal* or peri-natal* or puerp*) and (depress* or dysthymi* or adjustment disorder* or mood disorder* or affective disorder*)).mp.
71.	(((third or fourth or 3rd or 4th) adj degree) and tear*).mp.
72.	(((anal near adj2 sphincter) or (rectal adj mucosa) or rectum or (anal adj epithelium) or anus or (recto?vaginal adj2 fistulae) or (anorectal adj mucosa) or (anal adj skin)) and (tear* or injur* or damage* or lacerat* or rupture* or trauma)).mp.
73.	((obstetric* and anal and sphincter and injur*) or (anal and sphincter and injur*)).mp.
74.	(exp Pregnancy/ or exp Obstetrics/ or (pregnan* or obstetric*).mp.) and (exp Cholestasis/ or exp Cholestasis, Intrahepatic/)
75	pregnancy complication*.mp.
76	exp Pregnancy Complications/
77	exp FIBROMYALGIA/
78.	exp Low Back Pain/
79	exp Back Pain/
80	chronic back pain.mp.
81	interstitial cystitis.mp.
82	exp Cystitis, Interstitial/
83	irritable bowel syndrome.mp.
84	exp Irritable Bowel Syndrome/
85	vulvodynia.mp.
86	exp VULVODYNIA/
87	tension headache.mp.
88.	exp Tension -Type Headache/
89	1 or 2 or 3 or 4 or 5 or 6 or 7or 8 or 9 or 10 or 11 or 12 or 13 or 14 or 15 or 16 or 17 or 18 or 19 or 20 or 21 or 22 or 23 or 24 or 25 or 26 or 27 or 28 or 29 or 30 or 31 or 32 or 33 or 34 or 35 or 36 or 37 or 38 or 39 or 40 or 41 or 42 or 43 or 44 or 45 or 46 or 47 or 48 or 49 or 50 or 51 or 52 or 53 or 54 or 55 or 56 or 57 or 58 or 59 or 60 or 61 or 62 or 63 or 64 or 65 or 66 or 67 or 68 or 69 or 71 or 72 or 73 or 74 or 75 or 76
90	77 or 78 or 79 or 80 or 81 or 82 or 83 or 84 or 85 or 86 or 87 or 88
91	89 and 90
92	(systematic$ adj2 (review$ or overview)).ti,ab.
93	(systematic$ adj5 review$).tw,sh.
94	meta-analysis.mp. or exp meta-analysis/
95	92 or 93 or 94
96	91 and 95

For peer review only

Appendix Table 5.4 search strategy-mental health conditions and pregnancy complications

	Search term
1	pregnancy complication*.mp. or exp Pregnancy Complications/
2	exp Abortion, Spontaneous/ or ((recurrent adj3 miscarr$) or miscarr$ or early pregnancy loss$).mp.
3	(stillbirth or still birth).mp. or exp Stillbirth/ or exp Fetal Death/ or (f?etal death$ or f?etal demise$).mp.
4	Hypertension, Pregnancy-Induced/ or (gestational hypertension or (pregnancy adj3 hypertensi$)).mp.
5	(preeclampsia or pre-eclampsia).mp. or exp Pre-Eclampsia/
6	exp Eclampsia/ or (eclampsia or tox?emia).mp.
7	HELLP.mp. or exp HELLP Syndrome/
8	placenta accreta.mp. or exp Placenta Accreta/ or placenta percreta.mp. or placenta increta.mp. or morbidly adherent placenta.mp. or abnormally invasive placenta.mp.
9	Placenta$ abruption.mp. or exp Abruptio Placentae/
10	placenta pr?eia.mp. or exp placenta previa/ or exp low lying placenta/
11	Hyperemesis Gravidarum.mp. or Hyperemesis Gravidarum/ or morning sickness.mp. or exp Morning Sickness/

For peer review only

For peer review only - http://bmjopen.bmj.com/site/about/guidelines.xhtml

12	(((pregnan$ or gestation$ or prenatal$ or antenatal$ or pre-natal$ or ante-natal$ or maternal$) adj2 diabet$) or gestational diabetes).mp. or exp Diabetes, Gestational/ or GDM.mp.
13	ectopic pregnancy.mp. or exp Pregnancy, Ectopic/ or ((tub$$ adj3 pregnanc$) or (cornual adj3 pregnanc$) or (heterotopic adj3 pregnanc$) or (abdomin$ adj3 pregnanc$) or (extrauterine adj3 pregnanc$) or (interstitial adj3 pregnanc$) or (cervi$ adj3 pregnanc$) or (ovar$ adj3 pregnanc$) or (cesarean scar adj3 pregnanc$)).mp.
14	exp Gestational Trophoblastic Disease/ or gestational trophoblastic.mp. or exp Hydatidiform Mole/ or ((hydatid? adj2 mole?) or (molar adj2 pregnanc?)).mp. or exp Choriocarcinoma/ or choriocarcinoma.mp.
15	exp Pregnancy, Multiple/ or ((pregnanc* or gestation*) adj (twin* or triplet* or quadruplet* or quintuplet* or multiple or multi?f?et*)).mp. or (Monochorionic or dichorionic).mp.
16	exp Postpartum Hemorrhage/ or (postpartum hemorrhage or post partum hemorrhage or postpartum haemorrhage or post partum haemorrhage).ti,ab. or obstetric haemorrhage.mp.
17	obstetric labor, premature.mp. or exp Obstetric Labor, Premature/ or (premature labor or premature labour or preterm labor or preterm labour or preterm birth).mp.
18	exp Cesarean Section, Repeat/ or cesarean.mp. or exp Cesarean Section/ or (caesarean or cesarean or caesarian or cesarian or cesarien or caesarien or c-section or c section).mp. or exp Extraction, Obstetrical/ or exp Obstetrical Forceps/ or ((operative or instrumental or assisted or forcep* or ventouse* or vacuum*) adj1 (deliver* or birth*)).mp.
19	low birth weight.mp. or exp Infant, Low Birth Weight/ or (low birth weight* adj4 very low birth weight*).mp.
20	exp Infant, Small for Gestational Age/ or small for gestational age.mp. or (small adj3 gestational age).mp.

For peer review only - http://bmjopen.bmj.com/site/about/guidelines.xhtml

For peer review only

21	(intra?uterine growth adj2 (restriction* or retardation)).mp. or iugr.ti,ab. or fetal growth retardation.mp. or exp Fetal Growth Retardation/ or (fetal growth adj2 (restriction? or retardation)).mp.
22	((postpartum* or post partum* or post-partum* or postnatal* or post natal* or post-natal* or perinatal* or peri natal* or peri-natal* or puerp*) and (depress* or dysthymi* or adjustment disorder* or mood disorder* or affective disorder*)).mp.
23	(((postpartum* or post partum* or post-partum* or postnatal* or post natal* or post-natal or perinatal* or peri natal* or peri-natal* or puerp*) and (psychos#s or psychotic)) or psychosis after childbirth).mp.
24	((((third or fourth or 3rd or 4th) adj degree) and tear*) or (((anal near adj2 sphincter) or (rectal adj mucosa) or rectum or (anal adj epithelium) or anus or (recto?vaginal adj2 fistulae) or (anorectal adj mucosa) or (anal adj skin)) and (tear* or injur* or damage* or lacerat* or rupture* or trauma)) or ((obstetric* and anal and sphincter and injur*) or (anal and sphincter and injur*))).mp.
25	(exp Pregnancy/ or exp Obstetrics/ or (pregnan* or obstetric*).mp.) and (exp Cholestasis/ or exp Cholestasis, Intrahepatic/)
26	pelvic girdle pain.mp. or exp Pelvic Girdle Pain/ or (symphysis pubis adj3 (pain$ or dysfunction$)).mp. or Pubic Symphysis Diastasis.mp. or exp Pubic Symphysis Diastasis/ or Sacroiliac joint dysfunction.mp. or PGP.mp.
27	1 or 2 or 3 or 4 or 5 or 6 or 7 or 8 or 9 or 10 or 11 or 12 or 13 or 14 or 15 or 16 or 17 or 18 or 19 or 20 or 21 or 22 or 23 or 24 or 25 or 26
28	((affective and non-affective) or (psychos*s or psychotic) or (bipolar* or bi-polar* or mania* or manic-depress**) or (schizophrenia* or schizo-affective or schizophrenia*) or (delusion* or paranoi*)).mp.
29	(depress* or dysthymi* or mood disorder* or affective disorder* or distress* or neuros*).mp.
30	(anxiet* or obsessive compulsive disorder* or OCD* PTSD or post-traumatic stress disorder or panic* or neurosis* or neurotic* or adjustment disorder*).mp.

For peer review only - http://bmjopen.bmj.com/site/about/guidelines.xhtml

31	(systematic review or meta-analysis).mp.
32	29 or 30
33	27 and 28
34	27 and 28 and 31
35	27 and 32
36	27 and 31 and 32

Appendix Table 5.5 Search strategy- Medline database-metabolic conditions and pregnancy complications

	Search terms
1	pregnancy complication*.mp. or exp Pregnancy Complications/
2	exp Abortion, Spontaneous/ or ((recurrent adj3 miscarr$) or miscarr$ or early pregnancy loss$).mp.
3	(stillbirth or still birth).mp. or exp Stillbirth/ or exp Fetal Death/ or (f?etal death$ or f?etal demise$).mp.
4	Hypertension, Pregnancy-Induced/ or (gestational hypertension or (pregnancy adj3 hypertensi$)).mp.
5	(preeclampsia or pre-eclampsia).mp. or exp Pre-Eclampsia/
6	exp Eclampsia/ or (eclampsia or tox?emia).mp.
7	HELLP.mp. or exp HELLP Syndrome/
8	placenta accreta.mp. or exp Placenta Accreta/ or placenta percreta.mp. or placenta increta.mp. or morbidly adherent placenta.mp. or abnormally invasive placenta.mp.
9	Placenta$ abruption.mp. or exp Abruptio Placentae/

For peer review only - http://bmjopen.bmj.com/site/about/guidelines.xhtml

For peer review only

10	placenta pr?eia.mp. or exp placenta previa/ or exp low lying placenta/
11	Hyperemesis Gravidarum.mp. or Hyperemesis Gravidarum/ or morning sickness.mp. or exp Morning Sickness/
12	(((pregnan$ or gestation$ or prenatal$ or antenatal$ or pre-natal$ or ante-natal$ or maternal$) adj2 diabet$) or gestational diabetes).mp. or exp Diabetes, Gestational/ or GDM.mp.
13	ectopic pregnancy.mp. or exp Pregnancy, Ectopic/ or ((tub$$ adj3 pregnanc$) or (cornual adj3 pregnanc$) or (heterotopic adj3 pregnanc$) or (abdomin$ adj3 pregnanc$) or (extrauterine adj3 pregnanc$) or (interstitial adj3 pregnanc$) or (cervi$ adj3 pregnanc$) or (ovar$ adj3 pregnanc$) or (cesarean scar adj3 pregnanc$)).mp.
14	exp Gestational Trophoblastic Disease/ or gestational trophoblastic.mp. or exp Hydatidiform Mole/ or ((hydatid? adj2 mole?) or (molar adj2 pregnanc?)).mp.
15	exp Choriocarcinoma/ or choriocarcinoma.mp.
16	exp Pregnancy, Multiple/ or ((pregnanc* or gestation*) adj (twin* or triplet* or quadruplet* or quintuplet* or multiple or multi?f?et*)).mp. or (Monochorionic or dichorionic).mp.
17	exp Postpartum Hemorrhage/ or (postpartum hemorrhage or post partum hemorrhage or postpartum haemorrhage or post partum haemorrhage).ti,ab. or obstetric haemorrhage.mp.
18	obstetric labor, premature.mp. or exp Obstetric Labor, Premature/ or (premature labor or premature labour or preterm labor or preterm labour or preterm birth).mp.
19	exp Cesarean Section, Repeat/ or cesarean.mp. or exp Cesarean Section/ or (caesarean or cesarean or caesarian or cesarian or cesarien or caesarien or c-section or c section).mp.
20	exp Extraction, Obstetrical/ or exp Obstetrical Forceps/ or ((operative or instrumental or assisted or forcep* or ventouse* or vacuum*) adj1 (deliver* or birth*)).mp.
21	low birth weight.mp. or exp Infant, Low Birth Weight/ or (low birth weight* adj4 very low birth weight*).mp.

For peer review only

For peer review only - http://bmjopen.bmj.com/site/about/guidelines.xhtml

22	exp Infant, Small for Gestational Age/ or small for gestational age.mp. or (small adj3 gestational age).mp.
23	(intra?uterine growth adj2 (restriction* or retardation)).mp. or iugr.ti,ab.
24	fetal growth retardation.mp. or exp Fetal Growth Retardation/ or (fetal growth adj2 (restriction? or retardation)).mp.
25	postpartum depression.mp. or exp Depression, Postpartum/
26	((postpartum* or post partum* or post-partum* or postnatal* or post natal* or post-natal* or perinatal* or peri natal* or peri-natal* or puerp*) and (depress* or dysthymi* or adjustment disorder* or mood disorder* or affective disorder*)).mp.
27	(((postpartum* or post partum* or post-partum* or postnatal* or post natal* or post-natal or perinatal* or peri natal* or peri-natal* or puerp*) and (psychos#s or psychotic)) or psychosis after childbirth).mp.
28	(((third or fourth or 3rd or 4th) adj degree) and tear*).mp.
29	(((anal near adj2 sphincter) or (rectal adj mucosa) or rectum or (anal adj epithelium) or anus or (recto?vaginal adj2 fistulae) or (anorectal adj mucosa) or (anal adj skin)) and (tear* or injur* or damage* or lacerat* or rupture* or trauma)).mp. [mp=title, book title, abstract, original title, name of substance word, subject heading word, floating sub-heading word, keyword heading word, organism supplementary concept word, protocol supplementary concept word, rare disease supplementary concept word, unique identifier, synonyms]
30	((obstetric* and anal and sphincter and injur*) or (anal and sphincter and injur*)).mp.
31	(exp Pregnancy/ or exp Obstetrics/ or (pregnan* or obstetric*).mp.) and (exp Cholestasis/ or exp Cholestasis, Intrahepatic/)
32	1 or 2 or 3 or 4 or 5 or 6 or 7 or 8 or 9 or 10 or 11 or 12 or 13 or 14 or 15 or 16 or 17 or 18 or 19 or 20 or 21 or 22 or 23 or 24 or 25 or 26 or 27 or 28 or 29 or 30 or 31

For peer review only

For peer review only - http://bmjopen.bmj.com/site/about/guidelines.xhtml

33	exp Diabetes Mellitus, Type 2/ or diabetes type 2.mp. or Type 2/ or (MODY or NIDDM or T2DM or T2D).mp. or (non insulin* depend* or noninsulin* depend* or noninsulin?depend* or non insulin?depend*).mp. or ((typ? 2 or typ? II or typ?2 or typ?II) adj3 diabet*).mp. or (((late or adult* or matur* or slow or stabl*) adj3 onset) and diabet*).mp.
34	exp hypertension/ or hypertens$.mp. or exp blood pressure/ or (blood pressure or bloodpressure).mp.
35	33 or 34
36	systematic review.mp.
37	(systematic$ adj2 (review$ or overview)).ti,ab.
38	(systematic$ adj5 review$).tw,sh.
39	meta-analysis.mp. or exp meta-analysis/
40	36 or 37 or 38 or 39
41	32 and 35 and 40

References

1. Legistation UK-https://www.legislation.gov.uk/ukpga/1992/29/section/1.
2. National Institute for Health and Care Excellence. NICE Guideline [NG133]: Hypertension in pregnancy: diagnosis and management. 2019. https://www.nice.org.uk/guidance/ng133/chapter/Recommendations#management-of-gestational-hypertension.
3. Royal college of obstetrician and gynaecologists,Placenta praevia, placenta accreta and

vasa praevia,2018

https://www.rcog.org.uk/globalassets/documents/patients/patient-information-leaflets/pregnancy/pi-placenta-praevia-placenta-accreta-and-vasa-praevia.pdf.
4. Workalemahu T, Enquobahrie DA, Gelaye B, Thornton TA, Tekola-Ayele F, Sanchez SE, et al. Abruptio placentae risk and genetic variations in mitochondrial biogenesis and oxidative phosphorylation: replication of a candidate gene association study. American journal of obstetrics and gynecology. 2018;219(6):617. e1-. e17.
5. Martinelli KG, Garcia ÉM, Santos Neto ETd, Gama SGNd. Advanced maternal age and its association with placenta praevia and placental abruption: a meta-analysis. Cadernos de saude publica. 2018;34.

For peer review only - http://bmjopen.bmj.com/site/about/guidelines.xhtml

6. Royal college of obstetrician and gynaecologists,The Management of Nausea andVomiting of Pregnancy and Hyperemesis Gravidarum

Green-top Guideline No. 69,2016

https://www.rcog.org.uk/globalassets/documents/guidelines/green-top-guidelines/gtg69-hyperemesis.pdf.

7. BMJ-Best practice,Gestational diabetes mellitus,2021

https://bestpractice.bmj.com/topics/en-gb/665.

8. Royal college of obstetrician and gynaecologists,,Ectopic pregnancy,2016

https://www.rcog.org.uk/en/patients/patient-leaflets/ectopic-pregnancy/.

9. Royal college of obstetrician and gynaecologists,,Molar pregnancy and gestational trophoblastic disease,2019

https://www.rcog.org.uk/en/patients/patient-leaflets/gestational-trophoblastic-disease-gtd/#:~:text=Molar%20pregnancy%20is%20an%20abnormal,from%20your%20uterus%20(womb).

10. Gestational trophoblastic disease,Cancer research UK https://www.cancerresearchuk.org/about-cancer/gestational-trophoblastic-disease-gtd accessed in Feb,2022.

11. Royal college of obstetrician and gynaecologists,,Multiple pregnancy: having more than one baby,2021

https://www.rcog.org.uk/en/patients/patient-leaflets/multiple-pregnancy-having-more-than-one-baby/.

12. Royal college of obstetrician and gynaecologists,Heavy bleeding after birth (postpartum haemorrhage,,2016

https://www.rcog.org.uk/en/patients/patient-leaflets/heavy-bleeding-after-birth-postpartum-haemorrhage/.

13. World health organisation

Preterm birth,2018

https://www.who.int/news-room/fact-sheets/detail/preterm-birth.

14. National maternal and Perinatal Audit-https://maternityaudit.org.uk/Audit/Charting/ClinicalByMeasure. 2017/2018.

15. World health organisation

Low birth weight,2019

-https://www.who.int/data/nutrition/nlis/info/low-birth-weight#:~:text=Low%20birth%20weight%20has%20been,2500%20grams%20(5.5%20pounds).

16. Royal college of obstetrician and gynaecologists,Small-for-Gestational-Age Fetus, Investigation and Management (Green-top Guideline No. 31),2013

https://www.rcog.org.uk/en/guidelines-research-services/guidelines/gtg31/.

17. National institute of health and care excellence,Depression - antenatal and postnatal,2020,https://cks.nice.org.uk/topics/depression-antenatal-postnatal/.

18. National institute of health and care excellence,Antenatal and postnatal mental health: clinical management and service guidance,2020, https://www.nice.org.uk/guidance/cg192/chapter/Introduction.

19. Royal college of obstetrician and gynaecologists Care of a third- or fourth-degre, e tear that occurred during childbirth (OASI),2019 https://www.rcpsych.ac.uk/mental-health/problems-disorders/postpartum-psychosis.

For peer review only - http://bmjopen.bmj.com/site/about/guidelines.xhtml

20. Royal college of obstetrician and gynaecologists ,Obstetric Cholestasis,(Green-top Guideline No. 43),2014

https://www.rcog.org.uk/globalassets/documents/patients/patient-information-leaflets/pregnancy/pi-obstetric-cholestasis.pdf

21. Pelvic girdle pain and pregnancy -<https://www.rcog.org.uk/for-the-public/browse-all-patient-information-leaflets/pelvic-girdle-pain-and-pregnancy/-assessed 27/05/2022>.

For peer review only

The association of pregnancy complications/risk factors with the development of future long-term health conditions in women: overarching protocol for umbrella reviews

Journal:	*BMJ Open*
Manuscript ID	bmjopen-2022-066476.R2
Article Type:	Protocol
Date Submitted by the Author:	08-Dec-2022
Complete List of Authors:	Singh, Megha; University of Birmingham, Applied health research<br>Crowe, Francesca; University of Birmingham, Institute of Applied Health Research<br>Thangaratinam, Shakila; Queen Mary University of London, BARC (Barts Research Centre for Women's Health), Blizard Institute, Barts and the London School of Medicine and Dentistry; University of Birmingham, Institute of Metabolism and Systems Research<br>Abel, Kathryn ; Institute of Brain Behaviour and Mental Health, Medical and Human Sciences; Manchester Mental Health & Social Care Trust<br>Black, Mairead; University of Aberdeen,<br>Okoth, Kelvin; University of Birmingham Edgbaston Campus, Institute of Applied Health Research<br>Riley, Richard; Keele University,<br>Eastwood, Kelly-Ann; University Hospitals Bristol and Weston NHS Foundation Trust<br>Hope, Holly; Manchester University,<br>Wambua, Steven; University of Birmingham, Applied health research<br>Healey, Jemma ; University of Aberdeen, 6. Aberdeen Centre for Women's Health Research, School of Medicine, Medical Science and Nutrition,<br>Lee, Siang Ing; University of Birmingham Institute of Applied Health Research,<br>Phillips, Katherine; University of Birmingham, Applied health research<br>Vowles, Zoe; Guy's and St. Thomas' NHS Foundation Trust<br>Cockburn, Neil; University of Birmingham, Applied health research<br>Moss, Ngawai; Queen Mary University of London<br>Nirantharakumar, Krishnarajah; University of Birmingham, Institute of Applied Health Research
**Primary Subject Heading**:	Obstetrics and gynaecology
Secondary Subject Heading:	Sexual health, Reproductive medicine, Public health, Obstetrics and gynaecology, Mental health
Keywords:	Hypertension < CARDIOLOGY, MENTAL HEALTH, Diabetes in pregnancy < DIABETES & ENDOCRINOLOGY

Title

The association of pregnancy complications/risk factors with the development of future long-term health conditions in women: overarching protocol for umbrella reviews

Investigators and Institutional Affiliations

Dr. Megha Singh[1], Dr. Francesca Crowe[1], Professor Shakila Thangaratinam[2, 3], Professor Kathryn Abel[4, 5], Dr Mairead Black[6], Dr. Kelvin Okoth[1], Professor Richard Riley[1], Dr. Kelly-Ann Eastwood[8, 9], Dr. Holly Hope[4], Mr. Steven Wambua[1], Ms. Jemma Healey[6], Dr. Siang Ing Lee,[1] Dr. Katherine Phillips[1], Ms. Zoe Vowles[10],Dr. Neil Cockburn[1] , Ms. Ngawai Moss[11] ,Professor Krishnarajah Nirantharakumar[1]

Institutional Affiliations

1. Institute of Applied Health Research, University of Birmingham, Birmingham, UK
2. WHO Collaborating Centre for Global Women's Health, Institute of Metabolism and Systems Research, University of Birmingham, Birmingham, UK
3. Department of Obstetrics and Gynaecology, Birmingham Women's and Children's NHS Foundation Trust, Birmingham, UK
4. Centre for Women's Mental Health, Division of Psychology and Mental Health, School of Health Sciences, Faculty of Biology Medicine & Health, The University of Manchester, UK
5. Greater Manchester Mental Health NHS Foundation Trust, Manchester, UK
6. Aberdeen Centre for Women's Health Research, School of Medicine, Medical Science and Nutrition, University of Aberdeen, UK
7. Centre for Prognosis Research, School of Primary, Community and Social Care, Keele University, Staffordshire ST5 5BG
8. Centre for Public Health, Queen's University of Belfast, UK
9. St Michael's Hospital, University Hospitals Bristol NHS Foundation Trust, UK
10. Guy's and St. Thomas' NHS Foundation Trust, UK
11. Patient and public representative

Email Address of the Authors

Dr. Megha Singh-m.singh.6@bham.ac.uk

Dr. Francesca Crowe f.crowe@bham.ac.uk

Professor Shakila Thangaratinam s.thangaratinam.1@bham.ac.uk

Professor Kathryn Abel kathryn.m.abel@manchester.ac.uk

Dr. Mairead Black mairead.black@abdn.ac.uk

Dr. Kelvin Okoth k.okoth@bham.ac.uk

Professor Richard Riley r.riley@keele.ac.uk

Dr. Kelly-Ann Eastwood k.eastwood@qub.ac.uk

Dr. Holly Hope holly.hope@manchester.ac.uk

For peer review only - http://bmjopen.bmj.com/site/about/guidelines.xhtml

Mr. Steven Wambua ssw107@student.bham.ac.uk

Ms. Jemma Healey jemma.healey@abdn.ac.uk

Dr. Siang Ing Lee s.i.Lee@bham.ac.uk

Dr. Katherine Phillips k.phillips.1@bham.ac.uk

Ms. Zoe Vowles zoe.vowles@gstt.nhs.uk

Dr. Neil Cockburn n.cockburn@bham.ac.uk

Ms. Ngawai Moss ngawai@btinternet.com

Professor Krishnarajah Nirantharakumar k.nirantharan@bham.ac.uk

Corresponding Author- Dr. Megha Singh, Applied health research, University of Birmingham, UK. Email-m.singh.6@bham.ac.uk

Word count – 2720

Key Words-Pregnancy complications, cancer, chronic pain, hypertension, diabetes, autoimmune conditions.

Abstract

Introduction

With good medical care, most pregnancy complications like pre-eclampsia, gestational diabetes etc. resolve after childbirth. However, pregnancy complications are known to be associated with an increased risk of new long-term health conditions for women later in life, such as cardiovascular disease. These umbrella reviews aim to summarise systematic reviews evaluating the association between pregnancy complications and five groups of long-term health conditions: autoimmune conditions, cancers, functional disorders, mental health conditions, and metabolic health conditions (diabetes and hypertension).

Methods and analysis

We will conduct searches in Medline, Embase, and the Cochrane database of systematic reviews without any language restrictions. We will include systematic reviews with or without meta-analyses that studied the association between pregnancy complications and the future risk of the five groups of long-term health conditions in women. Pregnancy complications were identified from existing core outcome sets for pregnancy and after consultation with experts. Two reviewers will independently screen the articles. Data will be synthesised with both narrative and quantitative methods. Where a meta-analysis has been carried out, we will report the combined effect size from individual studies. For binary data, pooled odds ratios with 95% confidence intervals (CIs) will be presented. For continuous data, we will use the mean difference with 95% CIs. The findings will be presented in forest plots to assess heterogeneity. The methodological quality of the studies will be evaluated with the AMSTAR 2 tool or the Cochrane risk of bias tool. The corrected covered area method will be used to assess the impact of overlap in reviews. The findings will be used to

For peer review only - http://bmjopen.bmj.com/site/about/guidelines.xhtml

inform the design of prediction models, which will predict the risk of women developing these five group of health conditions following a pregnancy complication.

Ethics and dissemination

No ethical approvals required. Findings will be disseminated through publications in peer reviewed journals and conference presentations.

Strengths and limitations

1-These umbrella reviews will combine the results from existing systematic reviews in the topic and compile in a single document.

2-These reviews will include reviews from all languages without any restriction of language.

3- Screening of studies and quality assessment of the reviews will be independently done by both reviewers. Overlap of the reviews will be taken into account.

4-The same data extraction and quality appraisal forms will be used across the reviews and will be piloted before use.

5-A limitation is that there might not be an existing systematic review for a few of the rare conditions and so these will not be included in the umbrella review.

Detailed Research Plan

Introduction

Although maternal deaths have decreased globally by 38 percent in the last two decades due to improved and more accessible medical care, the occurrence of pregnancy complications has seen an increasing trend (1-4). For instance, worldwide incidence of hypertensive disorders of pregnancy increased by 11% over the past 20 years going from 16 to 18 million. The prevalence of gestational diabetes has also increased during the same time period alongside rising levels of obesity and inactivity (5). Prior analyses have consistently identified environmental risk factors such as air pollution, poverty, alcohol intake, diet, smoking, obesity and weight gain in pregnancy with gestational diabetes and pre-eclampsia in the mother and pre-term birth and low birth weight among the offspring. Both common mental health disorders (i.e. depression and anxiety) and serious mental health disorders (affective and non-affective psychotic disorders) are associated with poor health in pregnancy. Polycystic ovarian syndrome (PCOS) increases the risk of gestational diabetes and periodontal disease has also been identified as a potential risk factor for preterm birth and low birth weight (6-12).

Globally, there were 295 000 maternal deaths in 2017 that were attributed to preventable causes related to pregnancy and childbirth, equivalent to 810 deaths per day (13, 14). Complications arising during pregnancy and childbirth continue to be leading causes of maternal deaths, the most common of which are postpartum haemorrhage or hypertensive disorders during pregnancy (15). Most pregnancy complications like hypertensive disorders and gestational diabetes resolve after birth; however, they are associated with an increased risk of complications in future pregnancies as well as long-term physical and mental health

For peer review only - http://bmjopen.bmj.com/site/about/guidelines.xhtml

conditions. During pregnancy, the maternal organs undergo significant physiological changes, such as increased cardiac output and inflammatory response due to the complications or risk factors, which can be re-activated by age related changes in later life, resulting in development of long-term health conditions (16-19). Women who have experienced pre-eclampsia have an increased risk of developing type 2 diabetes along with increased risk of hypertension, ischaemic heart disease, stroke and venous thromboembolism (20-23). Depression and anxiety disorders are common after miscarriage, stillbirth and preterm births (24-26). Studies have also identified pregnancy complications such as miscarriage and pre-term birth are associated with future risk of breast cancer (27, 28).

While it is well established that gestational diabetes increases the risk of developing type 2 diabetes by tenfold, it is also associated with an increased risk of cardiovascular disease and cancer in later life (29, 30). Even complications often considered less serious such as hyperemesis gravidarum were associated with a 70% increased risk of developing rheumatoid arthritis.A relationship between miscarriage and future development of autoimmune diseases have also been identified (31, 32).There are systematic reviews which have shown the relationship between pregnancy complications and risk factors and future development of health conditions, but there is a need for further research to generate robust evidence which can further be utilised for the betterment of maternal health post pregnancy.

We have previously published an umbrella review studying the association between pregnancy complications and cardiovascular diseases (33). In this umbrella review protocol, we describe our objectives and methods to investigate the associations of pregnancy complications and five other groups of long-term health conditions, namely autoimmune conditions, cancers, functional conditions, mental health conditions and metabolic conditions (diabetes and hypertension). The umbrella reviews will aid in collating the necessary evidence to delineate the association between adverse pregnancy complications and subsequent occurrence of these health conditions. This will help develop early detection strategies and inform the prognostic factors that will be used to develop risk prediction models to predict future occurrence of these long-term health conditions.

Protocol development

This umbrella review protocol was developed following Joanna Briggs Institute (JBI) methodology for umbrella reviews (34). The reporting was done using the preferred reporting items for systematic reviews and meta-analyses protocols (PRISMA-P) guidelines (appendix table 1) (35). The protocol has been registered with Prospero (registration number CRD42022323718). In line with the Prospero registration the start date was April, 2022. Any deviations from the protocol will be reported in detail in the umbrella reviews.

Aims and objectives

Aims

These umbrella reviews will identify, appraise, and consolidate higher level evidence in the form of systematic reviews with or without meta-analyses into a single readable/usable document and provide recommendations for future research.

Objectives

For peer review only - http://bmjopen.bmj.com/site/about/guidelines.xhtml

To identify and appraise higher level evidence (systematic reviews and meta-analyses) reporting the associations between pregnancy complications/ risk factors (Table 1) and future risk of

1) Autoimmune conditions
2) Cancers
3) Functional health conditions
4) Mental health conditions
5) Metabolic health conditions

If there are no systematic reviews for specific exposures and outcomes, we may consider conducting a separate systematic review that would be subject to peer-review. We will not include primary studies in our umbrella reviews. The health conditions included in each of the above groups are listed below (Table 2).

Research plan/Methods

Each umbrella review aims to identify, appraise, combine and synthesise all the available evidence for each outcome. The umbrella reviews will primarily include systematic reviews with or without meta-analyses (36, 37). Where there is no existing systematic review, the research team will undertake a scoping review to assess whether to conduct a new separate systematic review. The reporting of the reviews will be done using the preferred reporting items for systematic reviews and meta-analyses (PRISMA) guidelines (38).

Study design

Systematic reviews of observational and interventional studies with or without meta-analysis that have assessed the association between the pregnancy complications/risk factors and future risk of long-term health conditions in women will be considered for these umbrella reviews. As the purpose of an umbrella review is to identify and synthesis evidence from systematic reviews, we will include all relevant systematic reviews (39, 40). Some of these systematic reviews may include interventional studies. Any secondary analysis of data collected in intervention studies around exposure and outcome of interest will be included although the exposure does not have to be the intervention in the intervention study.

Population

The population will include pregnant women no age restriction.

Exposures

The exposure of interest are adverse pregnancy complications/risk factors which will be identified by scoping searches and consultation with experts. The list of pregnancy complications and risk factors identified are listed below in table 1 and the definitions are listed in appendix table 2. Depending upon the outcome in question, a few of the exposures might not be considered.

Table 1: Pregnancy complications and risk factors

For peer review only - http://bmjopen.bmj.com/site/about/guidelines.xhtml

1. Pregnancy loss
- Miscarriage/ Recurrent miscarriage/ Spontaneous pregnancy loss
- Stillbirth
2. Hypertensive disorders of pregnancy
- Gestational hypertension
- Pre-eclampsia- early or late onset
- Recurrent pre-eclampsia
- Eclampsia
- HELLP (Hemolysis, Elevated Liver enzymes and Low Platelets) syndrome
3. Placental disorders
- Placenta previa
- Placental abruption
- Placenta accreta
- Placenta percreta
4. Hyperemesis gravidarum
5. Gestational diabetes mellitus
6. Ectopic pregnancy
7. Molar pregnancy/ Choriocarcinoma
8. Multiple pregnancy/Twin-pregnancies/ Multiple gestation
9. Obstetric haemorrhage (postpartum)
10. Pre-term birth/ Recurrent pre-term birth
11. Mode of birth: Caesarean, Instrumental
12. Low birth weight
- Low birth weight
- Small for gestational age
- Intra-uterine growth retardation / intra-uterine growth restriction
- Fetal growth restriction
13. post-partum depression
14. Puerperal psychosis
15. Perineal trauma-3rd and 4th degree tear
16. Obstetric cholestasis
17. Pelvic girdle pain

For peer review only

Comparator

Pregnant women without the exposure of interest will be the comparator group.

Outcomes

The outcomes of interest are the following five groups of long-term health conditions described in table 2.

Table 2: List of Health conditions (outcomes)

Conditions	

For peer review only - http://bmjopen.bmj.com/site/about/guidelines.xhtml

**Autoimmune conditions**	Psoriasis, vitiligo, alopecia areata, systemic lupus erythematosus, psoriatic arthritis, ankylosing spondylitis, rheumatoid arthritis, Sjögren's syndrome, systemic sclerosis, coeliac disease, Crohn's disease, ulcerative colitis, (inflammatory bowel disease), multiple sclerosis, grave's disease, Hashimoto's disease, type 1 diabetes mellitus, myasthenia gravis, Addison's disease
**Cancers**	Lung, breast, colorectal, cervix, cancer of unknown primary, pancreas, ovary, uterus/endometrium, brain, (other central nervous system) and intracranial tumours, liver, melanoma skin cancer, lymphoma, kidney, thyroid, leukaemia.
**Functional conditions**	Fibromyalgia, chronic pain, chronic back pain, tension headache, irritable bowel syndrome, interstitial cystitis, vulvodynia, irritable bowel syndrome
**Mental health**	Serious mental illness-affective psychosis (bipolar, mania) and non-affective psychoses (schizophrenia, paranoia and psychoses nos.)<br>Common mental illness-mood disorders (depression, dysthymia) and neurotic (generalised anxiety, panic, post-traumatic stress disorder (PTSD), obsessive compulsive disorder (OCD) etc.) disorders
**Metabolic conditions**	Type 2 diabetes mellitus and hypertension.

Search Strategy

We will conduct searches in Medline, Embase and the Cochrane database of systematic reviews from inception. We will be looking for systematic reviews with or without meta-analysis that examine the associations between pregnancy complications and future risk of the long-term health condition in women. There will be no restriction of language or setting when selecting the studies.

The search strategy will be developed using subject headings and free text keywords using the concepts for pregnancy complication/risk factors (listed in appendix table 3) and for the specific disease in the group of long-term health conditions to be studied (listed in appendix table 4). The reference list of included studies will also be searched. The search terms and detailed search strategy for Medline are provided in Appendix Table 3, 4 and 5.1-5.5, respectively. These will be modified for use in other databases. A two-pronged approach will be adapted, firstly search strategy will be restricted to systematic reviews to identify which pregnancy complications/ risk factors will have evidence from systematic reviews. Secondly, we will run a search strategy for primary studies if an update of the review is required. Searches will be updated periodically to identify newly published systematic reviews.

Study selection

Once the literature search is completed, reference management software will be used to manage the identified literature (e.g., Endnote, Mendeley or Reference Manager). After removing duplicate studies, two reviewers will independently conduct the title and abstract screening and ineligible studies will be excluded. Full text screening of eligible studies will be conducted by two reviewers independently and a third senior reviewer will be consulted to resolve any discrepancy. The full text will be translated if non-English language studies are identified. For non-English language articles, the authors will be consulted if they have expertise in the language. Followed by contacting university language departments and

For peer review only - http://bmjopen.bmj.com/site/about/guidelines.xhtml

fellow researchers. If no one is identified with expertise in the language, then a professional will be hired for the translation (41).The list of excluded studies will be maintained with the reasons for exclusion documented. The details of the steps involved in study selection will be reported using the PRISMA flowchart.

Exclusion criteria

The following types of publications will not be included: protocols, review articles, conference abstracts, guidelines, consensus, documents or expert position papers, summaries, comments, letters, and brief reports. The reviews that include theoretical studies or text or opinion as their primary source of evidence will not be included (34, 42).

Quality assessment

Two reviewers will perform the quality assessment of the reviews using AMSTAR 2 tool independently. Out of the 16 points of the AMSTAR 2 tool, one point will be awarded for each of the criteria met(43). The reviews will be rated as high, moderate, low or critically low quality. The critical domains will include protocol registration, literature search detailed and including grey literature, risk of bias assessment of included primary studies, meta-analysis conducted appropriately, risk of bias considered in reporting/interpretation of results and reporting of publication bias will be taken into consideration for rating the review. The reviews will not be excluded even if they are rated as being low quality. Reviews that do not mention the quality assessment of primary studies might be excluded but this will be at the reviewer's discretion. To resolve any disagreements, a third reviewer will be consulted.

Update of existing reviews

Up to 50% of systematic reviews are out of date after 5.5 years (44). The recommended methods for updating existing systematic reviews and meta-analyses will be used where a need for update is identified and the update of the review will only be considered by authors based on these guidelines (45-48). In case the reviews need to be updated, only high and moderate quality systematic reviews will be eligible for updates (45).

Overlap

Overlapping reviews refers to a situation where two or more reviews examine the same research question and may include the same primary studies. The degree of overlap will be presented graphically using a citation matrix. A citation matrix is a plot of the included primary studies in the rows and systematic reviews in the columns. Overlap will be quantified by the method of corrected covered area (CCA) (49, 50). Quality rating will be used as the selection criteria where higher-quality reviews will be considered over lower-quality reviews.

Data extraction

A standardised data extraction form will be used. The data extraction form will be piloted prior to use. Data will be extracted from the final list of included studies which will then be checked by the second reviewer. Two reviewers will be involved in data extraction. After the

For peer review only - http://bmjopen.bmj.com/site/about/guidelines.xhtml

first reviewer has completed the data extraction, the second reviewer will check the data extraction sheet and provide any comments. Any differences will be resolved by discussion and a third reviewer will be consulted if necessary.
The data will be extracted using the following template using Microsoft Excel.

1. Study ID
2. Author/s
3. Year of publication
4. Geographical area
5. Aim of the review
6. Database searched
7. Search period
8. Population
9. Heath care setting
10. Exposures
11. Comparator
12. Outcomes
13. Covariates
14. Study design(s)
15. Definition of exposure
16. Definition of outcome
17. Data synthesis method
18. Quality assessment tool
19. Quality of the included primary studies as assessed by review authors
20. Number of studies included in qualitative analysis (narrative synthesis where meta-analysis was not done / possible by the review authors)
21. Number of meta-analyses
22. Number of studies included in each meta-analysis
23. Summary estimates of each meta-analysis and its related 95% confidence intervals
24. Author's conclusion
25. Review limitation
26. Additional comments

Recently published studies will be added into the narration of the reviews where no meta-analysis has been performed.

Data analysis

Study characteristics will be presented in a tabular format. The study will synthesise the data using both narrative and quantitative methods. Where a meta-analysis has been carried out, we will report the summary result. Where the review does not provide a summary result, we will explore the key findings and use these to inform a narrative overview of the key findings. Based on the data extracted from individual studies, systematic review or meta-analysis, data may need to be converted before pooling. For binary data, pooled odds ratios with 95% confidence intervals (CIs) will be presented. For continuous data, we will use the mean difference or standardised mean difference with 95% CIs. While analysing the results reported in the systematic reviews, there may be differences in population characteristics, so it might not be possible to combine all the results from the

For peer review only - http://bmjopen.bmj.com/site/about/guidelines.xhtml

included studies. If we do combine them, we may present effect sizes according to certain population characteristics such as country/region to account for differences in nutritional status etc. We will try to consider all heterogeneity including clinical, methodological and statistical and will ensure this is covered in the discussion section of the manuscripts of the umbrella reviews. The findings will be presented in forest plots to assess the heterogeneity of the study findings. The I^2 statistic will be used to evaluate statistical heterogeneity. All statistical analysis will be conducted using Stata, Microsoft Excel or R package. Age and ethnicity subgroup analysis will be considered where appropriate. Publication bias will be assessed both quantitatively and using a funnel plot where appropriate (51). Grading of recommendations, assessment, development and evaluations(GRADE) tool will be used to estimate the strength of evidence (52).

Patient Public involvement

Patient and public involvement (PPI) representatives (RP and NM) were involved in formulating the research question and study design. They have also played a key role in collaboration with clinicians and researchers to identify and consider the list of pregnancy complications and health outcomes in the study. NM has been part of our regular meetings and also provided input to improve the manuscript. They will also play a key role in disseminating the results once the reviews have been undertaken.

Ethics and dissemination

No ethical approvals required. Findings will be disseminated through publications in peer reviewed journals and conferences.

Conflict of Interest

No conflict of interest reported.

Acknowledgements

Patient representatives and MuM-PreDiCT team

Authors contributions

MS was responsible for drafting the initial manuscript.KN, FC, ST, MB, KAE, HH, JH, SW, ZV, KP, NM, NC, SIL, ST, KO and KA, RR were responsible for revising the manuscript critically for important intellectual content. The authors have approved the final submitted version and are accountable for all aspects of the work.

Funding

This work was funded by the Strategic Priority Fund “Tackling multimorbidity at scale” programme [grant number-MR/W014432/1 delivered by the Medical Research Council and the National Institute for Health and Care Research in partnership with the Economic and Social Research Council and in collaboration with the Engineering and Physical Sciences Research Council. The funders have no role in development of this protocol.

For peer review only - http://bmjopen.bmj.com/site/about/guidelines.xhtml

References

1. Kramer MS. The epidemiology of adverse pregnancy outcomes: an overview. J Nutr. 2003;133(5 Suppl 2):1592s-6s.
2. Zhang F, Dong L, Zhang CP, Li B, Wen J, Gao W, et al. Increasing prevalence of gestational diabetes mellitus in Chinese women from 1999 to 2008. Diabet Med. 2011;28(6):652-7.
3. Lavery JA, Friedman AM, Keyes KM, Wright JD, Ananth CV. Gestational diabetes in the United States: temporal changes in prevalence rates between 1979 and 2010. Bjog. 2017;124(5):804-13.
4. Bornstein E, Eliner Y, Chervenak FA, Grünebaum A. Concerning trends in maternal risk factors in the United States: 1989–2018. EClinicalMedicine. 2020;29:100657.
5. Wang, W., Xie, X., Yuan, T. et al. Epidemiological trends of maternal hypertensive disorders of pregnancy at the global, regional, and national levels: a population-based study. BMC Pregnancy Childbirth 21, 364 (2021). https://doi.org/10.1186/s12884-021-03809-2. 2021.
6. Alder J, Fink N, Bitzer J, Hösli I, Holzgreve W. Depression and anxiety during pregnancy: a risk factor for obstetric, fetal and neonatal outcome? A critical review of the literature. The Journal of Maternal-Fetal & Neonatal Medicine. 2007;20(3):189-209.
7. Ashrafi M, Sheikhan F, Arabipoor A, Hosseini R, Nourbakhsh F, Zolfaghari Z. Gestational diabetes mellitus risk factors in women with polycystic ovary syndrome (PCOS). European Journal of Obstetrics & Gynecology and Reproductive Biology. 2014;181:195-9.
8. Bai W, Li Y, Niu Y, Ding Y, Yu X, Zhu B, et al. Association between ambient air pollution and pregnancy complications: a systematic review and meta-analysis of cohort studies. Environmental research. 2020;185:109471.
9. Kęska K, Szcześniak MW, Adamus A, Czernicka M. Waterlogging-stress-responsive LncRNAs, their regulatory relationships with miRNAs and target genes in cucumber (Cucumis sativus L.). International journal of molecular sciences. 2021;22(15):8197.
10. Langley-Evans SC, Pearce J, Ellis S. Overweight, obesity and excessive weight gain in pregnancy as risk factors for adverse pregnancy outcomes: A narrative review. Journal of Human Nutrition and Dietetics. 2022;35(2):250-64.
11. Patra J, Bakker R, Irving H, Jaddoe VW, Malini S, Rehm J. Dose–response relationship between alcohol consumption before and during pregnancy and the risks of low birthweight, preterm birth and small for gestational age (SGA)—a systematic review and meta-analyses. BJOG: An International Journal of Obstetrics & Gynaecology. 2011;118(12):1411-21.
12. Scannapieco FA, Bush RB, Paju S. Periodontal disease as a risk factor for adverse pregnancy outcomes. A systematic review. Annals of Periodontology. 2003;8(1):70-8.
13. World health organisation,Maternal mortalityhttps://www.who.int/news-room/fact-sheets/detail/maternal-mortality(16/02/2022) ,(assessed Feb,2022).
14. Dr Lale Say M, Doris Chou, MD,Alison Gemmill, MPH,Özge Tunçalp, MD,Ann-Beth Moller, MSc,Jane Daniels, PhD,A Metin Gülmezoglu, MD,Marleen Temmerman, MD,Leontine Alkema, PhD. Global causes of maternal death: a WHO systematic analysis. 2014;VOLUME 2,(ISSUE 6, E323-E333).
15. World health organisation,maternal deaths fact sheet,https://www.who.int/news-room/fact-sheets/detail/maternal-mortality,22nd february,2022.
16. Sattar N, Greer IA. Pregnancy complications and maternal cardiovascular risk: opportunities for intervention and screening? BMJ. 2002 Jul 20;325(7356):157-60. doi: 10.1136/bmj.325.7356.157. PMID: 12130616; PMCID: PMC1123678.,(assessed Feb,2022). 2002.
17. Neiger R. Long-Term Effects of Pregnancy Complications on Maternal Health: A Review. Journal of Clinical Medicine. 2017;6(8):76.
18. Panaitescu AM PM, Ciobanu AM, Gica N, Cimpoca-Raptis BA. . Pregnancy Complications Can Foreshadow Future Disease-Long-Term Outcomes of a Complicated Pregnancy. Medicina (Kaunas). 2021;57(12):1320. Published 2021 Dec 1. doi:10.3390/medicina57121320. 2021.
19. Williams D. Pregnancy: a stress test for life. Curr Opin Obstet Gynecol. 2003 Dec;15(6):465-71. doi: 10.1097/00001703-200312000-00002. PMID: 14624211..,(assessed Feb,2022)

For peer review only - http://bmjopen.bmj.com/site/about/guidelines.xhtml

2003.
20. Bellamy L, Casas JP, Hingorani AD, Williams DJ. Pre-eclampsia and risk of cardiovascular disease and cancer in later life: systematic review and meta-analysis. Bmj. 2007;335(7627):974.
21. Robbins CL, Hutchings Y, Dietz PM, Kuklina EV, Callaghan WM. History of preterm birth and subsequent cardiovascular disease: a systematic review. Am J Obstet Gynecol. 2014;210(4):285-97.
22. Rich-Edwards JW. Reproductive health as a sentinel of chronic disease in women. Womens Health (Lond). 2009;5(2):101-5.
23. Wu P, Kwok CS, Haththotuwa R, Kotronias RA, Babu A, Fryer AA, et al. Pre-eclampsia is associated with a twofold increase in diabetes: a systematic review and meta-analysis. Diabetologia. 2016;59(12):2518-26.
24. Knight M BK, Tuffnell D, Shakespeare J, Kotnis R, Kenyon S, Kurinczuk JJ (Eds.) on behalf of MBRRACE-UK. Saving Lives, Improving Mothers' Care - Lessons learned to inform maternity care from the UK and Ireland Confidential

Enquiries into Maternal Deaths and Morbidity 2015-17. Oxford: National Perinatal Epidemiology Unit, University of

Oxford 2019

2015-17.
25. Brier N. Anxiety after miscarriage: a review of the empirical literature and implications for clinical practice. Birth. 2004;31(2):138-42.
26. Anderson C, Cacola P. Implications of preterm birth for maternal mental health and infant development. MCN: The American Journal of Maternal/Child Nursing. 2017;42(2):108-14.
27. Brind J, Chinchilli VM, Severs WB, Summy-Long J. Induced abortion as an independent risk factor for breast cancer: a comprehensive review and meta-analysis. J Epidemiol Community Health. 1996;50(5):481-96.
28. Wingo PA, Newsome K, Marks JS, Calle EE, Parker SL. The risk of breast cancer following spontaneous or induced abortion. Cancer Causes Control. 1997;8(1):93-108.
29. Burlina S, Dalfrà MG, Chilelli NC, Lapolla A. Gestational Diabetes Mellitus and Future Cardiovascular Risk: An Update. International Journal of Endocrinology. 2016;2016:2070926.
30. Daly B, Toulis KA, Thomas N, Gokhale K, Martin J, Webber J, et al. Increased risk of ischemic heart disease, hypertension, and type 2 diabetes in women with previous gestational diabetes mellitus, a target group in general practice for preventive interventions: a population-based cohort study. PLoS medicine. 2018;15(1):e1002488.
31. Brennan P, Bankhead C, Silman A, Symmons D, editors. Oral contraceptives and rheumatoid arthritis: results from a primary care-based incident case-control study. Seminars in arthritis and rheumatism; 1997: Elsevier.
32. Jørgensen KT, Pedersen BV, Jacobsen S, Biggar RJ, Frisch M. National cohort study of reproductive risk factors for rheumatoid arthritis in Denmark: a role for hyperemesis, gestational hypertension and pre-eclampsia? Annals of the rheumatic diseases. 2010;69(2):358-63.
33. Okoth K, Chandan JS, Marshall T, Thangaratinam S, Thomas GN, Nirantharakumar K, et al. Association between the reproductive health of young women and cardiovascular disease in later life: umbrella review. BMJ. 2020;371.
34. Institute TJB. Joanna Briggs Institute Reviewers' Manual: 2014 edition / Supplement. 2014.
35. PRISMA transparent reporting of systematic reviews and meta-analysis https://www.prisma-statement.org/Extensions/Protocols,assessed Nov,2022.
36. Fusar-Poli P, Radua J. Ten simple rules for conducting umbrella reviews. Evidence Based Mental Health. 2018;21(3):95-100.
37. Cant R, Ryan C, Kelly MA. A nine-step pathway to conduct an umbrella review of literature. Nurse Author & Editor.
38. Page MJ, McKenzie JE, Bossuyt PM, Boutron I, Hoffmann TC, Mulrow CD, et al. The PRISMA 2020 statement: an updated guideline for reporting systematic reviews. Bmj. 2021;372:n71.

For peer review only - http://bmjopen.bmj.com/site/about/guidelines.xhtml

39. Smith V, Devane D, Begley CM, Clarke M. Methodology in conducting a systematic review of systematic reviews of healthcare interventions. BMC medical research methodology. 2011;11(1):1-6.
40. Haddaway NR, Land M, Macura B. “A little learning is a dangerous thing”: A call for better understanding of the term ‘systematic review’. Environment International. 2017;99:356-60.
41. Rockliffe L. Including non-English language articles in systematic reviews: A reflection on processes for identifying low-cost sources of translation support. Research Synthesis Methods. 2022;13(1):2-5.
42. (ed.) GB-Z. Umbrella Reviews: Evidence Synthesis with Overviews of Reviews and Meta-Epidemiologic Studies, chapter 1 page 6.6.
43. Shea BJ, Reeves BC, Wells G, Thuku M, Hamel C, Moran J, et al. AMSTAR 2: a critical appraisal tool for systematic reviews that include randomised or non-randomised studies of healthcare interventions, or both. Bmj. 2017;358:j4008.
44. Bougioukas KI, Liakos A, Tsapas A, Ntzani E, Haidich A-B. Preferred reporting items for overviews of systematic reviews including harms checklist: a pilot tool to be used for balanced reporting of benefits and harms. Journal of clinical epidemiology. 2018;93:9-24.
45. Garner P, Hopewell S, Chandler J, MacLehose H, Akl EA, Beyene J, et al. When and how to update systematic reviews: consensus and checklist. BMJ. 2016;354:i3507.
46. Moher D, Tsertsvadze A, Tricco A, Eccles M, Grimshaw J, Sampson M, et al. When and how to update systematic reviews. Cochrane database of systematic reviews. 2008(1).
47. Moher D, Tsertsvadze A, Tricco AC, Eccles M, Grimshaw J, Sampson M, et al. A systematic review identified few methods and strategies describing when and how to update systematic reviews. Journal of clinical epidemiology. 2007;60(11):1095. e1-. e11.
48. Sampson M, Shojania KG, McGowan J, Daniel R, Rader T, Iansavichene AE, et al. Surveillance search techniques identified the need to update systematic reviews. Journal of clinical epidemiology. 2008;61(8):755-62.
49. Lunny C, Pieper D, Thabet P, Kanji S. Managing overlap of primary study results across systematic reviews: practical considerations for authors of overviews of reviews. BMC Med Res Methodol. 2021;21(1):140.
50. Hennessy EA, Johnson BT. Examining overlap of included studies in meta-reviews: Guidance for using the corrected covered area index. Research synthesis methods. 2020;11(1):134-45.
51. Egger M, Smith GD, Schneider M, Minder C. Bias in meta-analysis detected by a simple, graphical test. Bmj. 1997;315(7109):629-34.
52. Brozek JL, Canelo-Aybar C, Akl EA, Bowen JM, Bucher J, Chiu WA, et al. GRADE Guidelines 30: the GRADE approach to assessing the certainty of modeled evidence—An overview in the context of health decision-making. Journal of clinical epidemiology. 2021;129:138-50.

For peer review only

For peer review only - http://bmjopen.bmj.com/site/about/guidelines.xhtml

The association of pregnancy complications/risk factors with the development of future long-term health conditions in women: overarching protocol for umbrella reviews

Appendix/Supplementary material

- Appendix Table 1: PRISMA-P (Preferred Reporting Items for Systematic review and Meta-Analysis Protocols) 2015 checklist: recommended items to address in a systematic review protocol).
- Appendix Table 2: Definitions of pregnancy complications and risk factors
- Appendix Table 3: Search terms for pregnancy complications/risk factors (exposure)
- Appendix Table 4: Search terms for long-term health conditions (outcomes)
- Appendix Table 5.1 Search strategy- Medline Database-Autoimmune condition and pregnancy complications
- Appendix Table 5.2 search strategy- Medline database- Cancer and pregnancy complications
- Appendix Table 5.3 search strategy- Medline database- Functional conditions and pregnancy complications
- Appendix Table 5.4 search strategy- Medline database- Mental health condition and pregnancy complications
- Appendix Table 5.5 search strategy- Medline database- Metabolic conditions and pregnancy complications

Appendix Table 1: PRISMA-P (Preferred Reporting Items for Systematic review and Meta-Analysis Protocols) 2015 checklist: recommended items to address in a systematic review protocol

PRISMA-P (Preferred Reporting Items for Systematic review and Meta-Analysis Protocols) 2015 checklist: recommended items to address in a systematic review protocol*

Section and topic	Item No	Checklist item
**ADMINISTRATIVE INFORMATION**		
Title:		
Identification	1a	Identify the report as a protocol of a systematic review-**Page 4 lines 143-149**
Update	1b	If the protocol is for an update of a previous systematic review, identify as such-**NA**
Registration	2	If registered, provide the name of the registry (such as PROSPERO) and registration number-**Page 4 line 147**
Authors:		
Contact	3a	Provide name, institutional affiliation, e-mail address of all protocol authors; provide physical mailing address of corresponding author-**Page 1 and 2 lines 4-47**
Contributions	3b	Describe contributions of protocol authors and identify the guarantor of the review-**Page 10 lines 347-352**

For peer review only - http://bmjopen.bmj.com/site/about/guidelines.xhtml

Amendments	4	If the protocol represents an amendment of a previously completed or published protocol, identify as such and list changes; otherwise, state plan for documenting important protocol amendments-**NA**
Support:		
Sources	5a	Indicate sources of financial or other support for the review-**Page 10, lines 354-359**
Sponsor	5b	Provide name for the review funder and/or sponsor- **Page 10, line 354**
Role of sponsor or funder	5c	Describe roles of funder(s), sponsor(s), and/or institution(s), if any, in developing the protocol-**Page 10, lines 359**
**INTRODUCTION**		
Rationale	6	Describe the rationale for the review in the context of what is already known **Page 4 and 5 lines 94-142**
Objectives	7	Provide an explicit statement of the question(s) the review will address with reference to participants, interventions, comparators, and outcomes (PICO)-**Page 4 lines 161-199**
**METHODS**		
Eligibility criteria	8	Specify the study characteristics (such as PICO, study design, setting, time frame) and report characteristics (such as years considered, language, publication status) to be used as criteria for eligibility for the review-**Page 5 lines 168-200**
Information sources	9	Describe all intended information sources (such as electronic databases, contact with study authors, trial registers or other grey literature sources) with planned dates of coverage-**Page 7 lines 201-216**
Search strategy	10	Present draft of search strategy to be used for at least one electronic database, including planned limits, such that it could be repeated **Appendix table 5.1-5.5**
Study records:		
Data management	11a	Describe the mechanism(s) that will be used to manage records and data throughout the review-**Page 7 lines 217-218**
Selection process	11b	State the process that will be used for selecting studies (such as two independent reviewers) through each phase of the review (that is, screening, eligibility and inclusion in meta-analysis)-**Page 7 line 218-226**
Data collection process	11c	Describe planned method of extracting data from reports (such as piloting forms, done independently, in duplicate), any processes for obtaining and confirming data from investigators-**Page 8 and 9 lines 267-304**
Data items	12	List and define all variables for which data will be sought (such as PICO items, funding sources), any pre-planned data assumptions and simplifications-**Appendix table 1**
Outcomes and prioritization	13	List and define all outcomes for which data will be sought, including prioritization of main and additional outcomes, with rationale-**Table 2 main document**
Risk of bias in individual studies	14	Describe anticipated methods for assessing risk of bias of individual studies, including whether this will be done at the outcome or study level, or both; state how this information will be used in data synthesis-**Page 8 lines 238-248**
Data synthesis	15a	Describe criteria under which study data will be quantitatively synthesised-**Page 9 and 10 lines 306-327**
	15b	If data are appropriate for quantitative synthesis, describe planned summary measures, methods of handling data and methods of combining data from studies, including any planned exploration of consistency (such as I^2, Kendall's τ)- **Page 9 and 10 lines 306-327**

For peer review only - http://bmjopen.bmj.com/site/about/guidelines.xhtml

For peer review only

	15c	Describe any proposed additional analyses (such as sensitivity or subgroup analyses, meta-regression)- **Page 10 line 324**
	15d	If quantitative synthesis is not appropriate, describe the type of summary planned- **Page 10 line 308**
Meta-bias(es)	16	Specify any planned assessment of meta-bias(es) (such as publication bias across studies, selective reporting within studies)**-Page 10 line 325**
Confidence in cumulative evidence	17	Describe how the strength of the body of evidence will be assessed (such as GRADE)- **Page 10 line 326**

*** It is strongly recommended that this checklist be read in conjunction with the PRISMA-P Explanation and Elaboration (cite when available) for important clarification on the items. Amendments to a review protocol should be tracked and dated. The copyright for PRISMA-P (including checklist) is held by the PRISMA-P Group and is distributed under a Creative Commons Attribution Licence 4.0.**

From: Shamseer L, Moher D, Clarke M, Ghersi D, Liberati A, Petticrew M, Shekelle P, Stewart L, PRISMA-P Group. Preferred reporting items for systematic review and meta-analysis protocols (PRISMA-P) 2015: elaboration and explanation. BMJ. 2015 Jan 2;349(jan02 1):g7647.

Appendix Table 2: Definitions of pregnancy complications and risk factors

**Pregnancy Specific factors**	**Definitions**
Miscarriage/ Recurrent miscarriage/ Spontaneous pregnancy loss.	Spontaneous loss of a pregnancy before 24 weeks of gestation.<br>Early miscarriage, before 13 weeks of gestation.<br>Late miscarriage, between 13 and 24 weeks of gestation<br>Recurrent miscarriage is loss of three or more consecutive pregnancies before 24 weeks of gestation (36).
Stillbirth	child born after the 24th week of pregnancy who did not show any signs of life after being born(1).
Pre-eclampsia- early and late onset<br>Recurrent pre-eclampsia	New onset of hypertension (over 140 mmHg systolic or over 90 mmHg diastolic) after 20 weeks of pregnancy and the coexistence of 1 or more of the following new-onset conditions:<br>- proteinuria<br>- other maternal organ dysfunction (renal insufficiency, liver involvement, neurological complications, haematological complications)<br>- uteroplacental dysfunction(2).
Eclampsia<br>HELLP	The new onset of seizures or coma in a pregnant woman with pre-eclampsia<br>HELLP (Hemolysis, Elevated Liver enzymes and Low Platelets) syndrome is a life-threatening pregnancy complication usually considered to be a variant of pre-eclampsia(2).
Gestational hypertension	New hypertension presenting after 20 weeks of pregnancy without significant proteinuria(2).
Placenta previa	Placenta previa is low lying placenta after 20 weeks of pregnancy(3).

For peer review only - http://bmjopen.bmj.com/site/about/guidelines.xhtml

Placenta accreta	Placenta accrete is where placenta is stuck to the uterine muscle(3).
Placenta percreta	Placenta percreta is a condition where placenta attaches itself and grows through the uterus and potentially to the nearby organs (such as the bladder)(3).
Placental abruption	Placental abruption is the early separation of a placenta from the lining of the uterus before completion of the second stage of labor. It is one of the causes of bleeding during the second half of pregnancy. Placental abruption is also called abruptio placentae(4, 5).
Hyperemesis gravidarum	Severe, protracted nausea and vomiting associated with weight loss of more than 5% of pre pregnancy weight, dehydration and electrolyte imbalances(6).
Gestational diabetes mellitus	hyperglycaemia in pregnancy that is above diagnostic thresholds for diabetes<br>• a fasting plasma glucose level of 5.6 mmol/litre or above or<br>• a 2-hour plasma glucose level of 7.8 mmol/litre or above(7).
Ectopic pregnancy	If the fertilised egg fails to move from the fallopian tube into the uterus, where the pregnancy grows and develops and the fertilised egg may implant and start to develop outside the uterus(8).
Molar pregnancy/ Choriocarcinoma	Abnormal cells or tumours that start in the womb from cells that would normally develop into the placenta are called Gestational trophoblastic disease (GTD).<br>Gestational trophoblastic disease (GTD) forms a group of disorders spanning the conditions of complete and partial molar pregnancies through to the malignant conditions of invasive mole, choriocarcinoma(9, 10).
Multiple pregnancy/Twin-pregnancies/ Multiple gestation	Term used when one is expecting two or more babies at the same time (twins, triplets or more)(11).
Obstetric / postpartum haemorrhage (PPH)	Heavy bleeding after birth postpartum haemorrhage (PPH) can be primary or secondary. Primary PPH -blood loss 500 ml (a pint) or more of blood within the first 24 hours after the birth of the baby. Secondary PPH occurs when there is abnormal or heavy vaginal bleeding between 24 hours and 12 weeks after the birth(12).
Pre-term birth/ Recurrent pre-term birth	Babies born alive before 37 weeks of pregnancy are completed. extremely preterm (less than 28 weeks),very preterm (28 to 32 weeks),moderate to late preterm (32 to 37 weeks)(13).

For peer review only - http://bmjopen.bmj.com/site/about/guidelines.xhtml

Mode of birth: Instrumental	Birth of a singleton/multiple baby/babies between 37+0 and 42+6 weeks of gestation, who give birth vaginally with the assistance of instruments(ventouse and forceps births)(14).
Mode of birth Caesarean	Birth of a singleton/ multiple baby/babies baby between 37+0 and 42+6 weeks of gestation, by caesarean section(elective or emergency)(14).
Low birth weight	Weight at birth of < 2500 grams (5.5 pounds)(15).
Small for gestational age	Small for gestational age Term babies with a birth weight below the 10th centile, and below the 2nd centile are provided for reference(14).
Intra-uterine growth retardation Intra uterine growth restriction	Growth restriction implies a pathological restriction of the genetic growth potential. As a result, growth restricted fetuses may manifest evidence of fetal compromise (abnormal Doppler studies, reduced liquor volume).<br>Small for gestational age Term babies with a birth weight below the 10th centile, and below the 2nd centile are provided for reference(14).
Fetal growth restriction	fetal growth restriction is defined as birth weight <3rd centile. We used severe small for gestational age at birth as a proxy for fetal growth restriction(16).
Post-partum depression	Depression like symptoms including low mood etc that starts within one or two months or several months of giving birth. Symptoms might last at least two weeks depending on the severity(17).
Puerperal psychosis	Puerperal psychosis is a severe mental illness that starts suddenly in the days, or weeks, after having a baby. Symptoms vary and can include high mood (mania), depression, confusion, hallucinations and delusions(18).
Perineal trauma-3rd and 4th degree tears	Severe perineal tears extending into the anal sphincter, anal mucosa are known as “third degree” and “fourth degree” tears also known as obstetric anal sphincter injury – OASI)(14, 19).
Obstetric cholestasis	Disorder that affects your liver during pregnancy causing build-up of bile acids in the body. The main symptom is itching of the skin and no rash. The symptoms get better when your baby has been born(20).
Pelvic girdle pain (PGP)	Pelvic girdle pain or symphysis pubis dysfunction (SPD).refers to pain in the front and/or the back of your pelvis .It also affect other areas such as the hips or thighs and the sacroiliac joints at the back and/or the symphysis pubis joint at the front(21).

Appendix Table 3. Search terms for pregnancy complications / risk factors (exposure)

For peer review only - http://bmjopen.bmj.com/site/about/guidelines.xhtml

Pregnancy complications/risk factors	pregnancy complication*.mp. or exp Pregnancy Complications/
Miscarriage/ Recurrent miscarriage/ Spontaneous pregnancy loss	exp Abortion, Spontaneous/ or ((recurrent adj3 miscarr$) or miscarr$ or early pregnancy loss$).mp.
Stillbirth	(stillbirth or still birth).mp. or exp Stillbirth/ or exp Fetal Death/ or (f?etal death$ or f?etal demise$).mp.
Hypertensive disorders of pregnancy /Gestational hypertension	Hypertension, Pregnancy-Induced/ or (gestational hypertension or (pregnancy adj3 hypertensi$)).mp.
Pre-eclampsia- early or late onset	(preeclampsia or pre-eclampsia).mp. or exp Pre-Eclampsia/
Eclampsia	exp Eclampsia/ or (eclampsia or tox?emia).mp.
HELLP (Homolysis, Elevated Liver enzymes and Low Platelets) syndrome	HELLP.mp. or exp HELLP Syndrome/
Placenta previa	placenta accreta.mp. or exp Placenta Accreta/ or placenta percreta.mp. or placenta increta.mp. or morbidly adherent placenta.mp. or abnormally invasive placenta.mp.
Placental abruption	Placenta$ abruption.mp. or exp Abruptio Placentae/
Placenta accrete and placenta percreta	placenta pr?eia.mp. or exp placenta previa/ or exp low lying placenta/
Hyperemesis gravidarum	Hyperemesis Gravidarum.mp. or Hyperemesis Gravidarum/ or morning sickness.mp. or exp Morning Sickness/
Gestational diabetes mellitus	(((pregnan$ or gestation$ or prenatal$ or antenatal$ or pre-natal$ or ante-natal$ or maternal$) adj2 diabet$) or gestational diabetes).mp. or exp Diabetes, Gestational/ or GDM.mp.
Ectopic pregnancy	ectopic pregnancy.mp. or exp Pregnancy, Ectopic/ or ((tub$$ adj3 pregnanc$) or (cornual adj3 pregnanc$) or (heterotopic adj3 pregnanc$) or (abdomin$ adj3 pregnanc$) or (extrauterine adj3 pregnanc$) or (interstitial adj3 pregnanc$) or (cervi$ adj3 pregnanc$) or (ovar$ adj3 pregnanc$) or (cesarean scar adj3 pregnanc$)).mp.
Molar pregnancy/ Choriocarcinoma	exp Gestational Trophoblastic Disease/ or gestational trophoblastic.mp. or exp Hydatidiform Mole/ or ((hydatid? adj2 mole?) or (molar adj2 pregnanc?)).mp.

For peer review only

For peer review only - http://bmjopen.bmj.com/site/about/guidelines.xhtml

	exp Choriocarcinoma/ or choriocarcinoma.mp.
Multiple pregnancy/Twin-pregnancies/ Multiple gestation	exp Pregnancy, Multiple/ or ((pregnanc* or gestation*) adj (twin* or triplet* or quadruplet* or quintuplet* or multiple or multi?f?et*)).mp. or (Monochorionic or dichorionic).mp.
Obstetric haemorrhage (postpartum)	exp Postpartum Hemorrhage/ or (postpartum hemorrhage or post partum hemorrhage or postpartum haemorrhage or post partum haemorrhage).ti,ab. or obstetric haemorrhage.mp.
Pre-term birth/ Recurrent pre-term birth	obstetric labor, premature.mp. or exp Obstetric Labor, Premature/ or (premature labor or premature labor or preterm labor or preterm labor or preterm birth).mp.
Mode of delivery: Caesarean or Instrumental	exp Cesarean Section, Repeat/ or cesarean.mp. or exp Cesarean Section/ or (caesarean or cesarean or caesarian or cesarian or cesarien or caesarien or c-section or c section).mp.<br><br>exp Extraction, Obstetrical/ or exp Obstetrical Forceps/ or ((operative or instrumental or assisted or forcep* or ventouse* or vacuum*) adj1 (deliver* or birth*)).mp.
Low birth weight	low birth weight.mp. or exp Infant, Low Birth Weight/ or (low birth weight* adj4 very low birth weight*).mp.
Small for gestational age	exp Infant, Small for Gestational Age/ or small for gestational age.mp. or (small adj3 gestational age).mp.
Intra-uterine growth retardation / intra-uterine growth restriction<br><br>Fetal growth retardation	(intra?uterine growth adj2 (restriction* or retardation)).mp. or iugr.ti,ab<br>fetal growth retardation.mp. or exp Fetal Growth Retardation/ or (fetal growth adj2 (restriction? or retardation)).mp.
postpartum depression	postpartum depression.mp. or exp Depression, Postpartum/
Puerperal psychosis	((postpartum* or post partum* or post-partum* or postnatal* or post natal* or post-natal* or perinatal* or peri natal* or peri-natal* or puerp*) and (depress* or

For peer review only - http://bmjopen.bmj.com/site/about/guidelines.xhtml

	dysthymi* or adjustment disorder* or mood disorder* or affective disorder*)).mp.<br><br>(((postpartum* or post partum* or post-partum* or postnatal* or post natal* or post-natal or perinatal* or peri natal* or peri-natal* or puerp*) and (psychos#s or psychotic)) or psychosis after childbirth).mp.
Perineal trauma(3rd and 4th degree)	(((third or fourth or 3rd or 4th) adj degree) and tear*).mp.<br><br>(((anal near adj2 sphincter) or (rectal adj mucosa) or rectum or (anal adj epithelium) or anus or (recto?vaginal adj2 fistulae) or (anorectal adj mucosa) or (anal adj skin)) and (tear* or injur* or damage* or lacerat* or rupture* or trauma)).mp.<br><br>((obstetric* and anal and sphincter and injur*) or (anal and sphincter and injur*)).mp.
Obstetric cholestasis	(exp Pregnancy/ or exp Obstetrics/ or (pregnan* or obstetric*).mp.) and (exp Cholestasis/ or exp Cholestasis, Intrahepatic/)
Pelvic girdle pain(PGP)	pelvic girdle pain.mp. or exp Pelvic Girdle Pain/ or(symphysis pubis adj3 (pain$ or dysfunction$)).mp. or Pubic Symphysis Diastasis.mp. or exp Pubic Symphysis Diastasis/ or Sacroiliac joint dysfunction.mp. or PGP.mp.

Appendix Table 4: Search terms for long-term health conditions (outcomes)

**1-Autoimmune health conditions**	
**Health conditions**	**Search terms**
Autoimmune conditions	exp Autoimmune Diseases/ or (auto?immune adj2 (disease* or condition*)).mp.
Psoriasis	exp Psoriasis/ or psoria$.mp.
Vitiligo	vitiligo.mp. or exp Vitiligo/ or (leucoderma or leukoderma).mp.
Alopecia areata	alopecia areata.mp. or exp Alopecia Areata/ or (alopecia adj totalis).mp. or (alopecia adj universalis).mp.

For peer review only - http://bmjopen.bmj.com/site/about/guidelines.xhtml

Systemic lupus erythematosus	exp Lupus Erythematosus, Systemic/ or systemic lupus.mp. or sle.mp.
Psoriatic arthritis	exp Arthritis, Psoriatic/ or psoriatic.mp. or (psoria? adj (arthriti? or arthropath?)).mp. or ((arthriti? or arthropath?) adj psoria?).mp.
Ankylosing spondylitis	Ankylosing spondylitis.mp. or exp Spondylitis, Ankylosing/
Rheumatoid arthritis	exp Arthritis, Rheumatoid/ or rheumatoid artheritis.mp. or ((rheumatoid or reumatoid or revmatoid or rheumatic or reumatic or revmatic or rheumat$ or reumat$ or revmarthrit$) adj3 (arthrit$ or artrit$ or diseas$ or condition$ or nodule$)).mp.
Sjögren's's syndrome	exp Sjogren's Syndrome/ or sjogren.mp.
Systemic sclerosis	Systemic Sclerosis.mp. or exp Scleroderma, Systemic/ or ((systemic adj scler?) or sclero?).mp. or crest syndrome.mp. or exp CREST Syndrome/
Coeliac disease	celiac disease.mp. or exp Celiac Disease/ or (c?eliac adj disease*).mp.
Ulcerative colitis	ulcerative colitis.mp. or exp Colitis, Ulcerative/
Crohn's disease	Crohn's disease.mp. or exp Crohn Disease/
Inflammatory bowel disease	exp Inflammatory Bowel Diseases/ or inflammatory bowel.mp.
Multiple sclerosis	multiple sclerosis.mp. or exp Multiple Sclerosis/
Graves' disease	graves disease.mp. or exp Graves Disease/ or (grave? adj6 (diseas? or thyrotoxicos? or hyperthyr?)).mp.
Hashimoto's disease	exp Thyroiditis, Autoimmune/ or or autoimmune thyroid.mp. or Hashimoto Disease/
Type 1 diabetes	type 1 diabetes.mp. or exp Diabetes Mellitus, Type 1/ or ("typ? 1 diabet*" or "typ? I diabet*" or "typ?1 diabet*" or "typ?I diabet*").mp. or (("insulin* depend*" or "insulin?depend*") not ("non-insulin* depend*" or "non insulindepend*")).mp. or (IDDM or T1DM or T1D).mp. or autoimmune diabetes.mp.
Myasthenia gravis	exp Myasthenia Gravis/ or mysathenia.mp.
Addison's disease	exp Addison Disease/ or addison.mp.
**2-Cancers**	
Lung (C33-34)	exp Lung Neoplasms/ or lung cancer c33 c34.mp.
Breast (C50)	exp Breast Neoplasms/ or breast cancer.mp. or breast cancer c50.mp.
Colorectal	(colorectal or colon$ or rectal or rectum or sigmoid).ti,ab,sh.) And (cancer$ or neoplasm$

For peer review only - http://bmjopen.bmj.com/site/about/guidelines.xhtml

	or or tumor or carcinoma or adenoma or adenocarcinoma).ti,ab,sh.)
Cervix	Uterine Cervical Neoplasms/ or cancer cervix.mp. or cervical cancer.mp. or cervical neoplasms.mps or cer$ cancer.mp.
Cancer of unknown primary (c77-c80)	Neoplasms, Unknown Primary/ or cancer of unknown primary.mp.
Pancreas (c25)	exp Pancreatic Neoplasms/ or cancer pancrea$.mp. or Carcinoma, Pancreatic Ductal or pancreas$ carcinoma.mp.
Ovary (c56- c57.4)	Exp Ovarian Neoplasms/ or ((ovarian or ovary or ovar$) adj2 carcinoma).tw. or ovary c56.mp.
Uterus (c54-c55)	Exp Uterine Neoplasms/ or Endometrial neoplasms/ or cancer, uter$.mp. or ((uterine or uterus or uter$) adj2 carcinoma).tw. or uterine c54.mp.
Oesophagus (c15)	exp Esophageal Neoplasms/ or ((esoph$ or oesoph$) adj3 (carcinoma or cancer)).tw.
Brain, Other CNS & Intracranial Tumours (C70-C72, C75.1-C75.3, D32-D33, D35.2-D35.4, D42-D43, D44.3-D44.5)	exp Central Nervous System Neoplasms/ or brain c70.mp. or brain d33.mp.
Liver (c22)	Exp Liver Neoplasms/ or liver cancer c22.mp.
Melanoma Skin Cancer (c43)	exp Skin neoplasms/ or exp Carcinoma, Basal cell/ or melanoma skin cancer.mp. or melanoma c43.mp. or BCC.mp.
Non-Hodgkin Lymphoma (c82-c86)	Exp Lymphoma, Non-Hodgkin/ or non-hodgkin lymphoma.mp.
Kidney (c64-66, c68)	Exp Kidney Neoplasms/ or kidney cancer.mp. or kidney c64.mp. or Carcinoma, Renal Cell/ or renal cancer.mp. or ((kidney or rena? or neph$) adj3 (carcinoma or cancer or neoplas?)).tw.
Thyroid	Exp Thyroid Neoplasms/ or Thyroid cancer.mp. or thyroid carcinoma.mp.
Leukemias	Leukemia/ or leukemia.mp. or leukaemia.mp.
**3-Functional health conditions**	
Chronic pain	exp Chronic Pain/
Fibromyalgia	fibromyalgia.mp. or exp FIBROMYALGIA/
Low back pain	exp Low Back Pain/ or exp Back Pain/ or chronic back pain.mp.
Interstitial cystitis	interstitial cystitis.mp. or exp Cystitis, Interstitial/
Irritable bowel syndrome	irritable bowel syndrome.mp. or exp Irritable Bowel Syndrome/

For peer review only

For peer review only - http://bmjopen.bmj.com/site/about/guidelines.xhtml

Vulvodynia	vulvodynia.mp. or exp VULVODYNIA/
Tension headache	tension headache.mp. or exp Tension -Type Headache/
**4-Mental health condition**	
Serious mental illness	((affective and non-affective) or (psychos*s or psychotic) or (bipolar* or bi-polar* or mania* or manic-depress**) or (schizophrenia* or schizo-affective or schizophrenia*) or (delusion* or paranoi*)).mp.
Common mental illness	(depress* or dysthymi* or mood disorder* or affective disorder* or distress* or neuros*).mp. or (anxiet* or obsessive compulsive disorder* or OCD* PTSD or post-traumatic stress disorder or panic* or neurosis* or neurotic* or adjustment disorder*).mp.
**5-Metabolic health conditions**	
Types 2 diabetes	exp Diabetes Mellitus, Type 2/ or diabetes type 2.mp. or Type 2/ or (MODY or NIDDM or T2DM or T2D).mp. or (non insulin* depend* or noninsulin* depend* or noninsulin?depend* or non insulin?depend*).mp. or ((typ? 2 or typ? II or typ?2 or typ?II) adj3 diabet*).mp. or (((late or adult* or matur* or slow or stabl*) adj3 onset) and diabet*).mp.
Hypertension	exp hypertension/ or hypertens$.mp. or exp blood pressure/ or (blood pressure or bloodpressure).mp.

For peer review only

Appendix Table 5.1 Search strategy- Medline database-Autoimmune condition and pregnancy complications

1	exp Autoimmune Diseases/
2	(auto?immune adj2 (disease* or condition*)).mp.
3	exp Psoriasis/
4	psoria$.mp.
5	exp Vitiligo/
6	vitiligo.mp.
7	(leucoderma or leukoderma).mp.
8	exp Alopecia Areata/
9	alopecia areata.mp.
10	((alopecia adj totalis) or (alopecia adj universalis)).mp.
11	exp Lupus Erythematosus, Systemic/
12	(systemic lupus or sle).mp.
13	arthritis psoriatic.mp. or exp Arthritis, Psoriatic/

For peer review only - http://bmjopen.bmj.com/site/about/guidelines.xhtml

14	Ankylosing spondylitis.mp. or exp Spondylitis, Ankylosing/
15	Spondylarthritis/
16	(axial adj2 spondylarthritis).tw.
17	(axial adj2 spa).tw.
18	(ankylos$ or spondyl$).tw.
19	(bekhterev$ or bechterew$).tw.
20	(Marie adj struempell$).tw.
21	(AS or axSPA).tw.
22	Sacroiliitis/
23	((axial or spin$ or peripheral or vertebral or enthesitis) adj3 (joint$ or spondyloarthritis or arthritis or ankylosing)).tw.
24	exp Arthritis, Rheumatoid/
25	((rheumatoid or reumatoid or revmatoid or rheumatic or reumatic or revmatic or rheumat$ or reumat$ or revmarthrit$) adj3 (arthrit$ or artrit$ or diseas$ or condition$ or nodule$)).mp.
26	sjogren syndrome.mp. or exp Sjogren's Syndrome/
27	exp Celiac Disease/
28	(c?eliac adj disease*).mp.
29	inflammatory bowel disease.mp. or exp Inflammatory Bowel Diseases/
30	ulcerative colitis.mp. or exp Colitis, Ulcerative/
31	Crohn's disease.mp. or exp Crohn Disease/
32	multiple sclerosis.mp. or exp Multiple Sclerosis/
33	Systemic Sclerosis.mp.
34	exp Scleroderma, Systemic/
35	((systemic adj scler?) or sclero?).mp.
36	crest syndrome.mp. or exp CREST Syndrome/
37	graves disease.mp. or exp Graves Disease/
38	(grave? adj3 (diseas? or thyrotoxicos? or hyperthyr?)).mp.
39	exp Thyroiditis, Autoimmune/
40	autoimmune thyroid.mp.
41	Hashimoto Disease.mp.
42	exp Hashimoto Disease/
43	(thyroid adj autoantibodies).mp. [mp=title, book title, abstract, original title, name of substance word, subject heading word, floating sub-heading word, keyword heading word, organism supplementary concept word, protocol supplementary concept word, rare disease supplementary concept word, unique identifier, synonyms]
44	thyroid.mp. and exp autoantibodies/
45	myasthenia gravis.mp. or exp Myasthenia Gravis/
46	addison disease.mp. or exp Addison Disease/
47	type 1 diabetes.mp. or exp Diabetes Mellitus, Type 1/ or ("typ? 1 diabet*" or "typ? I diabet*" or "typ?1 diabet*" or "typ?I diabet*").mp. or (("insulin* depend*" or "insulin?depend*") not ("non-insulin* depend*" or "non insulindepend*")).mp. or (IDDM or T1DM or T1D).mp. or autoimmune diabetes.mp.
48	exp Pregnancy Complications/
49	(pregnancy adj complication?).mp.
50	exp Abortion, Spontaneous/
51	((recurrent adj2 miscarr?) or miscarr?).mp.
52	(early adj3 pregnancy loss?).mp.
53	miscarriage.mp.
54	exp Stillbirth/

For peer review only - http://bmjopen.bmj.com/site/about/guidelines.xhtml

55	(stillbirth or (still adj birth)).mp.
56	exp Fetal Death/
57	((f?etal adj death$) or (f?etal adj demise$)).mp.
58	exp Hypertension, Pregnancy-Induced/
59	(gestational hypertension or (pregnancy adj3 hypertensi$)).mp.
60	(preeclampsia or pre-eclampsia).mp.
61	exp Pre-Eclampsia/
62	(eclampsia or tox?emia).mp.
63	exp Eclampsia/
64	hellp.mp. or exp HELLP Syndrome/
65	exp Placenta Accreta/
66	(placenta adj accreta).mp.
67	placenta percreta.mp.
68	placenta increta.mp.
69	(morbidly adj3 adherent placenta).mp.
70	abnormally invasive placenta.mp.
71	exp Abruptio Placentae/
72	Placenta? abruption.mp.
73	exp Placenta Previa/
74	Placenta pr?evia.mp.
75	low lying placenta.mp.
76	exp Hyperemesis Gravidarum/
77	Hyperemesis Gravidarum.mp.
78	exp Morning Sickness/
79	morning sickness.mp.
80	exp Diabetes, Gestational/
81	GDM.mp.
82	(((pregnan$ or gestation$ or prenatal$ or antenatal$ or pre-natal$ or ante-natal$ or maternal$) adj2 diabet$) or gestational diabetes).mp.
83	((tub$$ adj3 pregnanc$) or (cornual adj3 pregnanc$) or (heterotopic adj3 pregnanc$) or (abdomin$ adj3 pregnanc$) or (extrauterine adj3 pregnanc$) or (interstitial adj3 pregnanc$) or (cervi$ adj3 pregnanc$) or (ovar$ adj3 pregnanc$) or (cesarean scar adj3 pregnanc$)).mp.
84	exp Pregnancy, Ectopic/
85	ectopic pregnancy.mp.
86	exp Gestational Trophoblastic Disease/
87	gestational trophoblastic.mp.
88	exp Hydatidiform Mole/
89	((hydatid? adj2 mole?) or (molar adj2 pregnanc?)).mp.
90	exp Choriocarcinoma/
91	choriocarcinoma.mp.
92	exp Pregnancy, Multiple/
93	((pregnanc* or gestation*) adj (twin* or triplet* or quadruplet* or quintuplet* or multiple or multi?f?et*)).mp.
94	(Monochorionic or dichorionic).mp.
95	exp Postpartum Hemorrhage/
96	(postpartum hemorrhage or post partum hemorrhage or postpartum haemorrhage or post partum haemorrhage).mp.
97	obstetric haemorrhage.mp.
98	obstetric labor, premature.mp.

For peer review only - http://bmjopen.bmj.com/site/about/guidelines.xhtml

99	exp Obstetric Labor, Premature/
100	(premature labor or premature labour or preterm labor or preterm labour or preterm birth).mp.
101	exp Cesarean Section/
102	exp Cesarean Section, Repeat/
103	(caesarean or cesarean or caesarian or cesarian or cesarien or caesarien or c-section or c section).mp.
104	cesarean.mp.
105	exp Extraction, Obstetrical/
106	exp Obstetrical Forceps/
107	((operative or instrumental or assisted or forcep* or ventouse* or vacuum*) adj1 (deliver* or birth*)).mp.
108	exp Infant, Low Birth Weight/
109	low birth weight.mp.
110	(low birth weight* adj4 very low birth weight*).mp.
111	exp Infant, Small for Gestational Age/
112	(small adj3 gestational age).mp.
113	(intra?uterine growth adj2 (restriction* or retardation)).mp.
114	iugr.ti,ab.
115	exp Fetal Growth Retardation/
116	(fetal growth adj2 (restriction? or retardation)).mp.
117	exp Depression, Postpartum/
118	postpartum depression.mp.
119	((postpartum* or post partum* or post-partum* or postnatal* or post natal* or post-natal* or perinatal* or peri natal* or peri-natal* or puerp*) and (depress* or dysthymi* or adjustment disorder* or mood disorder* or affective disorder*)).mp.
120	((postpartum* or post partum* or post-partum* or postnatal* or post natal* or post-natal or perinatal* or peri natal* or peri-natal* or puerp*) and (psychos#s or psychotic)).mp.
121	(psychosis adj3 after childbirth).mp.
122	(((third or fourth or 3rd or 4th) adj degree) and tear*).mp.
123	(((anal near adj2 sphincter) or (rectal adj mucosa) or rectum or (anal adj epithelium) or anus or (recto?vaginal adj2 fistulae) or (anorectal adj mucosa) or (anal adj skin)) and (tear* or injur* or damage* or lacerat* or rupture* or trauma)).mp.
124	((obstetric* and anal and sphincter and injur*) or (anal and sphincter and injur*)).mp.
125	(exp Pregnancy/ or exp Obstetrics/ or (pregnan* or obstetric*).mp.) and (exp Cholestasis/ or exp Cholestasis, Intrahepatic/)
126	pelvic girdle pain.mp. or exp Pelvic Girdle Pain/
127	(symphysis pubis adj3 (pain$ or dysfunction$)).mp.
128	Pubic Symphysis Diastasis.mp. or exp Pubic Symphysis Diastasis/
129	Sacroiliac joint dysfunction.mp.
130	PGP.mp.
131	1 or 2 or 3 or 4 or 5 or 6 or 7 or 8 or 9 or 10 or 11 or 12 or 13 or 14 or 15 or 16 or 17 or 18 or 19 or 20 or 21 or 22 or 23 or 24 or 25 or 26 or 27 or 28 or 29 or 30 or 31 or 32 or 33 or 34 or 35 or 36 or 37 or 39 or 40 or 41 or 42 or 43 or 44 or 45 or 46 or 47
132	48 or 49 or 50 or 51 or 52 or 53 or 54 or 55 or 56 or 57 or 58 or 59 or 60 or 61 or 62 or 63 or 64 or 65 or 66 or 67 or 68 or 69 or 70 or 71 or 72 or 73 or 74 or 75 or 76 or 77 or 78 or 79 or 80 or 81 or 82 or 83 or 84 or 85 or 86 or 87 or 88 or 89 or 90 or 91 or 92 or 93 or 94 or 95 or 96 or 97 or 98 or 99 or 100 or 101 or 102 or 103 or 104 or 105 or 106 or 107 or 108 or 109 or 110 or 111 or 112 or 113 or 114 or 115 or 116 or 117 or 118 or 119 or 120 or 121 or 122 or 123 or 124 or 125 or 126 or 127 or 128 or 129 or 130

For peer review only - http://bmjopen.bmj.com/site/about/guidelines.xhtml

133	131 and 132
134	(systematic$ adj2 (review$ or overview)).ti,ab.
135	(systematic$ adj5 review$).tw,sh.
136	meta-analysis.mp. or exp meta-analysis/
137	134 or 135 or 136
138	133 and 137

Appendix Table 5.2 search strategy- Medline database- Cancer and pregnancy complications

1.	exp Abortion, Spontaneous/
2.	(stillbirth or still birth).mp.
3.	(recurrent adj3 miscarr$).mp. [mp=title, abstract, original title, name of substance word, subject heading word, floating sub-heading word, keyword heading word, organism supplementary concept word, protocol supplementary concept word, rare disease supplementary concept word, unique identifier, synonyms]
4.	(miscarr$ or early pregnancy loss$).mp. [mp=title, abstract, original title, name of substance word, subject heading word, floating sub-heading word, keyword heading word, organism supplementary concept word, protocol supplementary concept word, rare disease supplementary concept word, unique identifier, synonyms]
5.	exp Stillbirth/
6.	exp Fetal Death/
7.	(f?etal death$ or f?etal demise$).mp.
8.	Hypertension, Pregnancy-Induced/
9.	(gestational hypertension or (pregnancy adj3 hypertensi$)).mp.
10.	(preeclampsia or pre-eclampsia).mp.
11.	exp Pre-Eclampsia/
12.	exp Eclampsia/
13.	(eclampsia or tox?emia).mp.
14.	HELLP.mp.
15.	exp HELLP Syndrome/
16.	placenta accreta.mp.
17.	exp Placenta Accreta/
18.	placenta percreta.mp.
19.	placenta increta.mp.
20.	morbidly adherent placenta.mp.
21.	abnormally invasive placenta.mp.
22.	Placenta$ abruption.mp.
23.	exp Abruptio Placentae/
24.	placenta pr?eia.mp.
25.	exp placenta previa/
26.	low lying placenta.mp.
27.	Hyperemesis Gravidarum.mp.
28.	Hyperemesis Gravidarum/

For peer review only

For peer review only - http://bmjopen.bmj.com/site/about/guidelines.xhtml

29.	morning sickness.mp.
30.	exp Morning Sickness/
31.	(((pregnan$ or gestation$ or prenatal$ or antenatal$ or pre-natal$ or ante-natal$ or maternal$) adj2 diabet$) or gestational diabetes).mp.
32.	exp Diabetes, Gestational/
33.	GDM.mp.
34.	ectopic pregnancy.mp.
35.	exp Pregnancy, Ectopic/
36.	((tub$$ adj3 pregnanc$) or (cornual adj3 pregnanc$) or (heterotopic adj3 pregnanc$) or (abdomin$ adj3 pregnanc$) or (extrauterine adj3 pregnanc$) or (interstitial adj3 pregnanc$) or (cervi$ adj3 pregnanc$) or (ovar$ adj3 pregnanc$) or (cesarean scar adj3 pregnanc$)).mp.
37.	exp Gestational Trophoblastic Disease/
38.	gestational trophoblastic.mp.
39.	exp Hydatidiform Mole/
40.	((hydatid? adj2 mole?) or (molar adj2 pregnanc?)).mp.
41.	exp Choriocarcinoma/ or choriocarcinoma.mp.
42.	exp Pregnancy, Multiple/
43.	exp Pregnancy, Multiple/ or ((pregnanc* or gestation*) adj (twin* or triplet* or quadruplet* or quintuplet* or multiple or multi?f?et*)).mp.
44.	(Monochorionic or dichorionic).mp.
45.	exp Postpartum Hemorrhage/
46.	(postpartum hemorrhage or post partum hemorrhage or postpartum haemorrhage or post partum haemorrhage).ti,ab.
47.	obstetric haemorrhage.mp.
48.	obstetric labor, premature.mp.
49.	exp Obstetric Labor, Premature/
50.	(premature labor or premature labour or preterm labor or preterm labour or preterm birth).mp.
51.	exp Cesarean Section, Repeat/ or cesarean.mp.
52.	exp Cesarean Section/
53.	(caesarean or cesarean or caesarian or cesarian or cesarien or caesarien or c-section or c section).mp.
54.	exp Extraction, Obstetrical/
55.	exp Obstetrical Forceps/
56.	((operative or instrumental or assisted or forcep* or ventouse* or vacuum*) adj1 (deliver* or birth*)).mp.
57.	low birth weight.mp.
58.	exp Infant, Low Birth Weight/
59.	(low birth weight* adj4 very low birth weight*).mp.
60.	exp Infant, Small for Gestational Age/
61.	small for gestational age.mp.
62.	(small adj3 gestational age).mp.
63.	(intra?uterine growth adj2 (restriction* or retardation)).mp.
64.	iugr.ti,ab.
65.	fetal growth retardation.mp.

For peer review only - http://bmjopen.bmj.com/site/about/guidelines.xhtml

66.	exp Fetal Growth Retardation/
67.	foetal growth retardation.mp.
68.	(fetal growth adj2 (restriction? or retardation)).mp.
69.	(foetal growth adj2 (restriction? or retardation)).mp.
70.	((postpartum* or post partum* or post-partum* or postnatal* or post natal* or post-natal* or perinatal* or peri natal* or peri-natal* or puerp*) and (depress* or dysthymi* or adjustment disorder* or mood disorder* or affective disorder*)).mp.
71.	(((third or fourth or 3rd or 4th) adj degree) and tear*).mp.
72.	(((anal near adj2 sphincter) or (rectal adj mucosa) or rectum or (anal adj epithelium) or anus or (recto?vaginal adj2 fistulae) or (anorectal adj mucosa) or (anal adj skin)) and (tear* or injur* or damage* or lacerat* or rupture* or trauma)).mp.
73.	((obstetric* and anal and sphincter and injur*) or (anal and sphincter and injur*)).mp.
74.	(exp Pregnancy/ or exp Obstetrics/ or (pregnan* or obstetric*).mp.) and (exp Cholestasis/ or exp Cholestasis, Intrahepatic/)
75	pregnancy complication*.mp.
76	exp Pregnancy Complications/
77	exp Lung Neoplasms/
78.	lung cancer c33 c34.mp.
79	exp Breast Neoplasms/
80	breast cancer.mp.
81	breast cancer c50.mp.
82	Uterine Cervical Neoplasms/
83	cancer cervix.mp.
84	cervical cancer.mp.
85	cervical neoplasms.mp.
86	cer$ cancer.mp.
87	Neoplasms, Unknown Primary/
88.	cancer of unknown primary.mp.
89	exp Pancreatic Neoplasms/
90	cancer pancrea$.mp.
91	Carcinoma, Pancreatic Ductal/
92	pancreas$ carcinoma.mp.
93	exp Ovarian Neoplasms/
94	((ovarian or ovary or ovar$) adj2 carcinoma).tw.
95	ovary c56.mp.
96	exp Uterine Neoplasms/
97	Endometrial neoplasms/
98	cancer, uter$.mp.
99	((uterine or uterus or uter$) adj2 carcinoma).tw.
100	uterine c54.mp.
101	exp Esophageal Neoplasms/
102	((esoph$ or oesoph$) adj3 (carcinoma or cancer)).tw.
103	exp Central Nervous System Neoplasms/
104	brain c70.mp.

For peer review only

For peer review only - http://bmjopen.bmj.com/site/about/guidelines.xhtml

105	brain d33.mp.
106	exp Liver Neoplasms/
107	liver cancer c22.mp.
108	exp Skin neoplasms/
109	melanoma skin cancer.mp.
110	melanoma c43.mp.
111	exp Kidney Neoplasms/
112	kidney cancer.mp.
113	kidney c64.mp.
114	Carcinoma, Renal Cell/
115	renal cancer.mp.
116	((kidney or rena? or neph$) adj3 (carcinoma or cancer or neoplas?)).tw.
117	((kidney or rena? or neph$) adj3 (carcinoma or cancer or neoplas?)).tw.
118	exp Thyroid Neoplasms/
119	Thyroid cancer.mp.
120	thyroid carcinoma.mp.
121	Leukemia/
122	leukemia.mp.
123	leukaemia.mp.
124	exp Colorectal Neoplasms/
125	colorectal cancer.mp.
126.	exp Lymphoma/
127	lymphoma.mp.
128	1 or 2 or 3 or 4 or 5 or 6 or 7or 8 or 9 or 10 or 11 or 12 or 13 or 14 or 15 or 16 or 17 or 18 or 19 or 20 or 21 or 22 or 23 or 24 or 25 or 26 or 27 or 28 or 29 or 30 or 31 or 32 or 33 or 34 or 35 or 36 or 37 or 38 or 39 or 40 or 41 or 42 or 43 or 44 or 45 or 46 or 47 or 48 or 49 or 50 or 51 or 52 or 53 or 54 or 55 or 56 or 57 or 58 or 59 or 60 or 61 or 62 or 63 or 64 or 65 or 66 or 67 or 68 or 69 or 71 or 72 or 73 or 74 or 75 or 76
129	77 or 78 or 79 or 80 or 81 or 82 or 83 or 84 or 85 or 86 or 87 or 88 or 89 or 90 or 91 or 92 or 93 or 94 or 95 or 96 or 97 or 98 or 99 or 100 or 101 or 102 or 103 or 104 or 105 or 106 or 107 or 108 or 109 or 110 or 111 or 112 or 113 or 114 or 115 or 116 or 117 or 118 or 119 or 120 0r 121 or 122 or 123 or 124 or 125 or 126 or 127
130	128 or 129
131	(systematic$ adj2 (review$ or overview)).ti,ab.
132	(systematic$ adj5 review$).tw,sh.
133	meta-analysis.mp. or exp meta-analysis/
134	131 or 132 or 133
135	130 and 134

For peer review only - http://bmjopen.bmj.com/site/about/guidelines.xhtml

Appendix Table 5.3 Search strategy- Medline database- Funtional conditions and pregnancy complications

1.	exp Abortion, Spontaneous/
2.	(stillbirth or still birth).mp.
3.	(recurrent adj3 miscarr$).mp. [mp=title, abstract, original title, name of substance word, subject heading word, floating sub-heading word, keyword heading word, organism supplementary concept word, protocol supplementary concept word, rare disease supplementary concept word, unique identifier, synonyms]
4.	(miscarr$ or early pregnancy loss$).mp. [mp=title, abstract, original title, name of substance word, subject heading word, floating sub-heading word, keyword heading word, organism supplementary concept word, protocol supplementary concept word, rare disease supplementary concept word, unique identifier, synonyms]
5.	exp Stillbirth/
6.	exp Fetal Death/
7.	(f?etal death$ or f?etal demise$).mp.
8.	Hypertension, Pregnancy-Induced/
9.	(gestational hypertension or (pregnancy adj3 hypertensi$)).mp.
10.	(preeclampsia or pre-eclampsia).mp.
11.	exp Pre-Eclampsia/
12.	exp Eclampsia/
13.	(eclampsia or tox?emia).mp.
14.	HELLP.mp.
15.	exp HELLP Syndrome/
16.	placenta accreta.mp.
17.	exp Placenta Accreta/
18.	placenta percreta.mp.
19.	placenta increta.mp.
20.	morbidly adherent placenta.mp.
21.	abnormally invasive placenta.mp.
22.	Placenta$ abruption.mp.
23.	exp Abruptio Placentae/
24.	placenta pr?eia.mp.
25.	exp placenta previa/
26.	low lying placenta.mp.
27.	Hyperemesis Gravidarum.mp.
28.	Hyperemesis Gravidarum/
29.	morning sickness.mp.
30.	exp Morning Sickness/
31.	(((pregnan$ or gestation$ or prenatal$ or antenatal$ or pre-natal$ or ante-natal$ or maternal$) adj2 diabet$) or gestational diabetes).mp.
32.	exp Diabetes, Gestational/
33.	GDM.mp.
34.	ectopic pregnancy.mp.
35.	exp Pregnancy, Ectopic/

For peer review only - http://bmjopen.bmj.com/site/about/guidelines.xhtml

36.	((tub$$ adj3 pregnanc$) or (cornual adj3 pregnanc$) or (heterotopic adj3 pregnanc$) or (abdomin$ adj3 pregnanc$) or (extrauterine adj3 pregnanc$) or (interstitial adj3 pregnanc$) or (cervi$ adj3 pregnanc$) or (ovar$ adj3 pregnanc$) or (cesarean scar adj3 pregnanc$)).mp.
37.	exp Gestational Trophoblastic Disease/
38.	gestational trophoblastic.mp.
39.	exp Hydatidiform Mole/
40.	((hydatid? adj2 mole?) or (molar adj2 pregnanc?)).mp.
41.	exp Choriocarcinoma/ or choriocarcinoma.mp.
42.	exp Pregnancy, Multiple/
43.	exp Pregnancy, Multiple/ or ((pregnanc* or gestation*) adj (twin* or triplet* or quadruplet* or quintuplet* or multiple or multi?f?et*)).mp.
44.	(Monochorionic or dichorionic).mp.
45.	exp Postpartum Hemorrhage/
46.	(postpartum hemorrhage or post partum hemorrhage or postpartum haemorrhage or post partum haemorrhage).ti,ab.
47.	obstetric haemorrhage.mp.
48.	obstetric labor, premature.mp.
49.	exp Obstetric Labor, Premature/
50.	(premature labor or premature labour or preterm labor or preterm labour or preterm birth).mp.
51.	exp Cesarean Section, Repeat/ or cesarean.mp.
52.	exp Cesarean Section/
53.	(caesarean or cesarean or caesarian or cesarian or cesarien or caesarien or c-section or c section).mp.
54.	exp Extraction, Obstetrical/
55.	exp Obstetrical Forceps/
56.	((operative or instrumental or assisted or forcep* or ventouse* or vacuum*) adj1 (deliver* or birth*)).mp.
57.	low birth weight.mp.
58.	exp Infant, Low Birth Weight/
59.	(low birth weight* adj4 very low birth weight*).mp.
60.	exp Infant, Small for Gestational Age/
61.	small for gestational age.mp.
62.	(small adj3 gestational age).mp.
63.	(intra?uterine growth adj2 (restriction* or retardation)).mp.
64.	iugr.ti,ab.
65.	fetal growth retardation.mp.
66.	exp Fetal Growth Retardation/
67.	foetal growth retardation.mp.
68.	(fetal growth adj2 (restriction? or retardation)).mp.
69.	(foetal growth adj2 (restriction? or retardation)).mp.
70.	((postpartum* or post partum* or post-partum* or postnatal* or post natal* or post-natal* or perinatal* or peri natal* or peri-natal* or puerp*) and (depress* or dysthymi* or adjustment disorder* or mood disorder* or affective disorder*)).mp.
71.	(((third or fourth or 3rd or 4th) adj degree) and tear*).mp.

For peer review only - http://bmjopen.bmj.com/site/about/guidelines.xhtml

72.	(((anal near adj2 sphincter) or (rectal adj mucosa) or rectum or (anal adj epithelium) or anus or (recto?vaginal adj2 fistulae) or (anorectal adj mucosa) or (anal adj skin)) and (tear* or injur* or damage* or lacerat* or rupture* or trauma)).mp.
73.	((obstetric* and anal and sphincter and injur*) or (anal and sphincter and injur*)).mp.
74.	(exp Pregnancy/ or exp Obstetrics/ or (pregnan* or obstetric*).mp.) and (exp Cholestasis/ or exp Cholestasis, Intrahepatic/)
75	pregnancy complication*.mp.
76	exp Pregnancy Complications/
77	exp FIBROMYALGIA/
78.	exp Low Back Pain/
79	exp Back Pain/
80	chronic back pain.mp.
81	interstitial cystitis.mp.
82	exp Cystitis, Interstitial/
83	irritable bowel syndrome.mp.
84	exp Irritable Bowel Syndrome/
85	vulvodynia.mp.
86	exp VULVODYNIA/
87	tension headache.mp.
88.	exp Tension -Type Headache/
89	1 or 2 or 3 or 4 or 5 or 6 or 7or 8 or 9 or 10 or 11 or 12 or 13 or 14 or 15 or 16 or 17 or 18 or 19 or 20 or 21 or 22 or 23 or 24 or 25 or 26 or 27 or 28 or 29 or 30 or 31 or 32 or 33 or 34 or 35 or 36 or 37 or 38 or 39 or 40 or 41 or 42 or 43 or 44 or 45 or 46 or 47 or 48 or 49 or 50 or 51 or 52 or 53 or 54 or 55 or 56 or 57 or 58 or 59 or 60 or 61 or 62 or 63 or 64 or 65 or 66 or 67 or 68 or 69 or 71 or 72 or 73 or 74 or 75 or 76
90	77 or 78 or 79 or 80 or 81 or 82 or 83 or 84 or 85 or 86 or 87 or 88
91	89 and 90
92	(systematic$ adj2 (review$ or overview)).ti,ab.
93	(systematic$ adj5 review$).tw,sh.
94	meta-analysis.mp. or exp meta-analysis/
95	92 or 93 or 94
96	91 and 95

<u>Appendix Table 5.4 search strategy-mental health conditions and pregnancy complications</u>

For peer review only - http://bmjopen.bmj.com/site/about/guidelines.xhtml

	Search term
1	pregnancy complication*.mp. or exp Pregnancy Complications/
2	exp Abortion, Spontaneous/ or ((recurrent adj3 miscarr$) or miscarr$ or early pregnancy loss$).mp.
3	(stillbirth or still birth).mp. or exp Stillbirth/ or exp Fetal Death/ or (f?etal death$ or f?etal demise$).mp.
4	Hypertension, Pregnancy-Induced/ or (gestational hypertension or (pregnancy adj3 hypertensi$)).mp.
5	(preeclampsia or pre-eclampsia).mp. or exp Pre-Eclampsia/
6	exp Eclampsia/ or (eclampsia or tox?emia).mp.
7	HELLP.mp. or exp HELLP Syndrome/
8	placenta accreta.mp. or exp Placenta Accreta/ or placenta percreta.mp. or placenta increta.mp. or morbidly adherent placenta.mp. or abnormally invasive placenta.mp.
9	Placenta$ abruption.mp. or exp Abruptio Placentae/
10	placenta pr?eia.mp. or exp placenta previa/ or exp low lying placenta/
11	Hyperemesis Gravidarum.mp. or Hyperemesis Gravidarum/ or morning sickness.mp. or exp Morning Sickness/
12	(((pregnan$ or gestation$ or prenatal$ or antenatal$ or pre-natal$ or ante-natal$ or maternal$) adj2 diabet$) or gestational diabetes).mp. or exp Diabetes, Gestational/ or GDM.mp.
13	ectopic pregnancy.mp. or exp Pregnancy, Ectopic/ or ((tub$$ adj3 pregnanc$) or (cornual adj3 pregnanc$) or (heterotopic adj3 pregnanc$) or (abdomin$ adj3 pregnanc$)

For peer review only

For peer review only - http://bmjopen.bmj.com/site/about/guidelines.xhtml

	or (extrauterine adj3 pregnanc$) or (interstitial adj3 pregnanc$) or (cervi$ adj3 pregnanc$) or (ovar$ adj3 pregnanc$) or (cesarean scar adj3 pregnanc$)).mp.
14	exp Gestational Trophoblastic Disease/ or gestational trophoblastic.mp. or exp Hydatidiform Mole/ or ((hydatid? adj2 mole?) or (molar adj2 pregnanc?)).mp. or exp Choriocarcinoma/ or choriocarcinoma.mp.
15	exp Pregnancy, Multiple/ or ((pregnanc* or gestation*) adj (twin* or triplet* or quadruplet* or quintuplet* or multiple or multi?f?et*)).mp. or (Monochorionic or dichorionic).mp.
16	exp Postpartum Hemorrhage/ or (postpartum hemorrhage or post partum hemorrhage or postpartum haemorrhage or post partum haemorrhage).ti,ab. or obstetric haemorrhage.mp.
17	obstetric labor, premature.mp. or exp Obstetric Labor, Premature/ or (premature labor or premature labour or preterm labor or preterm labour or preterm birth).mp.
18	exp Cesarean Section, Repeat/ or cesarean.mp. or exp Cesarean Section/ or (caesarean or cesarean or caesarian or cesarian or cesarien or caesarien or c-section or c section).mp. or exp Extraction, Obstetrical/ or exp Obstetrical Forceps/ or ((operative or instrumental or assisted or forcep* or ventouse* or vacuum*) adj1 (deliver* or birth*)).mp.
19	low birth weight.mp. or exp Infant, Low Birth Weight/ or (low birth weight* adj4 very low birth weight*).mp.
20	exp Infant, Small for Gestational Age/ or small for gestational age.mp. or (small adj3 gestational age).mp.
21	(intra?uterine growth adj2 (restriction* or retardation)).mp. or iugr.ti,ab. or fetal growth retardation.mp. or exp Fetal Growth Retardation/ or (fetal growth adj2 (restriction? or retardation)).mp.
22	((postpartum* or post partum* or post-partum* or postnatal* or post natal* or post-natal* or perinatal* or peri natal* or peri-natal* or puerp*) and (depress* or dysthymi* or adjustment disorder* or mood disorder* or affective disorder*)).mp.

For peer review only - http://bmjopen.bmj.com/site/about/guidelines.xhtml

23	(((postpartum* or post partum* or post-partum* or postnatal* or post natal* or post-natal or perinatal* or peri natal* or peri-natal* or puerp*) and (psychos#s or psychotic)) or psychosis after childbirth).mp.
24	((((third or fourth or 3rd or 4th) adj degree) and tear*) or (((anal near adj2 sphincter) or (rectal adj mucosa) or rectum or (anal adj epithelium) or anus or (recto?vaginal adj2 fistulae) or (anorectal adj mucosa) or (anal adj skin)) and (tear* or injur* or damage* or lacerat* or rupture* or trauma)) or ((obstetric* and anal and sphincter and injur*) or (anal and sphincter and injur*))).mp.
25	(exp Pregnancy/ or exp Obstetrics/ or (pregnan* or obstetric*).mp.) and (exp Cholestasis/ or exp Cholestasis, Intrahepatic/)
26	pelvic girdle pain.mp. or exp Pelvic Girdle Pain/ or (symphysis pubis adj3 (pain$ or dysfunction$)).mp. or Pubic Symphysis Diastasis.mp. or exp Pubic Symphysis Diastasis/ or Sacroiliac joint dysfunction.mp. or PGP.mp.
27	1 or 2 or 3 or 4 or 5 or 6 or 7 or 8 or 9 or 10 or 11 or 12 or 13 or 14 or 15 or 16 or 17 or 18 or 19 or 20 or 21 or 22 or 23 or 24 or 25 or 26
28	((affective and non-affective) or (psychos*s or psychotic) or (bipolar* or bi-polar* or mania* or manic-depress**) or (schizophrenia* or schizo-affective or schizophrenia*) or (delusion* or paranoi*)).mp.
29	(depress* or dysthymi* or mood disorder* or affective disorder* or distress* or neuros*).mp.
30	(anxiet* or obsessive compulsive disorder* or OCD* PTSD or post-traumatic stress disorder or panic* or neurosis* or neurotic* or adjustment disorder*).mp.
31	(systematic review or meta-analysis).mp.
32	29 or 30
33	27 and 28

For peer review only - http://bmjopen.bmj.com/site/about/guidelines.xhtml

For peer review only

34	27 and 28 and 31
35	27 and 32
36	27 and 31 and 32

Appendix Table 5.5 Search strategy- Medline database-metabolic conditions and pregnancy complications

	**Search terms**
1	pregnancy complication*.mp. or exp Pregnancy Complications/
2	exp Abortion, Spontaneous/ or ((recurrent adj3 miscarr$) or miscarr$ or early pregnancy loss$).mp.
3	(stillbirth or still birth).mp. or exp Stillbirth/ or exp Fetal Death/ or (f?etal death$ or f?etal demise$).mp.
4	Hypertension, Pregnancy-Induced/ or (gestational hypertension or (pregnancy adj3 hypertensi$)).mp.
5	(preeclampsia or pre-eclampsia).mp. or exp Pre-Eclampsia/
6	exp Eclampsia/ or (eclampsia or tox?emia).mp.
7	HELLP.mp. or exp HELLP Syndrome/
8	placenta accreta.mp. or exp Placenta Accreta/ or placenta percreta.mp. or placenta increta.mp. or morbidly adherent placenta.mp. or abnormally invasive placenta.mp.
9	Placenta$ abruption.mp. or exp Abruptio Placentae/
10	placenta pr?eia.mp. or exp placenta previa/ or exp low lying placenta/
11	Hyperemesis Gravidarum.mp. or Hyperemesis Gravidarum/ or morning sickness.mp. or exp Morning Sickness/

For peer review only - http://bmjopen.bmj.com/site/about/guidelines.xhtml

12	(((pregnan$ or gestation$ or prenatal$ or antenatal$ or pre-natal$ or ante-natal$ or maternal$) adj2 diabet$) or gestational diabetes).mp. or exp Diabetes, Gestational/ or GDM.mp.
13	ectopic pregnancy.mp. or exp Pregnancy, Ectopic/ or ((tub$$ adj3 pregnanc$) or (cornual adj3 pregnanc$) or (heterotopic adj3 pregnanc$) or (abdomin$ adj3 pregnanc$) or (extrauterine adj3 pregnanc$) or (interstitial adj3 pregnanc$) or (cervi$ adj3 pregnanc$) or (ovar$ adj3 pregnanc$) or (cesarean scar adj3 pregnanc$)).mp.
14	exp Gestational Trophoblastic Disease/ or gestational trophoblastic.mp. or exp Hydatidiform Mole/ or ((hydatid? adj2 mole?) or (molar adj2 pregnanc?)).mp.
15	exp Choriocarcinoma/ or choriocarcinoma.mp.
16	exp Pregnancy, Multiple/ or ((pregnanc* or gestation*) adj (twin* or triplet* or quadruplet* or quintuplet* or multiple or multi?f?et*)).mp. or (Monochorionic or dichorionic).mp.
17	exp Postpartum Hemorrhage/ or (postpartum hemorrhage or post partum hemorrhage or postpartum haemorrhage or post partum haemorrhage).ti,ab. or obstetric haemorrhage.mp.
18	obstetric labor, premature.mp. or exp Obstetric Labor, Premature/ or (premature labor or premature labour or preterm labor or preterm labour or preterm birth).mp.
19	exp Cesarean Section, Repeat/ or cesarean.mp. or exp Cesarean Section/ or (caesarean or cesarean or caesarian or cesarian or cesarien or caesarien or c-section or c section).mp.
20	exp Extraction, Obstetrical/ or exp Obstetrical Forceps/ or ((operative or instrumental or assisted or forcep* or ventouse* or vacuum*) adj1 (deliver* or birth*)).mp.
21	low birth weight.mp. or exp Infant, Low Birth Weight/ or (low birth weight* adj4 very low birth weight*).mp.
22	exp Infant, Small for Gestational Age/ or small for gestational age.mp. or (small adj3 gestational age).mp.
23	(intra?uterine growth adj2 (restriction* or retardation)).mp. or iugr.ti,ab.

For peer review only - http://bmjopen.bmj.com/site/about/guidelines.xhtml

24	fetal growth retardation.mp. or exp Fetal Growth Retardation/ or (fetal growth adj2 (restriction? or retardation)).mp.
25	postpartum depression.mp. or exp Depression, Postpartum/
26	((postpartum* or post partum* or post-partum* or postnatal* or post natal* or post-natal* or perinatal* or peri natal* or peri-natal* or puerp*) and (depress* or dysthymi* or adjustment disorder* or mood disorder* or affective disorder*)).mp.
27	(((postpartum* or post partum* or post-partum* or postnatal* or post natal* or post-natal or perinatal* or peri natal* or peri-natal* or puerp*) and (psychos#s or psychotic)) or psychosis after childbirth).mp.
28	(((third or fourth or 3rd or 4th) adj degree) and tear*).mp.
29	(((anal near adj2 sphincter) or (rectal adj mucosa) or rectum or (anal adj epithelium) or anus or (recto?vaginal adj2 fistulae) or (anorectal adj mucosa) or (anal adj skin)) and (tear* or injur* or damage* or lacerat* or rupture* or trauma)).mp. [mp=title, book title, abstract, original title, name of substance word, subject heading word, floating sub-heading word, keyword heading word, organism supplementary concept word, protocol supplementary concept word, rare disease supplementary concept word, unique identifier, synonyms]
30	((obstetric* and anal and sphincter and injur*) or (anal and sphincter and injur*)).mp.
31	(exp Pregnancy/ or exp Obstetrics/ or (pregnan* or obstetric*).mp.) and (exp Cholestasis/ or exp Cholestasis, Intrahepatic/)
32	1 or 2 or 3 or 4 or 5 or 6 or 7 or 8 or 9 or 10 or 11 or 12 or 13 or 14 or 15 or 16 or 17 or 18 or 19 or 20 or 21 or 22 or 23 or 24 or 25 or 26 or 27 or 28 or 29 or 30 or 31
33	exp Diabetes Mellitus, Type 2/ or diabetes type 2.mp. or Type 2/ or (MODY or NIDDM or T2DM or T2D).mp. or (non insulin* depend* or noninsulin* depend* or noninsulin?depend* or non insulin?depend*).mp. or ((typ? 2 or typ? II or typ?2 or typ?II) adj3 diabet*).mp. or (((late or adult* or matur* or slow or stabl*) adj3 onset) and diabet*).mp.
34	exp hypertension/ or hypertens$.mp. or exp blood pressure/ or (blood pressure or bloodpressure).mp.

For peer review only - http://bmjopen.bmj.com/site/about/guidelines.xhtml

35	33 or 34
36	systematic review.mp.
37	(systematic$ adj2 (review$ or overview)).ti,ab.
38	(systematic$ adj5 review$).tw,sh.
39	meta-analysis.mp. or exp meta-analysis/
40	36 or 37 or 38 or 39
41	32 and 35 and 40

References

1. Legistation UK-https://www.legislation.gov.uk/ukpga/1992/29/section/1.
2. National Institute for Health and Care Excellence. NICE Guideline [NG133]: Hypertension in pregnancy: diagnosis and management. 2019. https://www.nice.org.uk/guidance/ng133/chapter/Recommendations#management-of-gestational-hypertension.
3. Royal college of obstetrician and gynaecologists,Placenta praevia, placenta accreta and

vasa praevia,2018

https://www.rcog.org.uk/globalassets/documents/patients/patient-information-leaflets/pregnancy/pi-placenta-praevia-placenta-accreta-and-vasa-praevia.pdf.
4. Workalemahu T, Enquobahrie DA, Gelaye B, Thornton TA, Tekola-Ayele F, Sanchez SE, et al. Abruptio placentae risk and genetic variations in mitochondrial biogenesis and oxidative phosphorylation: replication of a candidate gene association study. American journal of obstetrics and gynecology. 2018;219(6):617. e1-. e17.
5. Martinelli KG, Garcia ÉM, Santos Neto ETd, Gama SGNd. Advanced maternal age and its association with placenta praevia and placental abruption: a meta-analysis. Cadernos de saude publica. 2018;34.
6. Royal college of obstetrician and gynaecologists,The Management of Nausea andVomiting

of Pregnancy and Hyperemesis Gravidarum

Green-top Guideline No. 69,2016

https://www.rcog.org.uk/globalassets/documents/guidelines/green-top-guidelines/gtg69-hyperemesis.pdf.
7. BMJ-Best practice,Gestational diabetes mellitus,2021

https://bestpractice.bmj.com/topics/en-gb/665.
8. Royal college of obstetrician and gynaecologists,,Ectopic pregnancy,2016

https://www.rcog.org.uk/en/patients/patient-leaflets/ectopic-pregnancy/.
9. Royal college of obstetrician and gynaecologists,,Molar pregnancy and gestational trophoblastic disease,2019

https://www.rcog.org.uk/en/patients/patient-leaflets/gestational-trophoblastic-disease-gtd/#:~:text=Molar%20pregnancy%20is%20an%20abnormal,from%20your%20uterus%20(womb).

For peer review only - http://bmjopen.bmj.com/site/about/guidelines.xhtml

10. Gestational trophoblastic disease,Cancer research UK https://www.cancerresearchuk.org/about-cancer/gestational-trophoblastic-disease-gtd accessed in Feb,2022.
11. Royal college of obstetrician and gynaecologists,,Multiple pregnancy: having more than one baby,2021

https://www.rcog.org.uk/en/patients/patient-leaflets/multiple-pregnancy-having-more-than-one-baby/.
12. Royal college of obstetrician and gynaecologists,Heavy bleeding after birth (postpartum haemorrhage,,2016

https://www.rcog.org.uk/en/patients/patient-leaflets/heavy-bleeding-after-birth-postpartum-haemorrhage/.
13. World health organisation

Preterm birth,2018

https://www.who.int/news-room/fact-sheets/detail/preterm-birth.
14. National maternal and Perinatal Audit- https://maternityaudit.org.uk/Audit/Charting/ClinicalByMeasure. 2017/2018.
15. World health organisation

Low birth weight,2019

-https://www.who.int/data/nutrition/nlis/info/low-birth-weight#:~:text=Low%20birth%20weight%20has%20been,2500%20grams%20(5.5%20pounds).
16. Royal college of obstetrician and gynaecologists,Small-for-Gestational-Age Fetus, Investigation and Management (Green-top Guideline No. 31),2013

https://www.rcog.org.uk/en/guidelines-research-services/guidelines/gtg31/.
17. National institute of health and care excellence,Depression - antenatal and postnatal,2020,https://cks.nice.org.uk/topics/depression-antenatal-postnatal/.
18. National institute of health and care excellence,Antenatal and postnatal mental health: clinical management and service guidance,2020, https://www.nice.org.uk/guidance/cg192/chapter/Introduction.
19. Royal college of obstetrician and gynaecologists Care of a third- or fourth-degre, e tear that occurred during childbirth (OASI),2019 https://www.rcpsych.ac.uk/mental-health/problems-disorders/postpartum-psychosis.
20. Royal college of obstetrician and gynaecologists ,Obstetric Cholestasis,(Green-top Guideline No. 43),2014

https://www.rcog.org.uk/globalassets/documents/patients/patient-information-leaflets/pregnancy/pi-obstetric-cholestasis.pdf

21. Pelvic girdle pain and pregnancy -<https://www.rcog.org.uk/for-the-public/browse-all-patient-information-leaflets/pelvic-girdle-pain-and-pregnancy/-assessed 27/05/2022>.

For peer review only - http://bmjopen.bmj.com/site/about/guidelines.xhtml
